# Supplementary material for: In situ orderly self-assembly strategy affording NIR-II-J-aggregates for in vivo imaging and surgical navigation
Source: Nat Commun. 2023 Apr 3;14:1843. doi: 10.1038/s41467-023-37586-7 (PMC10070396; doi:10.1038/s41467-023-37586-7)
Supplement: Supplementary file 1 — Supplementary Information [file 41467_2023_37586_MOESM1_ESM.pdf]

Supplementary information

In Situ Orderly Self-assembly Strategy Affording NIR-II-J-Aggregates for In Vivo Imaging and Surgical Navigation

Zhe Li[1], Ping-Zhao Liang[1], Li Xu[1], Xing-Xing Zhang[1], Ke Li[1], Qian Wu[1], Xiao-Feng Lou[1], Tian-Bing Ren[1], Lin Yuan[1], and Xiao-Bing Zhang[1,2]*

[1]State Key Laboratory of Chemo/Biosensing and Chemometrics, College of Chemistry and Chemical Engineering, Hunan University, Changsha 410082, China
[2]Lead Contact
*Correspondence: xbzhang@hnu.edu.cn

Table of contents：

1. Materials and instruments.. S3

2. Additional experimental data. S4

3. Synthesis of compounds... S18

4. MS Spectra and NMR spectra. S32

5. Supplementary References S70

1. Materials and instruments

Materials and instruments. Unless otherwise stated, all reagents were purchased from commercial suppliers and used without further purification. All commercial organic compounds were purchased from Bide Pharmatech Ltd.1,2-dimyristoyl-sn-glycero-3-phosphocholine (DMPC) were purchased from Xi'an ruixi Biological Technology Co., Ltd. Filter membrane (220 um) is purchased from Titan. Solvents were purified by standard methods prior to use. Twice-distilled water was used throughout all experiments. Mass spectra were performed using an Agilent 1200-6520 Q-TOF mass spectrometer system operating in a MALDI-TOF mode and LCQ Advantage ion trap mass spectrometer (Thermo Finnigan). NMR spectra were recorded on Bruker-400, using TMS as the internal standard. UV–Vis spectra were recorded on a UV-1800 spectrophotometer (Shimadzu Corporation, Japan). Photoluminescence spectra were recorded on an Edinburgh Instruments FLS-1000 fluorescence spectrometer. The pH measurements were carried out on a PHS-3C pH meter (INESA instrument). TLC analysis was performed on silica gel plates and column chromatography was conducted over silica gel (mesh 200–300) columns, obtained from the Yantai Jiangyou silica gel Development Company Limited. Dynamic light scattering (DLS) was measured on Malvern Zetasizer Nano ZS90 (Malvern). Mice were purchased from Hunan Slake Jingda Laboratory Animal Co., Ltd (China). The AFM characterization was conducted by Bruker Multimode V8 Scanning Probe Microscopy (Bruker, German). The SAXS characterization was conducted by xeuss 2.0 (Xenocs, France). All statistical graph, absorption spectrum and fluorescent spectra were analyzed with OriginLab 2019. NMR files were analyzed with MestReNova. Mass spectrum files were analyzed with flexAnalysis.

2. Additional experimental data.

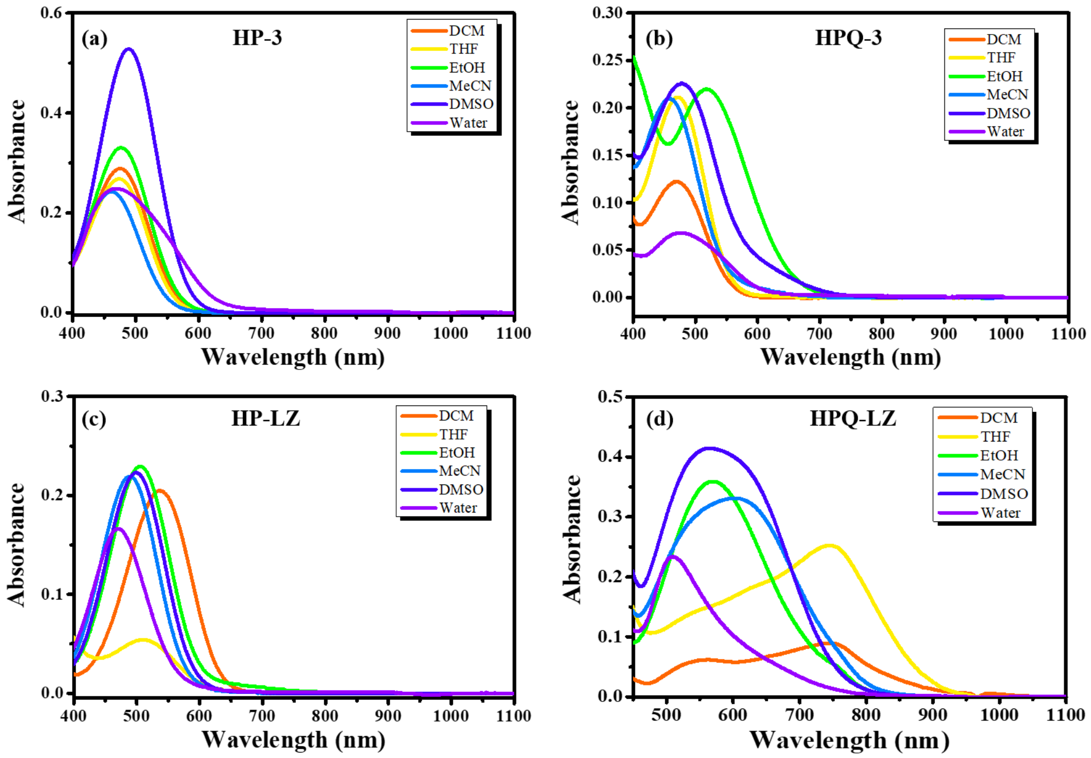

Supplementary Figure 1 | Absorbance spectrum of **HP-3 (a)**, **HPQ-3 (b)**, **HP-LZ (c)**, **HPQ-LZ (d)** in different solvents.

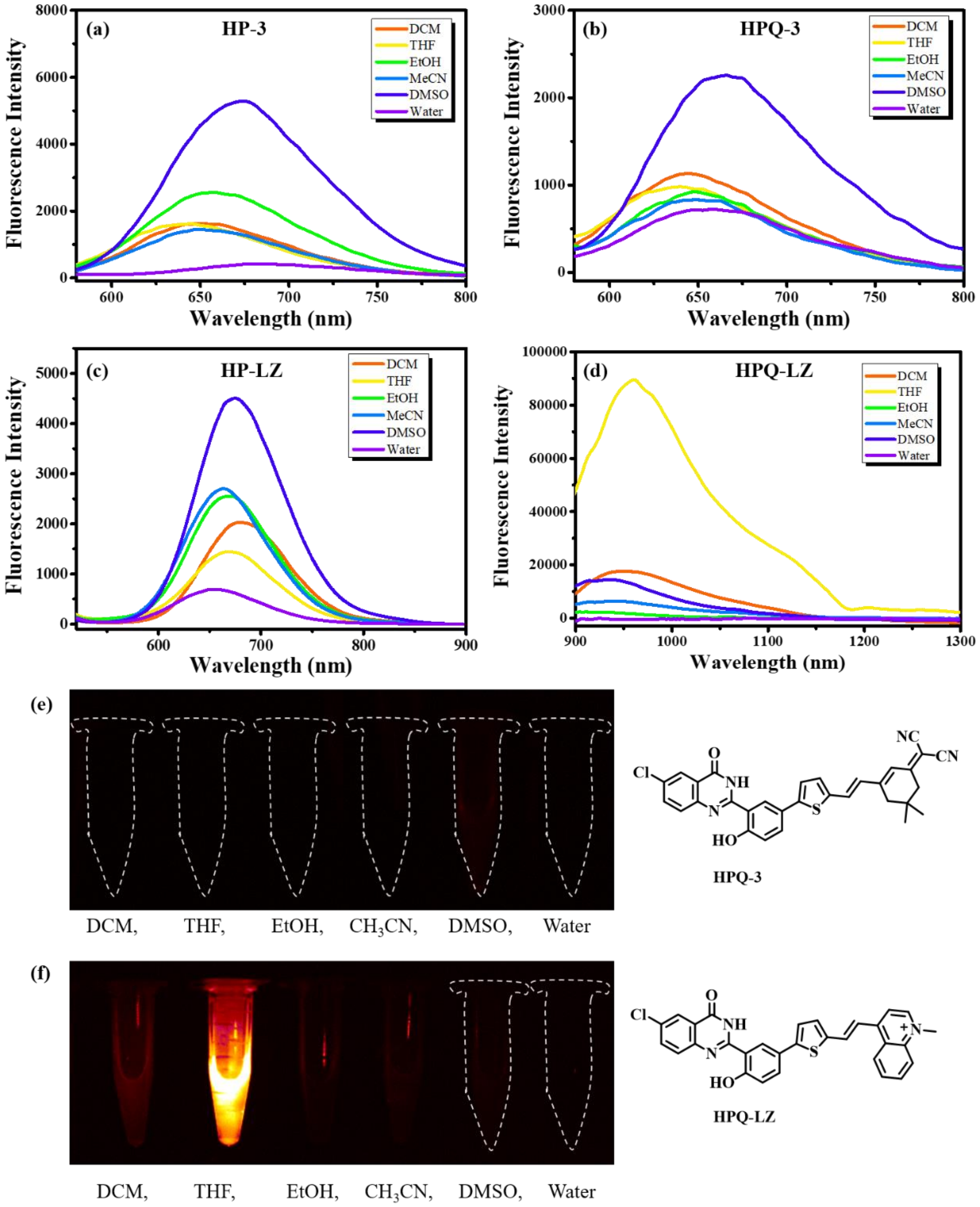

Supplementary Figure 2 | Fluorescence spectrum of **HP-3 (a)**, **HPQ-3 (b)**, **HP-LZ (c)**, **HPQ-LZ (d)** in different solvents, a, b: λ_{ex} = 500 nm, c: λ_{ex} = 550 nm, d: λ_{ex} = 808 nm. NIR-II fluorescence of **HPQ-3 (e)** and **HPQ-LZ (f)** in different solvents, λ_{ex} = 808 nm, Collection channel: 1000-1700 nm.

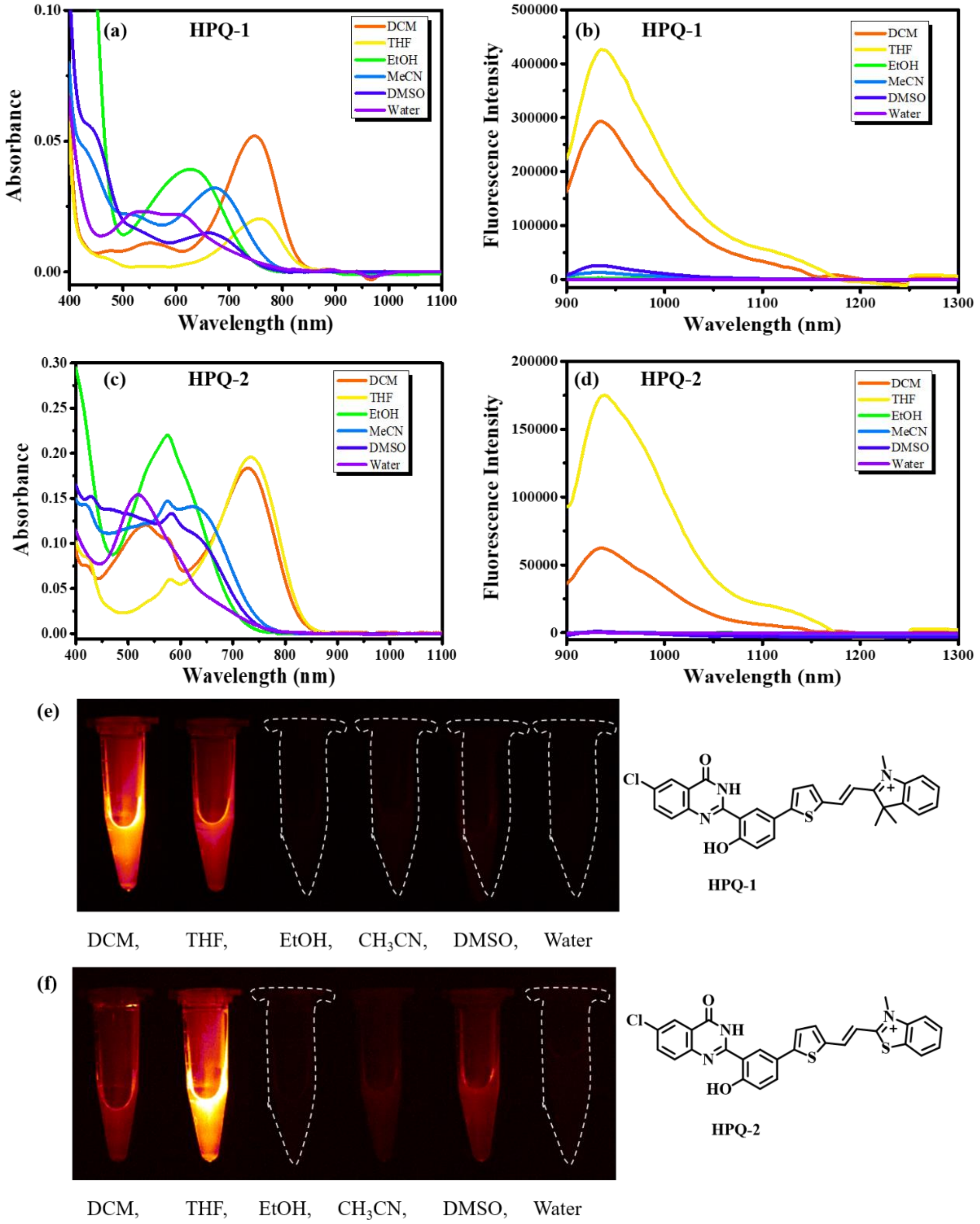

Supplementary Figure 3 | Absorbance spectrum of **HPQ-1 (a)**, **HPQ-2 (c)** in different solvents, NIR-II fluorescence spectrum of **HPQ-1 (b)**, **HPQ-2 (d)** in different solvents, λ_{ex} = 808 nm. NIR-II fluorescence of **HPQ-1 (e)**, **HPQ-2 (f)** in different solvents, λ_{ex} = 808 nm, Collection channel: 1000-1700 nm.

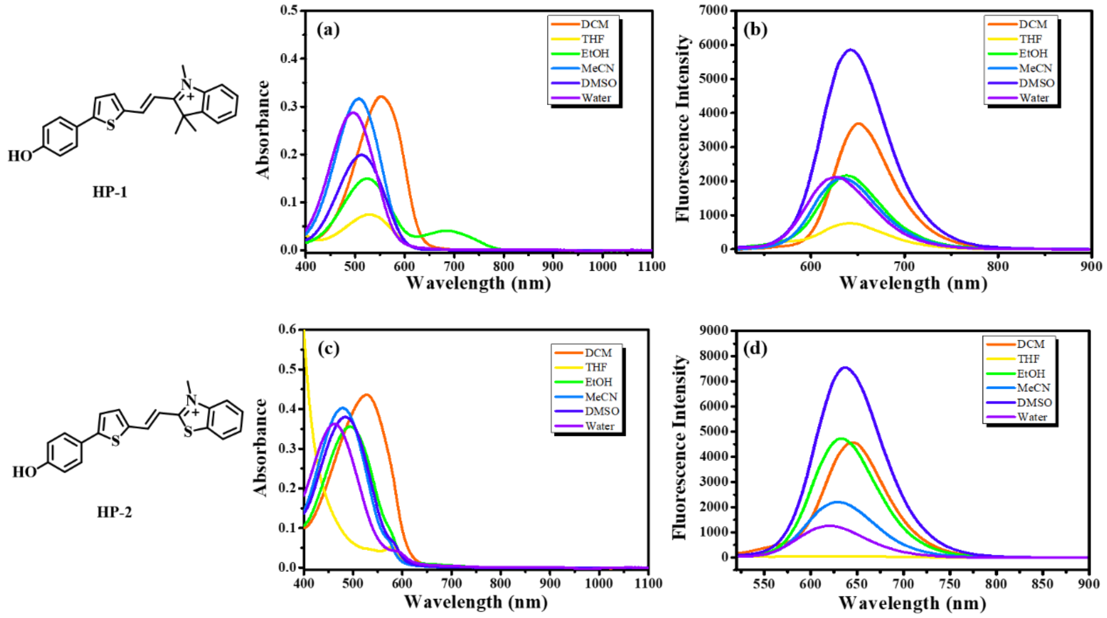

Supplementary Figure 4 | Absorbance spectrum of **HP-1 (a)**, **HP-2 (c)** in different solvents, fluorescence spectrum of **HP-1 (b)**, **HP-2 (d)** in different solvents, $\lambda_{ex} = 500$ nm.

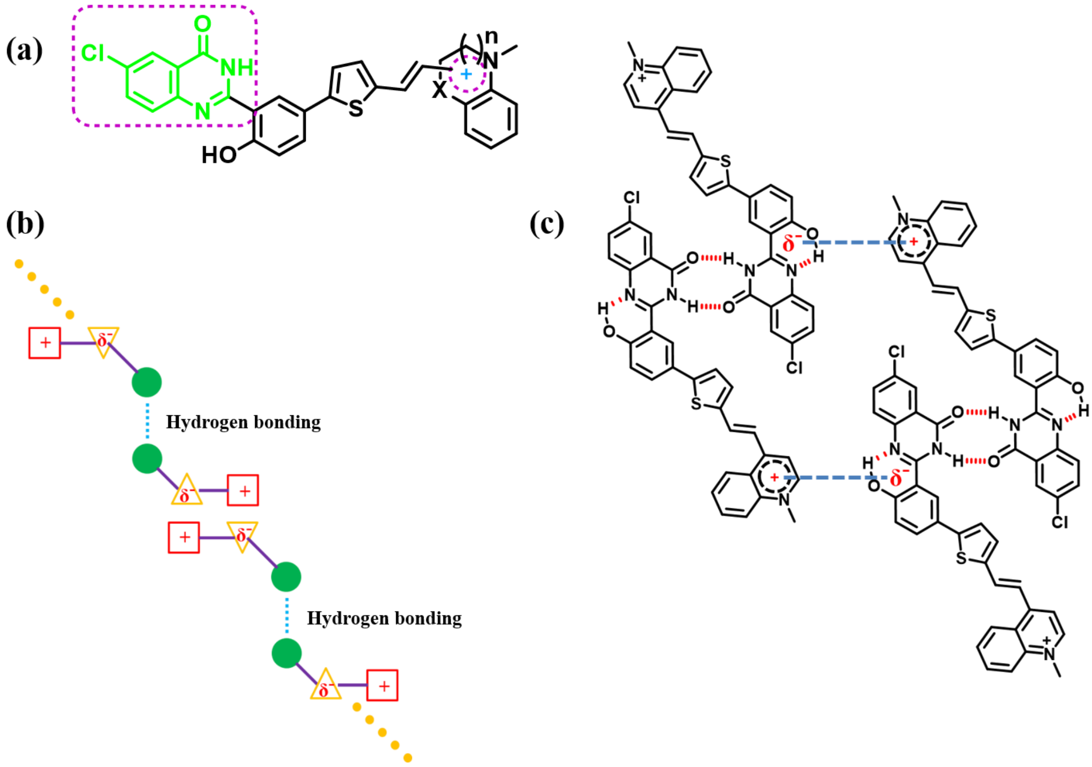

Supplementary Figure 5 | (a) Different modules of the NIR-II-J aggregate. Speculative molecular stacking method, (**b)** Simplified schematic, **(c)** Complete schematic, **HPQ-LZ** as an example.

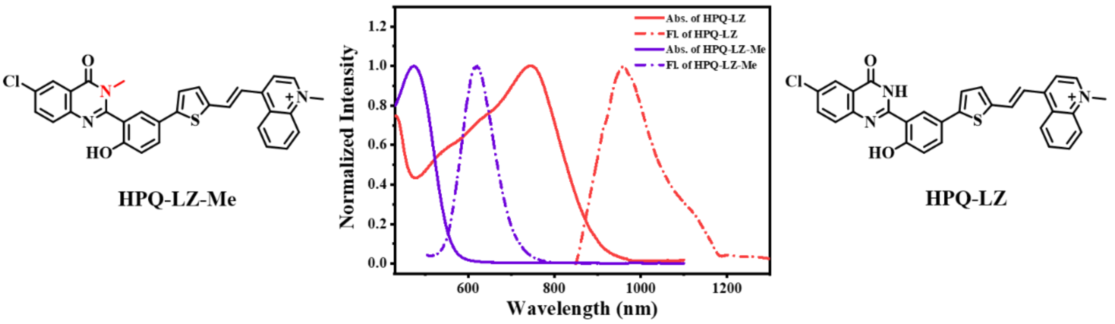

Supplementary Figure 6 | Normalized absorbance and fluorescence spectrum of **HPQ-LZ-Me** and **HPQ-LZ** in THF, λ_{ex} = 450 nm for **HPQ-LZ-Me**, λ_{ex} = 808 nm for **HPQ-LZ**.

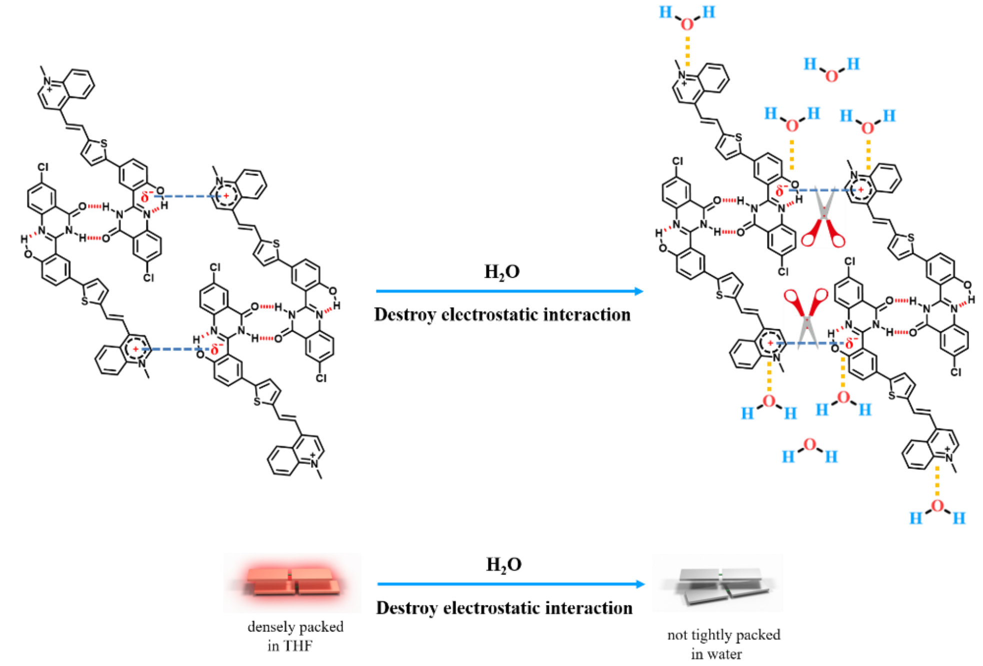

Supplementary Figure 7 | Schematic diagram that water destroys electrostatic interaction.

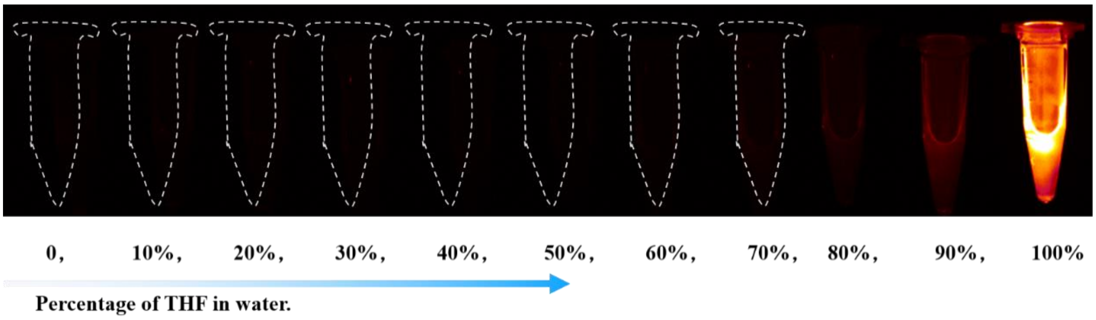

Supplementary Figure 8 | NIR-II fluorescence of **HPQ-LZ** in different ratios of THF in water, λ_{ex} = 808 nm, Collection channel: 1000-1700 nm, In vitro imaging.

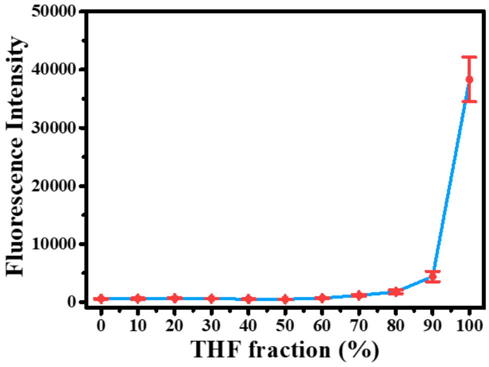

Supplementary Figure 9 | The line chart For **Supplementary Figure 8**. Data were presented as mean ± s.d. derived from n = 3 independent biological samples.

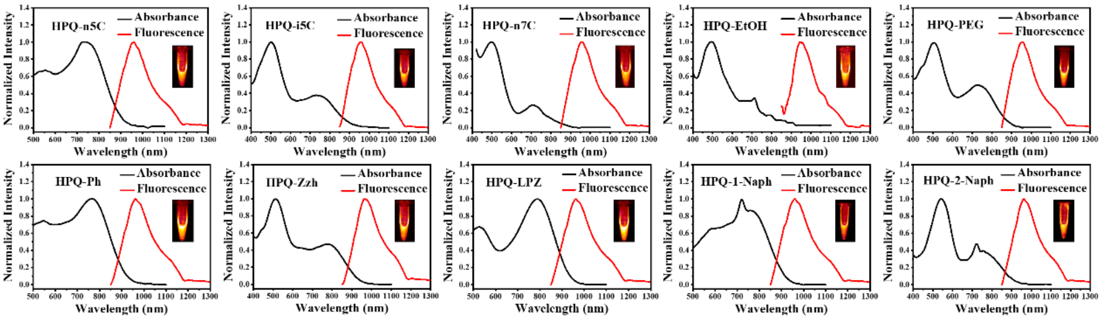

Supplementary Figure 10 | Normalized absorbance and fluorescence spectrum of all HPQ in THF. The vignettes are NIR-II fluorescence of different HPQ in THF, λ_{ex} = 808 nm, Collection channel: 1000-1700 nm.

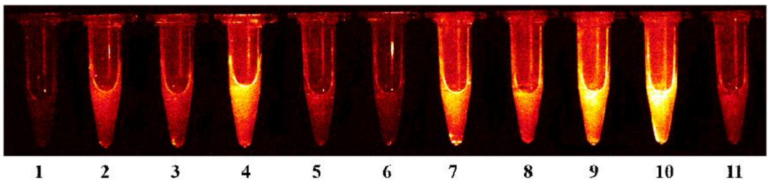

Supplementary Figure 11 | NIR-II fluorescence of all HPQ (20 μM) in DPBS+1 % Tween, (1) **HPQ-LZ**, (2) **HPQ-i5C**, (3) **HPQ-n5C**, (4) **HPQ-n7C**, (5) **HPQ-EtOH**, (6) **HPQ-PEG**, (7) **HPQ-Ph**, (8) **HPQ-LPZ**, (9) **HPQ-Zzh**, (10) **HPQ-1-Naph**, (11) **HPQ-2-Naph**, respectively, λ_{ex} = 808 nm, Collection channel: 1000-1700 nm, in vitro imaging.

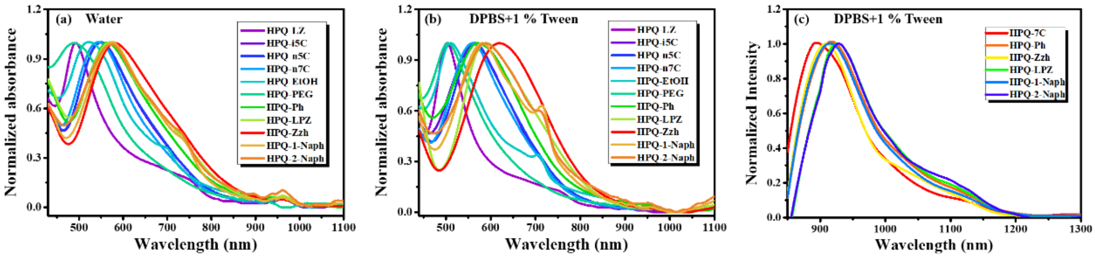

Supplementary Figure 12 | (a) Normalized absorbance spectrum of all HPQ in water. **(b)** Normalized absorbance spectrum of all HPQ in DPBS+1 % Tween. **(c)** Normalized fluorescence spectrum of HPQs in DPBS+1 % Tween, λ_{ex} = 808 nm.

Compounds	HPQ-LZ	HPQ-n5C	HPQ-i5C	HPQ-n7C	HPQ-EtOH	HPQ-PEG
Q_Y	< 0.01%	< 0.01%	< 0.01%	0.012%	< 0.01%	< 0.01%
**Compounds**	**HPQ-Ph**	**HPQ-LPZ**	**HPQ-Zzh**	**HPQ-1-Naph**	**HPQ-2-Naph**	
Q_Y	0.025%	0.014%	0.037%	0.049%	< 0.01%	

Supplementary Table 1. Fluorescence quantum yield (Q_Y) of different HPQs in DPBS + 1% Tween. Reference fluorophore: **IR-1061** (Q_Y = 0.32 % in DCM, excited at 808 nm)[1].

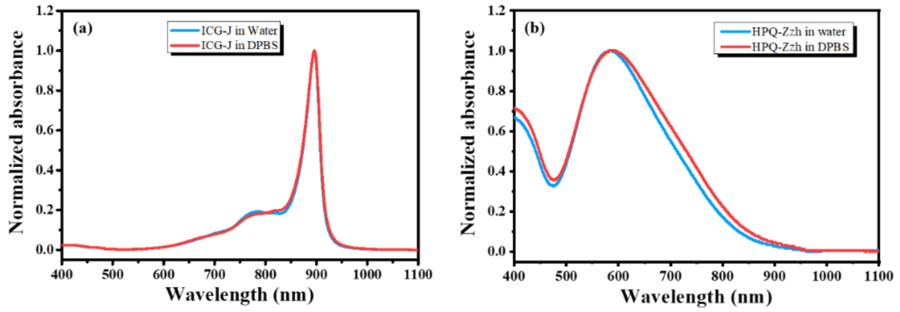

Supplementary Figure 13 | Normalized absorbance spectrum of **(a)** ICG-J-aggregates and **(b) HPQ-Zzh** in different solvent.

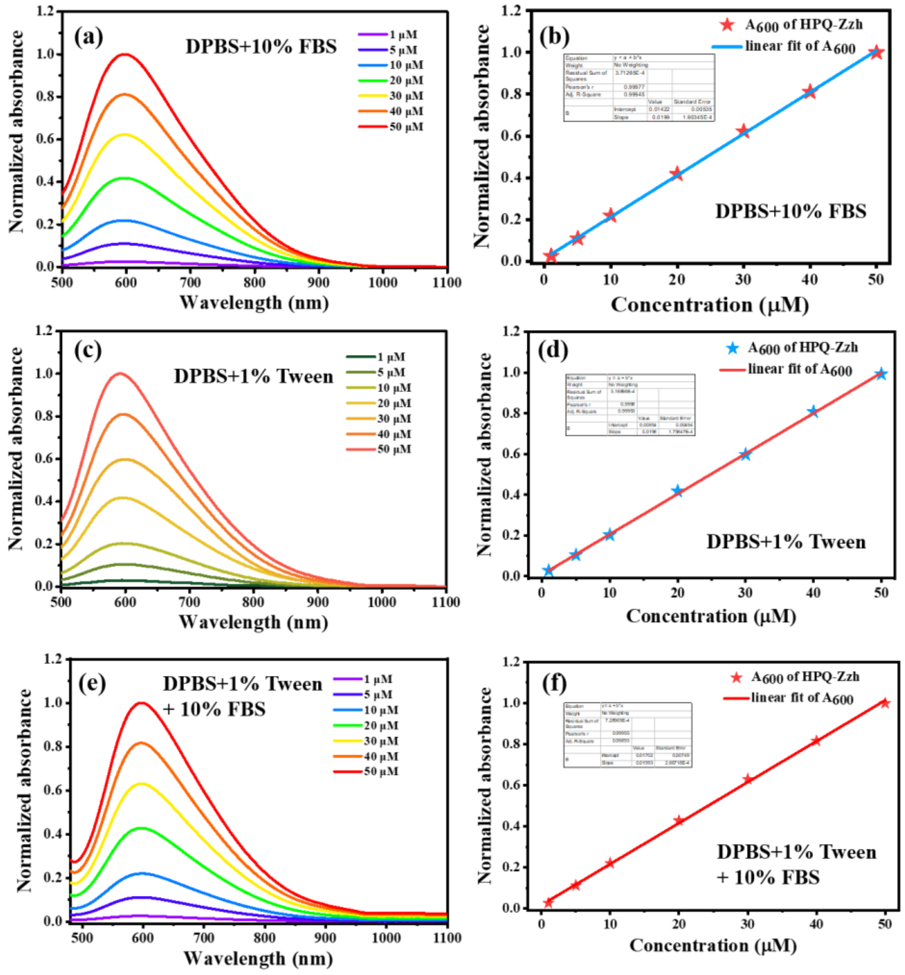

Supplementary Figure 14 | (a) Absorbance spectrum of **HPQ-Zzh** at different concentrations in DPBS

+10% FBS. **(b)** A_{600} linear relationship for a. **(c)** Absorbance spectrum of **HPQ-Zzh** at different concentrations in DPBS +1% Tween. **(d)** A_{600} linear relationship for c. **(e)** Absorbance spectrum of **HPQ-Zzh** at different concentrations in DPBS +1% Tween+10% FBS. **(f)** A_{600} linear relationship for e.

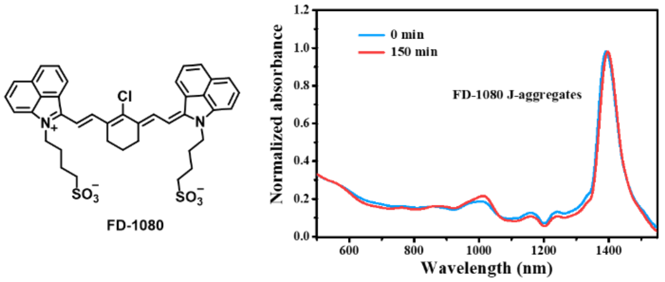

Supplementary Figure 15 | Structure of **FD-1080** and absorbance spectrum of **FD-1080** J-aggregates (in DMPC) at different time[2].

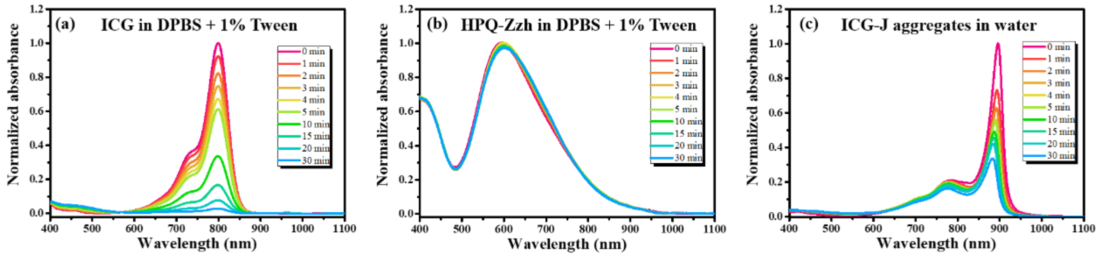

Supplementary Figure 16 | Normalized absorbance spectrum of **(a)** ICG (in DPBS + 1% Tween), **(b)** **HPQ-Zzh** (in DPBS + 1% Tween), and **(c)** ICG-J-aggregates (in water) under 200 mW cm^{-2} 808 nm laser at different time.

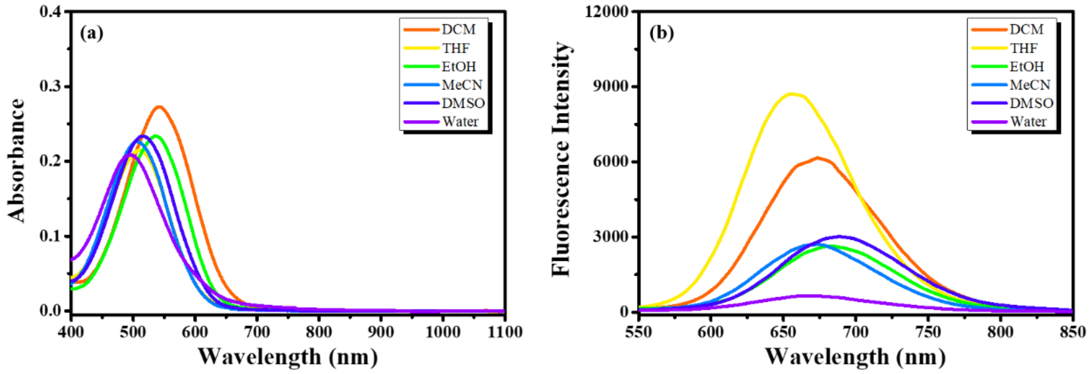

Supplementary Figure 17 | Absorbance **(a)** and Fluorescence **(b)** spectrum of **HP-Zzh** in different solvents, λ_{ex} = 500 nm.

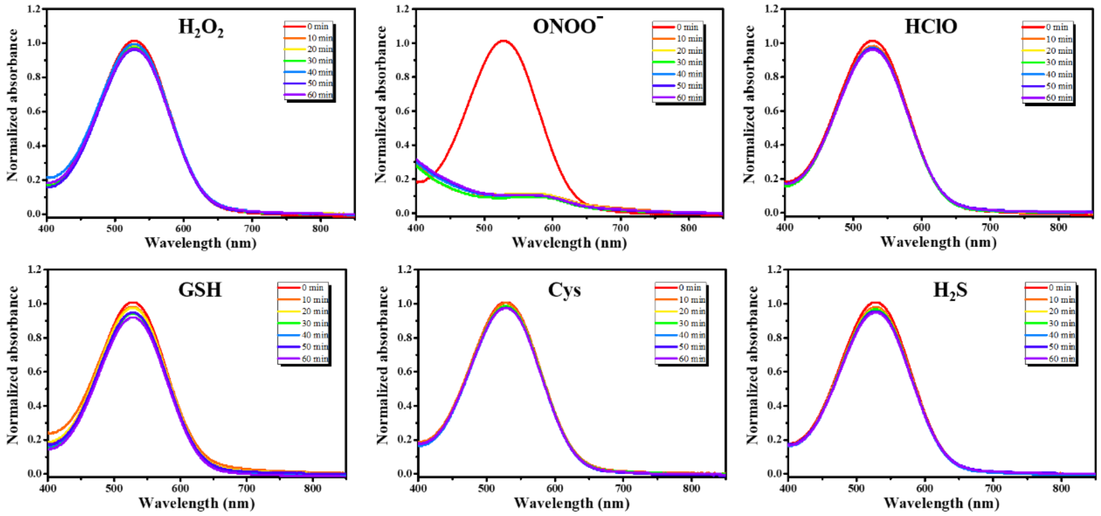

Supplementary Figure 18 | Normalized absorbance spectrum of **HP-Zzh** in DPBS +1% Tween in the presence of different active substances. H_2O_2 -100 μM, $ONOO^-$ - 20 μM, HClO -50 μM, GSH -1 mM, Cys -50 μM, H_2S -100 μM.

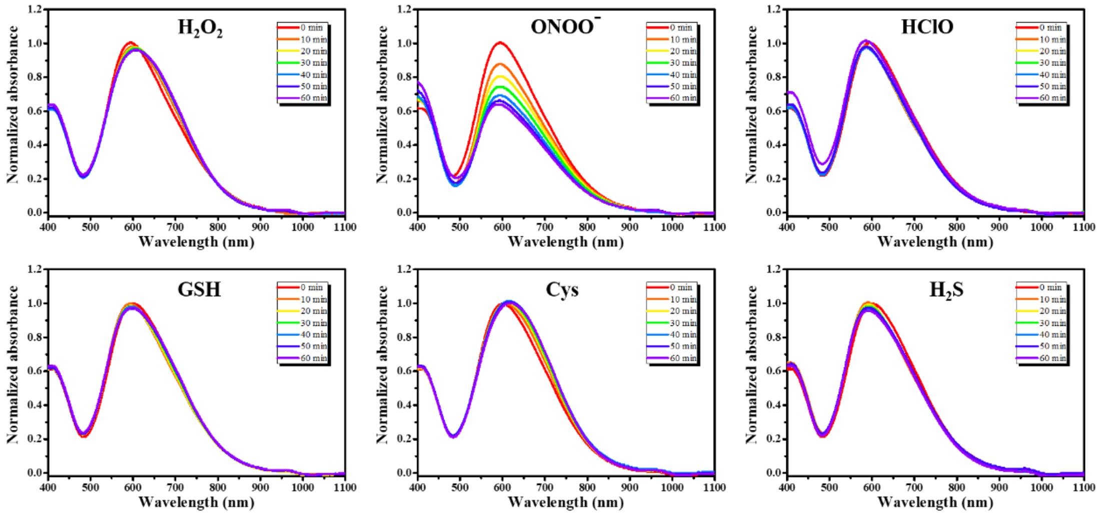

Supplementary Figure 19 | Normalized absorbance spectrum of **HPQ-Zzh** in DPBS +1% Tween in the presence of different active substances. H_2O_2 -100 μM, $ONOO^-$ - 20 μM, HClO -50 μM, GSH -1 mM, Cys -50 μM, H_2S -100 μM.

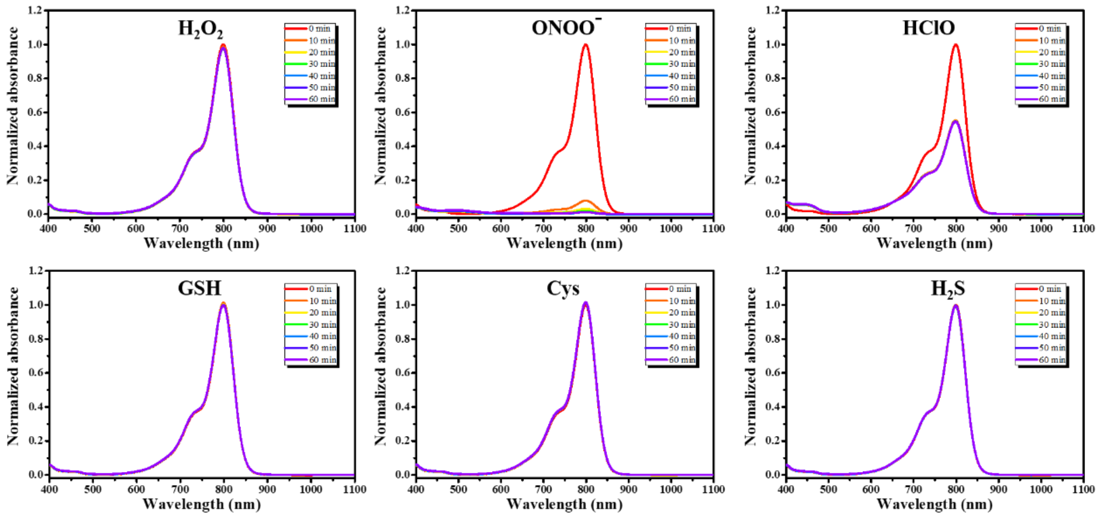

Supplementary Figure 20 | Normalized absorbance spectrum of ICG in DPBS +1% Tween in the presence of different active substances. H_2O_2 -100 μM, $ONOO^-$ - 20 μM, HClO -50 μM, GSH -1 mM, Cys -50 μM, H_2S -100 μM.

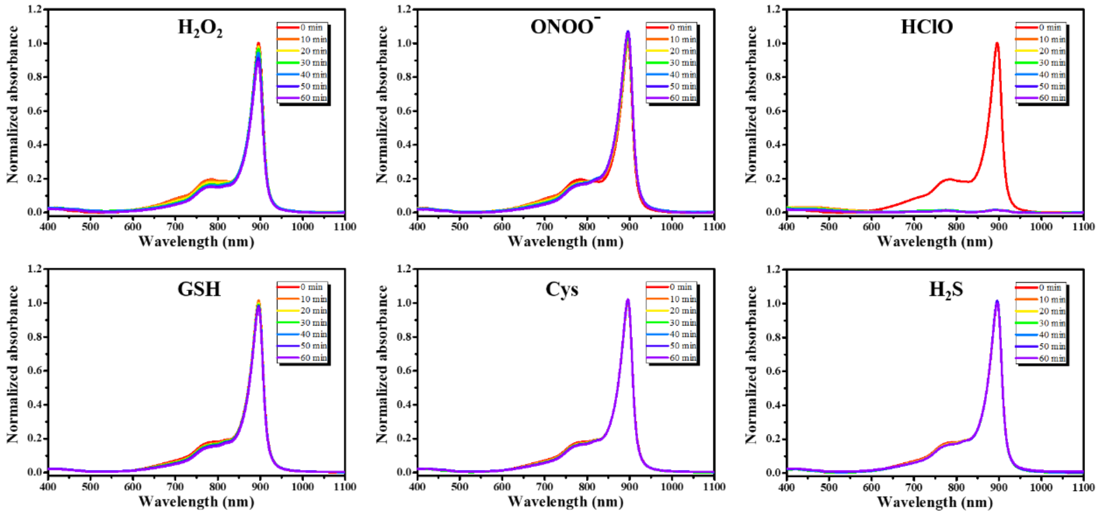

Supplementary Figure 21 | Normalized absorbance spectrum of ICG-J-aggregates in water in the presence of different active substances. H_2O_2 -100 μM, $ONOO^-$ - 20 μM, HClO -50 μM, GSH -1 mM, Cys -50 μM, H_2S -100 μM.

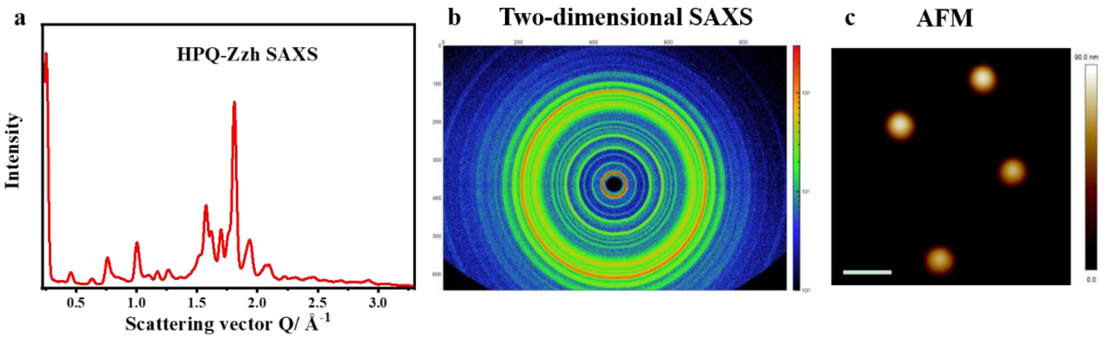

Supplementary Figure 22 | (a) One-dimensional SAXS atlas, **(b)** Two-dimensional SAXS pattern of **HPQ-Zzh**, and **(c)** AFM results of **HPQ-Zzh**, scale bar: 1 μm. The experiment was repeated three times independently, with similar results.

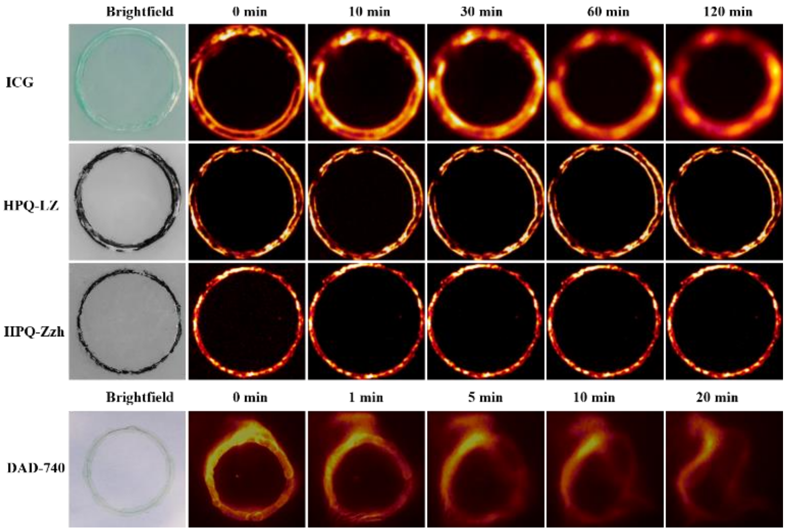

Supplementary Figure 23 | Diffusion situations of ICG, **DAD-740**, **HPQ-LZ** and **HPQ-Zzh** (vs. time). λ_{ex} = 808 nm, Collection channel: 1000-1700 nm. Our NIR-II-J-aggregates have good anti-diffusion ability.

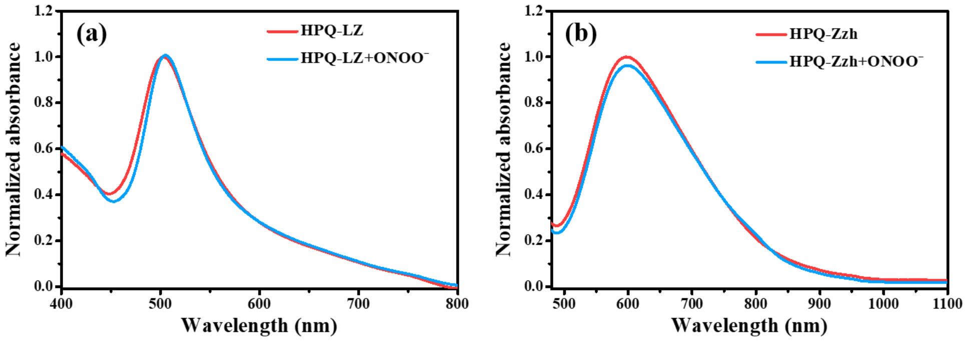

Supplementary Figure 24 | Normalized absorbance of **HPQ-LZ** (a) and **HPQ-Zzh** (b) in the absence and presence of $ONOO^-$ in DPBS +1% Tween.

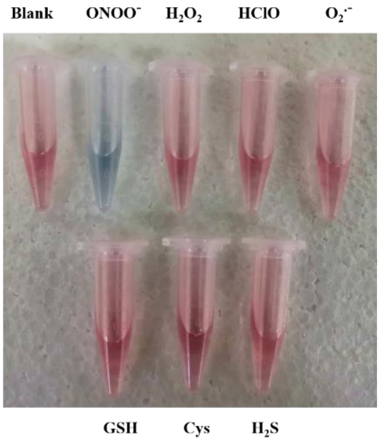

Supplementary Figure 25 | Photos of **HPQ-Zzh-B** (20 μM) to various analytes (1. blank, 2. 5 μM $ONOO^-$, 3. 100 μM H_2O_2, 4. 50 μM HOCl, 5. 20 μM $O_2^{\cdot -}$, 6. 3 mM GSH, 7. 100 μM Cys, 8. 100 μM H_2S.).

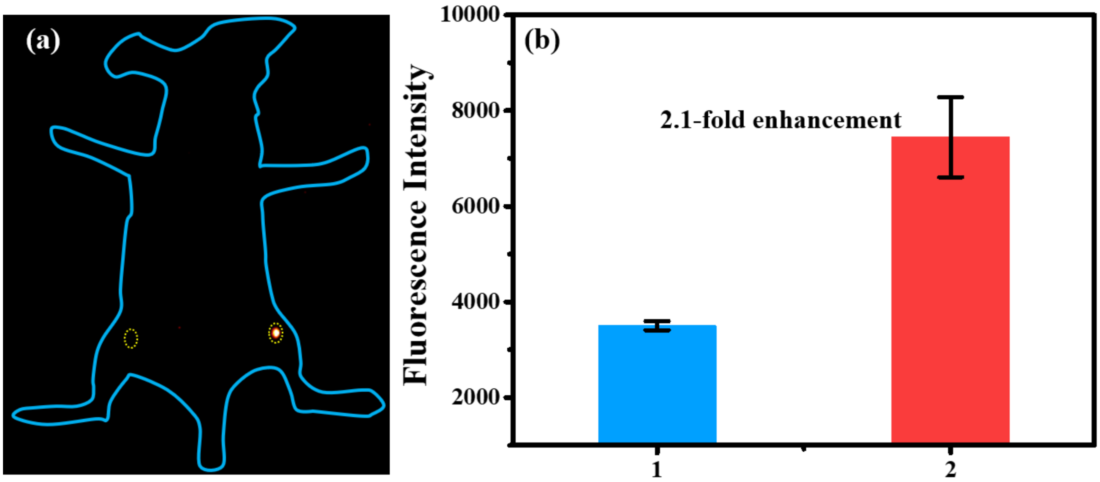

Supplementary Figure 26 | (a) NIR-II fluorescence of **HPQ-Zzh-B** (Left, 1) and **HPQ-Zzh-B** with $ONOO^-$ (Right, 2) in vivo, respectively, λ_{ex} = 808 nm, Collection channel: 1000-1700 nm. **(b)** Histogram for a. **HPQ-Zzh-B**: 100 μM, 25 μL, $ONOO^-$: 400 μM, 10 μL. Data were presented as mean ± s.d. derived from n = 3 independent biological samples.

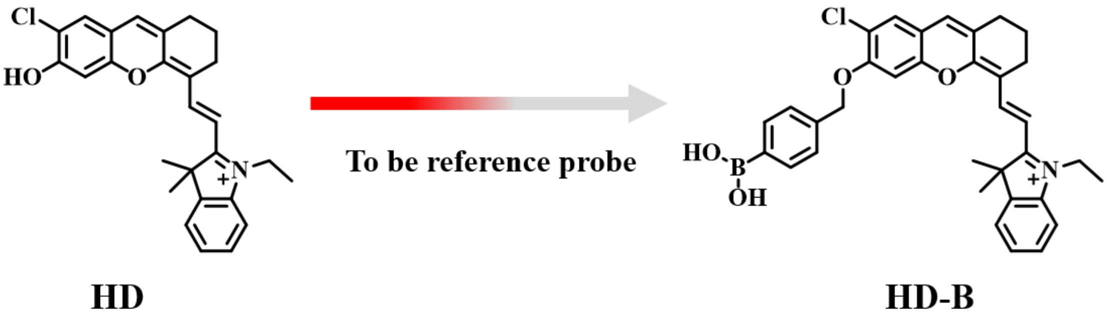

Supplementary Figure 27 | HD dye and construction of the reference probe **HD-B**.

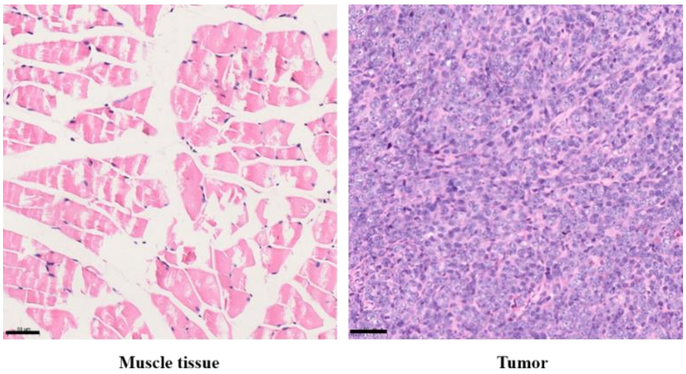

Supplementary Figure 28 | Histology studies of 4T1 tumor-bearing mice for muscle tissue and subcutaneous tumor, scale bar: 50 μm. The experiment was repeated three times independently, with similar results. Histological examination was according to a conventional method, and stained with hematoxylin and eosin (H&E).

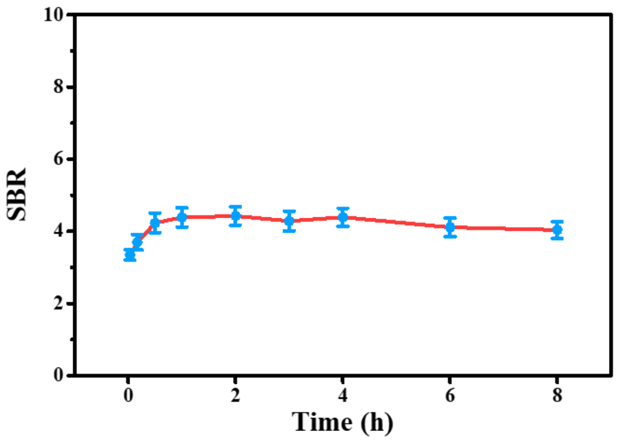

Supplementary Figure 29 | Signal-to-background ratio (SBR) vs. time (h) after injecting **HPQ-Zzh-B** at tumor. SBR remained stable for 1-8 h after probe injection. Data were presented as mean ± s.d. derived from n = 3 independent biological samples.

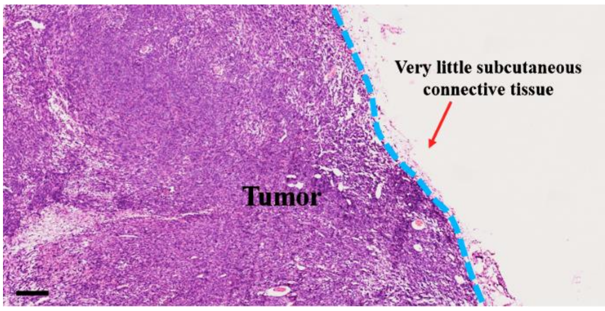

Supplementary Figure 30 | Histology studies of tumor resected after NIR-II fluorescence-mediated surgery for subcutaneous tumor, scale bar: 200 μm. The experiment was repeated three times independently, with similar results. After surgical navigation, the subcutaneous tumor had only a small amount of subcutaneous connective tissue. The histological studies indicated that NIR-II fluorescence based on **HPQ-Zzh-B**, could effectively distinguish the boundary between normal and tumor tissues.

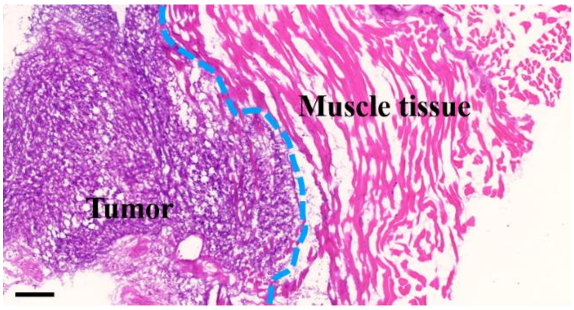

Supplementary Figure 31 | Histology studies of tumor resected after NIR-I fluorescence-mediated surgery for subcutaneous tumor, scale bar: 200 μm. The experiment was repeated three times independently, with similar results. After surgical navigation, the resected tissue contained not only a large amount of tumor tissue, but also a large amount of muscle tissue. The histological studies indicated that NIR-I fluorescence based on **HD-B**, could not effectively distinguish the boundary between normal and tumor tissues.

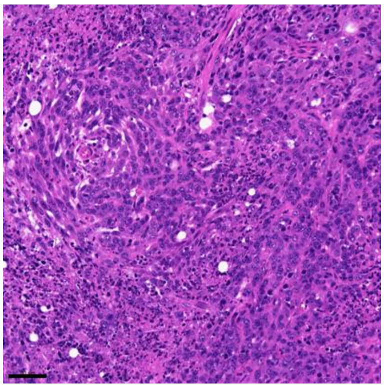

Supplementary Figure 32 | Histology studies of 4T1 tumor-bearing mice for primary tumor, scale bar: 50 μm. The experiment was repeated three times independently, with similar results. Histological examination was according to a conventional method, and stained with hematoxylin and eosin (H&E).

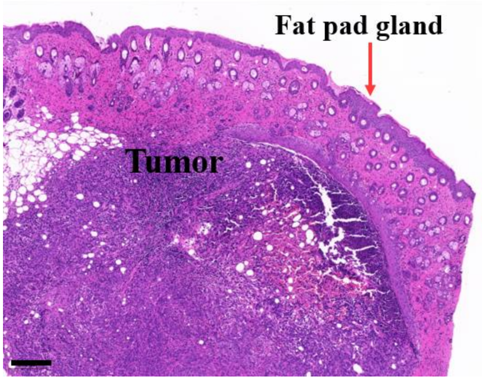

Supplementary Figure 33 | Histology studies of tumor resected after NIR-II fluorescence-mediated surgery for primary tumor, scale bar: 200 μm. The experiment was repeated three times independently, with similar results. After surgical navigation, the primary tumor had only a small amount of fat pad gland. The histological studies indicated that NIR-II fluorescence based on **HPQ-Zzh-B**, could effectively distinguish the boundary between normal and tumor tissues.

3. Synthesis of compounds

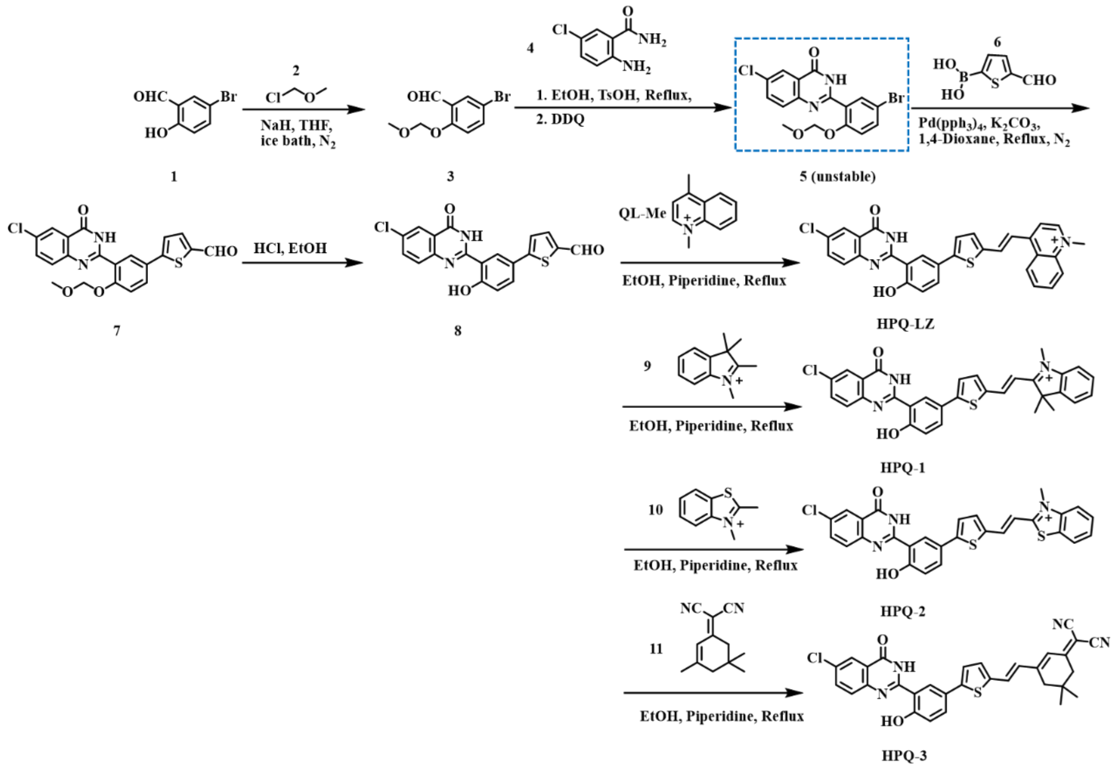

Synthesis of compound 8. Compound 1 (2.0 g, 10 mmol, 98% purity) and NaH (0.8 g, 20 mmol, 60% purity) were placed in a two-necked round bottom flask and anhydrous THF (20 mL) was added through the needle and stirred at R.T. for 1 h under nitrogen. After that, 1.61 g compound 2 (1.61 g, 20 mmol, 98% purity) dripped slowly and stirred for 1 h. The reaction solution was dried by rotary evaporator and slowly added organic phase. After extraction, the crude product is directly used in the next reaction. Such crude product and compound 4 (1.0 equiv., 98% purity) was dissolved in absolute EtOH to give a solution at room temperature. This reaction mixture was heated to reflux for 30 min, and then p-TsOH monohydrate (0.02 equiv.) was added, and reflux was continued for 2 h. The suspension was cooled to room temperature, and then 2,3-dichloro-5,6-dicyano-1,4-benzoquinone (DDQ, 1.01 equiv., 98% purity) was added in several portions, and the reaction mixture was further stirred overnight at room temperature. The precipitate was filtered, washed three times with absolute EtOH, and then twice with diethyl ether to get white solid which is easy to decompose. Immediately, this white solid, Compound 6 (1.50 g, 7.6 mmol, 98% purity), K_2CO_3 (2.00 g, 14.5 mmol), $Pd(PPh_3)_4$ (0.58 g, 0.506 mmol, 98% purity) were placed in a round bottom flask and dioxane (20 mL) was added and reflux for 24 h under N_2. After extraction, the crude product is directly hydrolyzed in an aqueous solution of hydrochloric acid and ethanol to obtain compound 8, yellow solid 1.0 g (Yield 25.1%). ^1H NMR (400 MHz, DMSO-d_6) δ 9.93 (s, 1H), 8.79 – 8.59 (m, 1H), 8.23 – 8.04 (m, 2H), 7.97 – 7.77 (m, 4H), 7.14 (d, J = 8.8 Hz, 1H). ^{13}C NMR (101 MHz, DMSO-d_6) δ 184.47, 171.39, 164.31, 155.89, 154.30, 153.47, 150.75, 141.49, 140.12, 134.38, 131.95, 129.34, 128.44, 127.68, 126.95, 125.11, 122.51, 117.26, 95.43. MS (MALDI-TOF): calcd for $C_{19}H_{12}ClN_2O_3S$ $(M+H)^+$ 383.03, found 382.98.

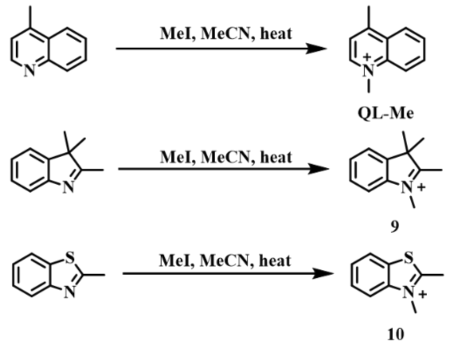

Synthesis of compound QL-Me. 4-methylquinoline (143 mg, 1.0 mmol, 1 Equiv., 98% purity) reacted with RI (3 Equiv.) in MeCN and refluxed at 81 °C for 12 h under nitrogen. The precipitate was filtered to give a white solid 271 mg (Yield 95.1%). ^{1}H NMR (400 MHz, DMSO-*d*$_6$) δ 9.43 (d, *J* = 6.0 Hz, 1H), 8.52 (m, 2H), 8.32 – 8.23 (m, 1H), 8.07 (q, *J* = 6.7, 5.1 Hz, 2H), 4.60 (s, 3H), 3.01 (s, 3H). ^{13}C NMR (101 MHz, DMSO-*d*$_6$) δ 158.60, 149.46, 138.13, 130.11, 128.92, 127.26, 122.93, 120.03, 45.53, 20.12. MS (MALDI-TOF): calcd for $C_{11}H_{12}N^+$ 158.10, found 158.06.

Synthesis of compound 9. 2,3,3-Trimethylindolenine (159 mg, 1.0 mmol, 1 Equiv., 98% purity) reacted with RI (3 Equiv.) in MeCN and refluxed at 81 °C for 12 h under nitrogen. The precipitate was filtered to give a purple solid 290 mg (Yield 96.3%). ^{1}H NMR (400 MHz, DMSO-*d*$_6$) δ 7.99 – 7.91 (m, 1H), 7.88 – 7.83 (m, 1H), 7.67 – 7.55 (m, 2H), 4.00 (s, 3H), 2.82 (s, 3H), 1.54 (s, 6H). ^{13}C NMR (101 MHz, DMSO-*d*$_6$) δ 196.45, 142.57, 142.07, 129.73, 129.26, 123.82, 115.65, 54.43, 35.50, 22.22, 15.08. MS (MALDI-TOF): calcd for $C_{12}H_{16}N^+$ M^+ 174.13, found 174.12.

Synthesis of compound 10. 2-Methylbenzothiazole (149 mg, 1.0 mmol, 1 Equiv., 98% purity) reacted with RI (3 Equiv.) in MeCN and refluxed at 81 °C for 12 h under nitrogen. The precipitate was filtered to give a white solid 273 mg (Yield 93.8%). ^{1}H NMR (400 MHz, DMSO-*d*$_6$) δ 8.50 (d, *J* = 8.1 Hz, 1H), 8.30 (d, *J* = 8.4 Hz, 1H), 7.86 (t, *J* = 7.8 Hz, 1H), 7.77 (t, *J* = 7.7 Hz, 1H), 4.23 (s, 3H), 3.23 (s, 3H). ^{13}C NMR (101 MHz, DMSO-*d*$_6$) δ 177.50, 141.97, 129.66, 129.09, 128.42, 125.04, 117.25, 37.21, 18.29. MS (MALDI-TOF): calcd for $C_9H_{10}NS^+$ M^+ 164.05, found 163.96.

Synthesis of compound HPQ-LZ. Compound 8 (50.0 mg, 0.13 mmol) and QL-Me (37.0 mg, 0.13 mmol) were placed in a round bottom flask, and 10 mL ethanol and a drop of piperidine were added and refluxed at 81 °C for 6 h under nitrogen. After cooling, the reaction solution was dried by rotary evaporator and purified by column chromatography (silica gel, DCM/MeOH = 10:1) to give a black solid 30 mg (Yield 35.6%). ^{1}H NMR (400 MHz, DMSO-*d*$_6$) δ 9.22 (d, *J* = 9.2 Hz, 1H), 9.03 (d, *J* = 8.7 Hz, 1H), 8.67 (d, *J* = 2.5 Hz, 1H), 8.48 – 8.37 (m, 3H), 8.32 – 8.22 (m, 1H), 8.10 – 7.98 (m, 2H), 7.96 – 7.80 (m, 1H), 7.78 – 7.58 (m, 4H), 7.49 (d, *J* =4.0 Hz 1H), 6.72 (d, *J* = 8.0 Hz, 1H), 4.51 (s, 3H). ^{13}C NMR (101 MHz, DMSO-*d*$_6$) δ 186.35, 176.06, 174.58, 170.78, 168.11, 165.57, 162.15, 160.21, 159.07, 155.76, 152.61, 149.44, 147.79, 140.58, 139.22, 138.16, 135.39, 130.11, 129.48, 129.45, 127.26, 126.26, 122.93, 119.98, 116.60, 114.47, 113.05, 110.63, 109.25, 45.44. MS (MALDI-TOF): calcd for $C_{30}H_{21}ClN_3O_2S^+$ M^+ 522.10, found 521.87.

Synthesis of compound HPQ-1. Compound 8 (50.0 mg, 0.13 mmol) and compound 9 (39.0 mg, 0.13 mmol) were placed in a round bottom flask, and 10 mL ethanol and a drop of piperidine were added and

refluxed at 81 °C for 6 h under nitrogen. After cooling, the reaction solution was dried by rotary evaporator and purified by column chromatography (silica gel, DCM/MeOH = 10:1) to give a black solid 28 mg (Yield 42.1%). ^{1}H NMR (400 MHz, DMSO-d_6) δ 9.92 (s, 1H), 8.67 (s, 2H), 8.43 (s, 1H), 8.21 – 8.01 (m, 3H), 7.91 (s, 2H), 7.84 (d, J = 5.6 Hz, 2H), 7.78 (s, 1H), 7.58 (s, 1H), 7.09 (d, J = 6.9 Hz, 1H), 6.97 (d, J = 9.2 Hz, 1H), 4.07 (s, 3H), 1.81 (s, 6H). ^{13}C NMR (101 MHz, DMSO-d_6) δ 197.34, 184.22, 178.07, 170.28, 168.02, 165.55, 162.78, 160.19, 156.22, 154.47, 149.33, 145.04, 143.55, 139.74, 136.27, 135.14, 130.26, 128.83, 126.08, 125.46, 122.70, 120.68, 117.14, 114.80, 112.59, 105.10, 99.99, 61.06, 26.18, 16.23. MS (MALDI-TOF): calcd for $C_{31}H_{25}ClN_3O_2S^+$ M^+ 538.14, found 537.96.

Synthesis of compound HPQ-2. Compound 8 (50.0 mg, 0.13 mmol) and compound 10 (37.8 mg, 0.13 mmol) were placed in a round bottom flask, and 10 mL ethanol and a drop of piperidine were added and refluxed at 81 °C for 6 h under nitrogen. After cooling, the reaction solution was dried by rotary evaporator and purified by column chromatography (silica gel, DCM/MeOH = 10:1) to give a black solid 25 mg (Yield 29.3%). ^{1}H NMR (400 MHz, DMSO-d_6) δ 9.90 (s, 1H), 8.67 (s, 2H), 8.40 (s, 1H), 8.19 (d, J = 8.7 Hz, 1H), 8.05 (s, 1H), 7.93 (d, J = 3.9 Hz, 1H), 7.83 (s, 2H), 7.74 (d, J = 8.7 Hz, 2H), 7.66 (d, J = 2.7 Hz, 1H), 7.65 – 7.45 (m, 1H), 7.01 (d, J = 8.5 Hz, 1H), 6.88 (d, J = 9.7 Hz, 1H), 4.31 (s, 3H). ^{13}C NMR (101 MHz, DMSO-d_6) δ 200.83, 188.84, 185.27, 176.32, 172.63, 171.55, 169.69, 166.68, 162.33, 158.48, 155.78, 152.41, 149.86, 146.28, 141.27, 139.82, 138.43, 136.96, 136.07, 135.16, 131.79, 130.68, 128.83, 120.11, 118.81, 112.46, 110.48, 25.71. MS (MALDI-TOF): calcd for $C_{28}H_{19}ClN_3O_2S_2^+$ M^+ 528.06, found 527.91.

Synthesis of compound HPQ-3. Compound 8 (50.0 mg, 0.13 mmol) and compound 11 (25.0 mg, 0.13 mmol) were placed in a round bottom flask, and 10 mL ethanol and a drop of piperidine were added and refluxed at 81 °C for 6 h under nitrogen. After cooling, the reaction solution was dried by rotary evaporator and purified by column chromatography (silica gel, DCM/MeOH = 10:1) to give a red solid 10 mg (Yield 14.0%). ^{1}H NMR (400 MHz, DMSO-d_6) δ 9.92 (s, 1H), 8.65 (s, 1H), 8.42 (s, 1H), 8.19 – 7.99 (m, 3H), 7.90 (s, 3H), 7.12 (d, J = 9.0 Hz, 1H), 7.00 (d, J = 9.3 Hz, 1H), 2.75 – 2.55 (m, 2H), 2.43 (s, 2H), 1.04 (s, 6H). ^{13}C NMR (101 MHz, DMSO-d_6) δ 183.60, 177.66, 170.43, 167.52, 164.56, 160.60, 158.87, 154.14, 148.71, 146.49, 145.18, 143.16, 139.66, 135.54, 131.89, 130.73, 125.54, 122.65, 120.52, 119.53, 116.87, 114.58, 112.57, 109.63, 82.77, 27.93, 16.43, 6.10, 0.57. MS (MALDI-TOF): calcd for $C_{31}H_{23}ClN_4O_2S$ M^+ 550.12, found 550.88.

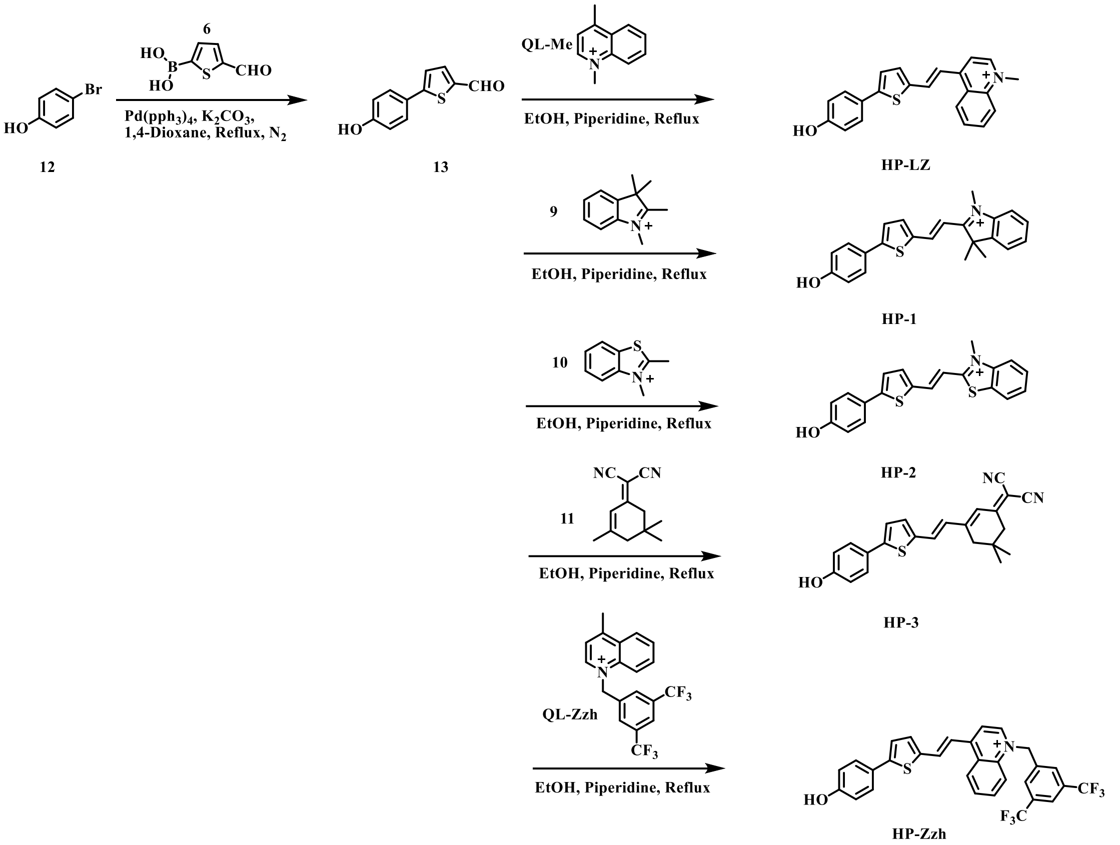

Synthesis of compound 13. Compound 12 (0.88 g, 5.06 mmol, 98% purity), Compound 6 (1.50 g, 7.6 mmol, 98% purity), K_2CO_3 (2.00 g, 14.5 mmol), $Pd(PPh_3)_4$ (0.58 g, 0.506 mmol, 98% purity) were placed in a round bottom flask and dioxane 20 mL was added and reflux for 24 h under N_2. After extraction, the reaction solution was dried by rotary evaporator and purified by column chromatography (silica gel, DCM/EtOH = 100:1) to give an orange solid 301 mg (Yield 29.1%). ^{1}H NMR (400 MHz, DMSO-d_6) δ 10.14 (s, 1H), 9.85 (s, 1H), 7.99 (d, J = 3.8 Hz, 1H), 7.65 (s, 2H), 7.56 (s, 1H), 6.88 (d, J = 8.0 Hz, 2H). ^{13}C NMR (101 MHz, DMSO-d_6) δ 184.14, 159.56, 154.22, 140.84, 140.09, 132.56, 131.90, 129.29, 128.27, 123.83, 116.58. MS (MALDI-TOF): calcd for $C_{11}H_8O_2S$ M^+ 204.02, found 204.91.

Synthesis of compound HP-LZ. Compound 13 (50.0 mg, 0.245 mmol) and QL-Me (70.0 mg, 0.245 mmol) were placed in a round bottom flask, and 10 mL ethanol and a drop of piperidine were added and refluxed at 81 °C for 6 h under nitrogen. After cooling, the reaction solution was dried by rotary evaporator and purified by column chromatography (silica gel, DCM/EtOH = 30:1) to give a red solid 26 mg (Yield 22.5%). ^{1}H NMR (400 MHz, DMSO-d_6) δ 9.97 (s, 1H), 9.30 (d, J = 6.7 Hz, 1H), 8.98 (d, J = 7.1 Hz, 1H), 8.37 – 8.30 (m, 3H), 8.28 – 8.20 (m, 1H), 8.06 (t, J = 7.9 Hz, 1H), 8.05 – 7.85 (m, 1H), 7.76 (d, J = 3.3 Hz, 1H), 7.63 (d, J = 8.7 Hz, 2H), 7.62 – 7.44 (m, 1H), 6.90 (d, J = 8.3 Hz, 2H), 4.53 (s, 3H). ^{13}C NMR (101 MHz, DMSO-d_6) δ 158.93, 152.52, 149.68, 148.06, 139.22, 138.95, 136.45, 135.36, 134.69, 129.59, 127.76, 126.67, 126.36, 124.55, 124.21, 119.77, 117.61, 116.59, 115.79, 44.96. MS (MALDI-TOF): calcd for $C_{22}H_{18}NOS^+$ M^+ 344.11, found 344.06.

Synthesis of compound HP-1. Compound 13 (50.0 mg, 0.245 mmol) and Compound 9 (70.0 mg, 0.245 mmol) were placed in a round bottom flask, and 10 mL ethanol and a drop of piperidine were added and refluxed at 81 °C for 6 h under nitrogen. After cooling, the reaction solution was dried by rotary

evaporator and purified by column chromatography (silica gel, DCM/EtOH = 30:1) to give a red solid 15 mg (Yield 12.6%). ^{1}H NMR (400 MHz, DMSO-d_6) δ 10.18 (s, 1H), 8.77 – 8.57 (m, 1H), 8.18 (d, *J* = 4.2 Hz, 1H), 7.87 (d, *J* = 7.5 Hz, 2H), 7.72 (d, *J* = 7.3 Hz, 3H), 7.71 – 7.51 (m, 2H), 7.30 – 7.10 (m, 1H), 6.94 (d, *J* = 8.3 Hz, 2H), 4.08 (s, 3H), 1.80 (s, 6H). ^{13}C NMR (101 MHz, DMSO-d_6) δ 180.83, 159.93, 155.31, 145.95, 143.64, 142.36, 140.56, 138.39, 129.36, 129.17, 128.41, 125.32, 124.04, 123.25, 116.81, 115.04, 109.90, 52.09, 34.32, 26.06. MS (MALDI-TOF): calcd for $C_{23}H_{22}NOS^+$ M^+ 360.14, found 360.09.

Synthesis of compound HP-2. Compound 13 (50.0 mg, 0.245 mmol) and Compound 10 (70.0 mg, 0.245 mmol) were placed in a round bottom flask, and 10 mL ethanol and a drop of piperidine were added and refluxed at 81 °C for 6 h under nitrogen. After cooling, the reaction solution was dried by rotary evaporator and purified by column chromatography (silica gel, DCM/EtOH = 30:1) to give a red solid 1.9 mg (Yield 1.5%). ^{1}H NMR (400 MHz, DMSO-d_6) δ 10.05 (s, 1H), 7.80 (m, 2H), 7.65 (d, *J* = 7.8 Hz, 1H), 7.55 (s, 1H), 7.43 (d, *J* = 8.7 Hz, 1H), 7.34 (m, 2H), 7.10 (d, *J* = 8.8 Hz, 1H), 6.90 (d, *J* = 8.3 Hz, 1H), 6.81 (d, *J* = 8.9 Hz, 1H), 3.94 (s, 3H). ^{13}C NMR (101 MHz, DMSO-d_6) δ 151.28, 140.93, 135.20, 128.37, 127.66, 126.89, 125.43, 124.55, 123.14, 116.49, 116.27, 113.75, 55.40. MS (MALDI-TOF): calcd for $C_{20}H_{16}NOS_2^+$ M^+ 350.07, found 350.04.

Synthesis of compound HP-3. Compound 13 (50.0 mg, 0.245 mmol) and Compound 11 (50.0 mg, 0.245 mmol) were placed in a round bottom flask, and 10 mL ethanol and a drop of piperidine were added and refluxed at 81 °C for 6 h under nitrogen. After cooling, the reaction solution was dried by rotary evaporator and purified by column chromatography (silica gel, DCM/EtOH = 30:1) to give a red solid 21 mg (Yield 23.0%). ^{1}H NMR (400 MHz, DMSO-d_6) δ 9.86 (s, 1H), 7.53 (d, *J* = 8.5 Hz, 2H), 7.50 – 7.40 (m, 1H), 7.38 (q, *J* = 3.7 Hz, 2H), 7.10 – 6.90 (m, 1H), 6.84 (d, *J* = 8.1 Hz, 3H), 2.50 (d, *J* = 5.3 Hz, 4H), 1.01 (s, 6H). ^{13}C NMR (101 MHz, DMSO-d_6) δ 170.30, 158.55, 156.11, 147.37, 139.76, 132.88, 131.55, 128.13, 127.49, 124.74, 123.78, 122.44, 116.49, 114.59, 113.71, 75.73, 42.67, 38.56, 32.09, 27.88. MS (MALDI-TOF): calcd for $C_{23}H_{20}N_2OS$ $(M+H)^+$ 372.13, found 373.83.

Synthesis of compound HP-Zzh. Compound 13 (50.0 mg, 0.245 mmol) and QL-Zzh (110.0 mg, 0.245 mmol) were placed in a round bottom flask, and 10 mL ethanol and a drop of piperidine were added and refluxed at 81 °C for 6 h under nitrogen. After cooling, the reaction solution was dried by rotary evaporator and purified by column chromatography (silica gel, DCM/EtOH = 30:1) to give a red solid 29 mg (Yield 18.6%). ^{1}H NMR (400 MHz, Methanol-d_4) δ 9.24 (s, 1H), 8.89 (s, 1H), 8.49 – 8.38 (m, 2H), 8.32 (d, *J* = 8.9 Hz, 1H), 8.18 (t, *J* = 7.9 Hz, 1H), 8.07 (s, 1H), 8.04 (d, *J* = 7.7 Hz, 1H), 8.00 (d, *J* = 5.8 Hz, 2H), 7.96 (s, 1H), 7.92 (s, 1H), 7.68 – 7.63 (m, 3H), 7.43 (d, *J* = 3.9 Hz, 1H), 6.89 (d, *J* = 8.6 Hz, 2H), 6.38 (s, 2H). ^{13}C NMR (101 MHz, CD_3OD_SPE) δ 151.51, 139.53, 138.91, 138.47, 137.81, 137.05, 135.49, 135.39, 132.19, 131.00, 129.44, 129.23, 128.46, 127.61, 127.27, 127.02, 126.46, 124.75, 123.27, 118.52, 116.29, 115.67, 115.24, 114.88, 58.21. MS (MALDI-TOF): calcd for $C_{30}H_{20}F_6NOS^+$ M^+ 556.12, found 556.16.

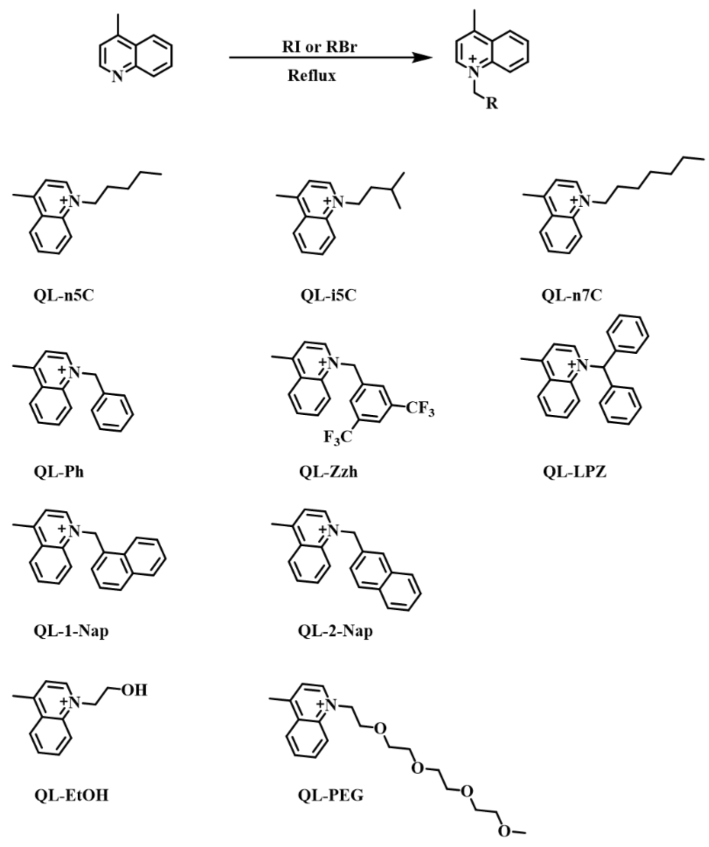

Synthesis of QL-R. 4-methylquinoline (1 Equiv., 98% purity) reacted with RX (X= Br or I) (3 Equiv.) in MeCN and refluxed at 81 °C for 12 h under nitrogen.

Synthesis of QL-n5C. 4-methylquinoline (143 mg, 1.0 mmol, 1 Equiv., 98% purity) reacted with RI (3 Equiv.) in MeCN and refluxed at 81 °C for 12 h under nitrogen. The precipitate was filtered to give a white solid 273 mg (Yield 80.1%). ^{1}H NMR (400 MHz, DMSO-d_6) δ 9.50 (d, J = 6.0 Hz, 1H), 8.62 (d, J = 8.9 Hz, 1H), 8.53 (d, J = 8.4 Hz, 1H), 8.25 (t, J = 7.9 Hz, 1H), 8.13 – 8.00 (m, 2H), 5.04 (t, J = 7.5 Hz, 2H), 3.00 (s, 3H), 1.94 (p, J = 7.6 Hz, 2H), 1.32 (m, 4H), 0.89 – 0.76 (m, 3H). ^{13}C NMR (101 MHz, DMSO-d_6) δ 158.94, 148.73, 137.09, 135.60, 130.06, 129.37, 127.64, 123.12, 119.87, 57.38, 55.55, 29.64, 28.32, 22.15, 20.37. MS (MALDI-TOF): calcd for $C_{15}H_{20}N^+$ M^+ 214.16, found 214.13.

Synthesis of QL-i5C. 4-methylquinoline (143 mg, 1.0 mmol, 1 Equiv., 98% purity) reacted with RI (3 Equiv.) in MeCN and refluxed at 81 °C for 12 h under nitrogen. The precipitate was filtered to give a white solid 269 mg (Yield 78.9%). ^{1}H NMR (400 MHz, DMSO-d_6) δ 9.53 (d, J = 6.1 Hz, 1H), 8.56 (d, J = 8.8 Hz, 2H), 8.28 (t, J = 8.6 Hz, 1H), 8.11 – 8.08 (m, 1H), 7.96 (s, 1H), 5.13 – 4.98 (m, 2H), 2.89 (s, 3H), 1.89 – 1.81 (m, 2H), 1.80 – 1.67 (m, 1H), 0.99 (d, J = 6.4 Hz, 6H). ^{13}C NMR (101 MHz, DMSO-d_6) δ 158.97, 148.76, 137.12, 135.61, 130.06, 129.40, 127.66, 123.13, 119.87, 57.39, 29.64, 28.33, 22.15, 20.34, 14.27. MS (MALDI-TOF): calcd for $C_{15}H_{20}N^+$ M^+ 214.16, found 214.16.

Synthesis of QL-n7C. 4-methylquinoline (143 mg, 1.0 mmol, 1 Equiv., 98% purity) reacted with RI (3 Equiv.) in MeCN and refluxed at 81 °C for 12 h under nitrogen. The precipitate was filtered to give a white solid 185 mg (Yield 50.1%). ^{1}H NMR (400 MHz, DMSO-d_6) δ 9.44 (d, J = 6.1 Hz, 1H), 8.70 – 8.50 (m, 2H), 8.29 (t, J = 8.0 Hz, 1H), 8.12 – 8.06 (m, 2H), 5.03 (t, J = 7.4 Hz, 2H), 3.03 (s, 3H), 2.01 –

1.92 (m, 2H), 1.40 – 1.24 (m, 8H), 0.87 (t, *J* = 6.7 Hz, 3H). ^{13}C NMR (101 MHz, DMSO-d_6) δ 159.03, 148.79, 137.21, 135.59, 130.06, 129.45, 127.64, 123.13, 119.81, 57.43, 31.52, 29.93, 28.59, 26.19, 22.41, 20.20, 14.37. MS (MALDI-TOF): calcd for $C_{17}H_{24}N^+$ M^+ 242.19, found 242.13.

Synthesis of QL-Ph. 4-methylquinoline (143 mg, 1.0 mmol, 1 Equiv., 98% purity) reacted with RBr (3 Equiv.) in MeCN and refluxed at 81 °C for 12 h under nitrogen. The precipitate was filtered to give a white solid 250 mg (Yield 79.9%). ^{1}H NMR (400 MHz, DMSO-d_6) δ 9.74 – 9.67 (m, 1H), 8.58 (d, *J* = 8.4 Hz, 1H), 8.51 (d, *J* = 9.0 Hz, 1H), 8.25 – 8.16 (m, 2H), 8.04 (t, *J* = 7.7 Hz, 1H), 7.40 (s, 5H), 6.38 (s, 2H), 3.07 (s, 3H). ^{13}C NMR (101 MHz, DMSO-d_6) δ 160.08, 149.65, 137.34, 135.64, 134.59, 130.13, 129.67, 129.53, 129.15, 127.77, 127.61, 123.44, 120.17, 59.96, 20.34. MS (MALDI-TOF): calcd for $C_{17}H_{16}N^+$ M^+ 234.13, found 234.09.

Synthesis of QL-Zzh. 4-methylquinoline (143 mg, 1.0 mmol, 1 Equiv., 98% purity) reacted with RBr (3 Equiv.) in MeCN and refluxed at 81 °C for 12 h under nitrogen. The precipitate was filtered to give a white solid 382 mg (Yield 84.9%). ^{1}H NMR (400 MHz, DMSO-d_6) δ 9.70 (d, *J* = 6.1 Hz, 1H), 8.61 (d, *J* = 8.3 Hz, 1H), 8.53 (d, *J* = 8.9 Hz, 1H), 8.24 (s, 4H), 8.18 (s, 1H), 8.06 (t, *J* = 7.7 Hz, 1H), 6.52 (s, 2H), 3.09 (s, 3H). ^{13}C NMR (101 MHz, DMSO-d_6) δ 160.45, 150.26, 137.52, 135.88, 131.40, 131.07, 130.17, 129.70, 129.31, 127.93, 124.85, 123.71, 122.14, 119.74, 58.73, 20.38. MS (MALDI-TOF): calcd for $C_{19}H_{14}F_6N^+$ M^+ 370.10, found 370.06.

Synthesis of QL-LPZ. 4-methylquinoline (143 mg, 1.0 mmol, 1 Equiv., 98% purity) reacted with RBr (3 Equiv.) in MeCN and refluxed at 81 °C for 12 h under nitrogen. The precipitate was filtered to give a white solid 273 mg (Yield 70.2%). ^{1}H NMR (400 MHz, DMSO-d_6) δ 9.29 (d, *J* = 5.5 Hz, 2H), 8.49 (d, *J* = 8.5 Hz, 2H), 8.33 (d, *J* = 8.5 Hz, 2H), 8.20 (t, *J* = 7.8 Hz, 2H), 8.07 – 8.01 (m, 4H), 7.51 (d, *J* = 6.4 Hz, 2H), 7.33 (d, *J* = 7.2 Hz, 2H), 3.07 (s, 1H), 3.02 (s, 3H). ^{13}C NMR (101 MHz, DMSO-d_6) δ 160.44, 158.62, 147.11, 144.66, 137.91, 137.22, 136.38, 135.75, 134.84, 130.00, 129.90, 129.45, 128.94, 128.29, 127.89, 127.03, 126.37, 123.47, 123.00, 121.44, 120.39, 70.85, 20.10. MS (MALDI-TOF): calcd for $C_{23}H_{20}N^+$ M^+ 310.16, found 310.12.

Synthesis of QL-1-Naph. 4-methylquinoline (143 mg, 1.0 mmol, 1 Equiv., 98% purity) reacted with RBr (3 Equiv.) in MeCN and refluxed at 81 °C for 12 h under nitrogen. The precipitate was filtered to give a white solid 180 mg (Yield 49.6%). ^{1}H NMR (400 MHz, DMSO-d_6) δ 9.47 (s, 1H), 8.63 (d, *J* = 8.4 Hz, 1H), 8.37 (d, *J* = 8.9 Hz, 1H), 8.25 (d, *J* = 8.1 Hz, 1H), 8.24 – 8.08 (m, 2H), 8.06 – 8.00 (m, 2H), 7.99 (d, *J* = 8.1 Hz, 1H), 7.81 – 7.61 (m, 2H), 7.38 (t, *J* = 7.7 Hz, 1H), 6.89 (s, 2H), 6.72 (d, *J* = 7.1 Hz, 1H), 3.10 (s, 3H). ^{13}C NMR (101 MHz, DMSO-d_6) δ 160.35, 149.37, 137.96, 135.84, 133.76, 130.27, 130.23, 130.18, 129.63, 129.53, 129.33, 127.79, 127.72, 127.20, 126.03, 124.77, 123.65, 123.49, 120.12, 57.97, 20.48. MS (MALDI-TOF): calcd for $C_{21}H_{18}N^+$ M^+ 284.14, found 284.09.

Synthesis of QL-2-Naph. 4-methylquinoline (143 mg, 1.0 mmol, 1 Equiv., 98% purity) reacted with RBr (3 Equiv.) in MeCN and refluxed at 81 °C for 12 h under nitrogen. The precipitate was filtered to give a white solid 255 mg (Yield 70.2%). ^{1}H NMR (400 MHz, DMSO-d_6) δ 9.75 (d, *J* = 6.1 Hz, 1H), 8.56 (t, *J* = 8.0 Hz, 2H), 8.24 (d, *J* = 6.1 Hz, 1H), 8.22 – 8.12 (m, 1H), 8.03 – 7.94 (m, 2H), 7.92 (d, *J* = 4.7 Hz, 2H), 7.88 – 7.83 (m, 1H), 7.63 – 7.55 (m, 3H), 6.53 (s, 2H), 3.07 (s, 3H). ^{13}C NMR (101 MHz, DMSO-d_6) δ 160.09, 149.77, 137.42, 135.65, 133.13, 133.07, 132.17, 130.11, 129.69, 129.32, 128.32,

128.13, 127.78, 127.26, 127.24, 126.76, 125.13, 123.53, 120.20, 60.17, 20.38. MS (MALDI-TOF): calcd for $C_{21}H_{18}N^+$ M^+ 284.14, found 284.10.

Synthesis of QL-EtOH. 4-methylquinoline (143 mg, 1.0 mmol, 1 Equiv., 98% purity) reacted with RBr (3 Equiv.) in MeCN and refluxed at 81 °C for 12 h under nitrogen. The precipitate was filtered to give a white solid 133 mg (Yield 49.8%). ^{1}H NMR (400 MHz, DMSO-d_6) δ 9.32 (d, J = 6.0 Hz, 1H), 8.66 (d, J = 8.9 Hz, 1H), 8.54 (d, J = 8.4 Hz, 1H), 8.23 (t, J = 7.9 Hz, 1H), 8.11 – 8.03 (m, 2H), 5.14 (t, J = 4.7 Hz, 2H), 3.91 (t, J = 4.7 Hz, 2H), 3.01 (s, 3H). ^{13}C NMR (101 MHz, DMSO-d_6) δ 158.93, 149.58, 137.38, 135.28, 129.92, 129.31, 127.49, 122.69, 120.02, 59.38, 20.26. MS (MALDI-TOF): calcd for $C_{12}H_{14}NO^+$ M^+ 188.11, found 188.06.

Synthesis of QL-PEG. 4-methylquinoline (143 mg, 1.0 mmol, 1 Equiv., 98% purity) reacted with RBr (3 Equiv.) in MeCN and refluxed at 81 °C for 12 h under nitrogen. The precipitate was filtered to give a white solid 165 mg (Yield 40.0%). ^{1}H NMR (400 MHz, DMSO-d_6) δ 9.38 (d, J = 6.0 Hz, 1H), 8.68 (d, J = 8.9 Hz, 1H), 8.51 (d, J = 8.4 Hz, 1H), 8.25 – 8.18 (m, 1H), 8.10 (d, J = 6.1 Hz, 1H), 8.02 (t, J = 7.7 Hz, 1H), 5.33 – 5.26 (m, 2H), 3.97 – 3.94 (m, 2H), 3.51 – 3.46 (m, 4H), 3.43 – 3.40 (m, 2H), 3.36 – 3.34 (m, 4H), 3.31 – 3.26 (m, 2H), 3.16 (s, 3H), 2.99 (s, 3H). ^{13}C NMR (101 MHz, DMSO-d_6) δ 159.26, 149.65, 137.23, 135.44, 130.03, 129.18, 127.52, 122.69, 119.98, 71.67, 70.30, 70.13, 70.09, 69.96, 68.21, 60.57, 58.48, 56.87, 20.27. MS (MALDI-TOF): calcd for $C_{19}H_{28}NO_4^+$ M^+ 334.20, found 334.18.

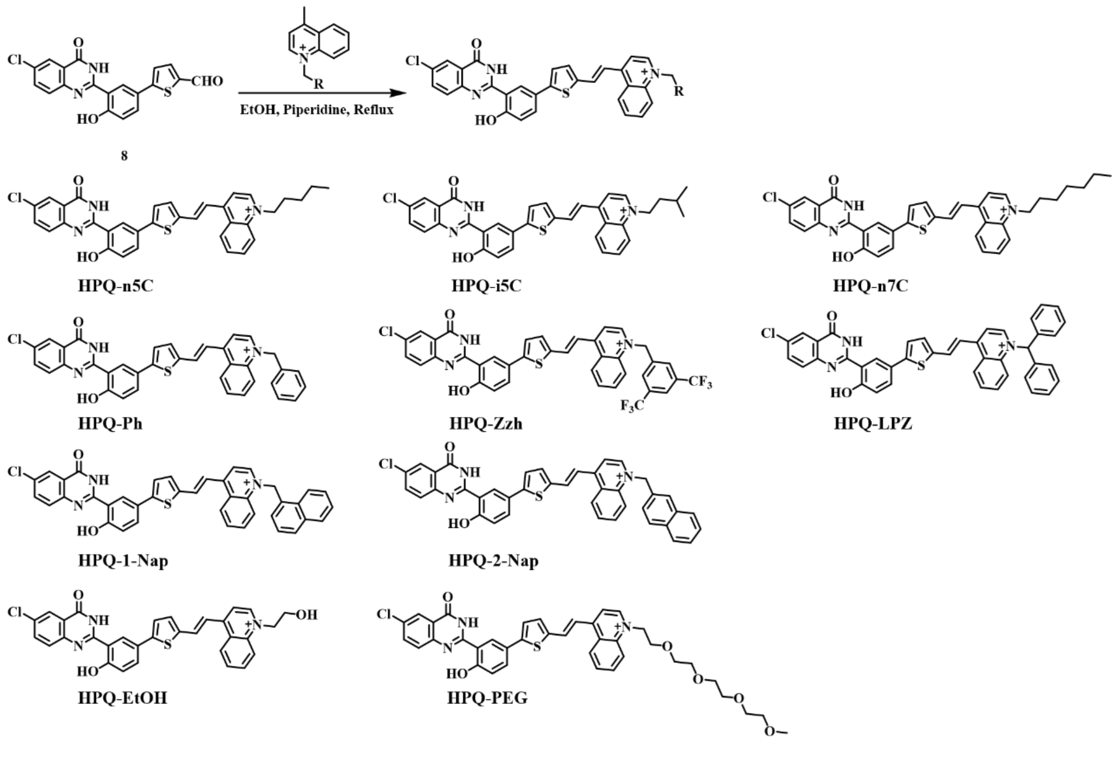

Synthesis of HPQ-R. Compound 8 (50.0 mg, 0.13 mmol) and QL-R (0.13 mmol) were placed in a round bottom flask, and 10 mL ethanol and a drop of piperidine were added and refluxed at 81 °C for 6 h under nitrogen. After cooling, the reaction solution was dried by rotary evaporator and purified by column chromatography.

Synthesis of HPQ-n5C. Compound 8 (50.0 mg, 0.13 mmol) and QL-n5C (0.13 mmol) were placed in a

round bottom flask, and 10 mL ethanol and a drop of piperidine were added and refluxed at 81 °C for 6 h under nitrogen. After cooling, the reaction solution was dried by rotary evaporator and purified by column chromatography (silica gel, DCM/MeOH = 10:1) to give a black solid 15 mg (Yield 16.3%). ^{1}H NMR (400 MHz, DMSO-d_6) δ 9.41 (s, 1H), 8.78 – 8.58 (m, 1H), 8.55 (d, *J* = 8.5 Hz, 2H), 8.49 – 8.39 (m, 1H), 8.35 – 8.24 (m, 2H), 8.22 – 7.89 (m, 4H), 7.87 – 7.62 (m, 2H), 7.60 – 7.31 (m, 1H), 6.99 – 6.70 (m, 1H), 5.01 (t, *J* = 7.4 Hz, 2H), 4.36 (s, 1H), 2.01 – 1.91 (m, 2H), 1.64 (d, *J* = 7.7 Hz, 2H), 1.36 (s, 2H), 0.91 – 0.85 (m, 3H). ^{13}C NMR (101 MHz, DMSO-d_6) δ 178.07, 161.01, 153.29, 152.78, 151.75, 147.08, 146.87, 140.13, 138.26, 137.15, 135.87, 135.39, 134.46, 133.60, 130.22, 129.31, 128.57, 127.06, 126.64, 126.36, 125.33, 122.77, 120.56, 119.44, 116.57, 115.41, 56.46, 29.52, 28.45, 22.19, 14.30. MS (MALDI-TOF): calcd for $C_{34}H_{29}ClN_3O_2S^+$ M^+ 578.17, found 578.09.

Synthesis of HPQ-i5C. Compound 8 (50.0 mg, 0.13 mmol) and QL-i5C (0.13 mmol) were placed in a round bottom flask, and 10 mL ethanol and a drop of piperidine were added and refluxed at 81 °C for 6 h under nitrogen. After cooling, the reaction solution was dried by rotary evaporator and purified by column chromatography (silica gel, DCM/MeOH = 10:1) to give a black solid 17 mg (Yield 18.5%). ^{1}H NMR (400 MHz, DMSO-d_6) δ 9.25 (d, *J* = 6.1 Hz, 1H), 9.14 (d, *J* = 6.2 Hz, 1H), 8.99 (d, *J* = 8.5 Hz, 1H), 8.65 – 8.61 (m, 1H), 8.54 (t, *J* = 9.6 Hz, 1H), 8.53 – 8.33 (m, 2H), 8.30 – 8.12 (m, 2H), 8.09 – 8.03 (m, 2H), 8.02 – 7.92 (m, 1H), 7.79 (d, *J* = 8.6 Hz, 1H), 7.75 (s, 1H), 7.58 (s, 1H), 6.87 (s, 1H), 5.16 – 5.03 (m, 2H), 1.68 – 1.63 (m, 6H), 1.56 (q, *J* = 5.6 Hz, 3H). MS (MALDI-TOF): calcd for $C_{34}H_{29}ClN_3O_2S^+$ M^+ 578.17, found 578.12. Due to the poor solubility of HPQ-i5C (250 μM) in DMSO, its ^{13}C NMR signals are very low even 10000 times scan (the concentrations of organic compounds for ^{13}C NMR test are typically greater than 10 mM).

Synthesis of HPQ-n7C. Compound 8 (50.0 mg, 0.13 mmol) and QL-n7C (0.13 mmol) were placed in a round bottom flask, and 10 mL ethanol and a drop of piperidine were added and refluxed at 81 °C for 6 h under nitrogen. After cooling, the reaction solution was dried by rotary evaporator and purified by column chromatography (silica gel, DCM/MeOH = 10:1) to give a black solid 11 mg (Yield 11.6%). ^{1}H NMR (400 MHz, DMSO-d_6) δ 9.41 (d, *J* = 6.0 Hz, 2H), 8.67 – 8.47 (m, 4H), 8.40 (s, 1H), 8.26 (t, *J* = 7.6 Hz, 2H), 8.07 (d, *J* = 9.6 Hz, 4H), 8.00 – 7.87 (m, 1H), 7.85 (d, *J* = 8.6 Hz, 1H), 6.99 (d, *J* = 8.7 Hz, 1H), 4.99 (d, *J* = 7.4 Hz, 2H), 2.04 – 1.84 (m, 4H), 1.72 – 1.60 (m, 2H), 1.32 (s, 4H), 0.84 (d, *J* = 7.0 Hz, 3H). ^{13}C NMR (101 MHz, DMSO-d_6) δ 193.76, 191.36, 171.47, 166.61, 164.26, 159.26, 159.05, 156.49, 148.81, 148.41, 139.62, 137.23, 136.61, 136.54, 135.60, 135.58, 131.83, 131.63, 130.74, 130.05, 129.46, 127.66, 123.13, 122.13, 120.52, 119.84, 119.80, 57.42, 44.20, 31.52, 29.92, 28.59, 26.19, 22.41, 20.16, 14.37. MS (MALDI-TOF): calcd for $C_{36}H_{33}ClN_3O_2S^+$ M^+ 606.20, found 606.23.

Synthesis of HPQ-Ph. Compound 8 (50.0 mg, 0.13 mmol) and QL-Ph (0.13 mmol) were placed in a round bottom flask, and 10 mL ethanol and a drop of piperidine were added and refluxed at 81 °C for 6 h under nitrogen. After cooling, the reaction solution was dried by rotary evaporator and purified by column chromatography (silica gel, DCM/MeOH = 10:1) to give a black solid 19 mg (Yield 21.6%). ^{1}H NMR (400 MHz, DMSO-d_6) δ 9.43 (d, *J* = 8.0 Hz, 1H), 9.08 – 9.00 (m, 1H), 8.67 (d, *J* = 2.3 Hz, 1H), 8.55 – 8.48 (m, 2H), 8.33 (d, *J* = 9.3 Hz, 1H), 8.15 (d, *J* = 8.3 Hz, 1H), 8.01 – 7.93 (m, 3H), 7.74 (d, *J* = 3.9 Hz, 1H), 7.73 – 7.53 (m, 3H), 7.51 (s, 1H), 7.48 – 7.28 (m, 6H), 6.21 (s, 2H). MS (MALDI-TOF): calcd for $C_{36}H_{25}ClN_3O_2S^+$ M^+ 598.14, found 598.18. Due to the poor solubility of HPQ-Ph (225 μM) in DMSO, its ^{13}C NMR signals are very low even 10000 times scan (the concentrations of organic

compounds for ^{13}C NMR test are typically greater than 10 mM).

Synthesis of HPQ-Zzh. Compound 8 (50.0 mg, 0.13 mmol) and QL-Zzh (0.13 mmol) were placed in a round bottom flask, and 10 mL ethanol and a drop of piperidine were added and refluxed at 81 °C for 6 h under nitrogen. After cooling, the reaction solution was dried by rotary evaporator and purified by column chromatography (silica gel, DCM/MeOH = 10:1) to give a black solid 30 mg (Yield 28.6%). ^{1}H NMR (400 MHz, DMSO-d_6) δ 9.49 – 9.42 (m, 1H), 9.08 (d, *J* = 9.1 Hz, 1H), 8.69 (d, *J* = 2.6 Hz, 1H), 8.60 – 8.50 (m, 2H), 8.36 (d, *J* = 9.3 Hz, 1H), 8.19 (d, *J* = 6.3 Hz, 4H), 8.03 – 7.96 (m, 3H), 7.77 (d, *J* = 3.9 Hz, 1H), 7.72 – 7.65 (m, 3H), 7.53 (d, *J* = 4.0 Hz, 1H), 6.73 (d, *J* = 9.6 Hz, 1H), 6.35 (s, 2H). MS (MALDI-TOF): calcd for $C_{38}H_{23}ClF_6N_3O_2S^+$ M^+ 734.11, found 734.02. Due to the poor solubility of HPQ-Zzh (200 μM) in DMSO, its ^{13}C NMR signals are very low even 10000 times scan (the concentrations of organic compounds for ^{13}C NMR test are typically greater than 10 mM).

Synthesis of HPQ-LPZ. Compound 8 (50.0 mg, 0.13 mmol) and QL-LPZ (0.13 mmol) were placed in a round bottom flask, and 10 mL ethanol and a drop of piperidine were added and refluxed at 81 °C for 6 h under nitrogen. After cooling, the reaction solution was dried by rotary evaporator and purified by column chromatography (silica gel, DCM/MeOH = 10:1) to give a black solid 14 mg (Yield 14.3%). ^{1}H NMR (400 MHz, DMSO-d_6) δ 9.98 – 9.88 (m, 1H), 9.04 (d, *J* = 8.0 Hz, 1H), 8.67 – 8.53 (m, 3H), 8.42 – 8.24 (m, 3H), 8.22 – 8.14 (m, 1H), 8.06 – 7.95 (m, 4H), 7.93 – 7.85 (m, 1H), 7.84 – 7.65 (m, 5H), 7.60 –7.41 (m, 5H), 7.36 – 7.31 (m, 3H), 6.83 (d, *J* = 8.4 Hz, 1H), 5.32 (s, 1H). ^{13}C NMR (101 MHz, DMSO-d_6) δ 184.39, 183.83, 162.63, 160.84, 156.16, 153.65, 152.06, 144.84, 142.15, 139.98, 139.79, 139.12, 137.80, 136.73, 135.62, 135.11, 134.14, 131.56, 131.24, 130.66, 129.91, 129.42, 128.83, 128.67, 127.28, 126.96, 126.43, 125.37, 122.76, 116.94, 116.36, 115.59, 95.45, 70.23. MS (ESI): calcd for $C_{42}H_{29}ClN_3O_2S^+$ M^+ 674.17, found 674.00.

Synthesis of HPQ-1-Naph. Compound 8 (50.0 mg, 0.13 mmol) and QL-1-Naph (0.13 mmol) were placed in a round bottom flask, and 10 mL ethanol and a drop of piperidine were added and refluxed at 81 °C for 6 h under nitrogen. After cooling, the reaction solution was dried by rotary evaporator and purified by column chromatography (silica gel, DCM/MeOH = 10:1) to give a black solid 11 mg (Yield 11.6%). ^{1}H NMR (400 MHz, DMSO-d_6) δ 9.25 (d, *J* = 6.1 Hz, 1H), 9.08 (d, *J* = 8.4 Hz, 1H), 8.68 (d, *J* = 2.6 Hz, 1H), 8.55 – 8.45 (m, 2H), 8.25 (d, *J* = 8.6 Hz, 1H), 8.17 (d, *J* = 8.8 Hz, 1H), 8.11 – 8.06 (m, 2H), 8.01 – 7.99 (m, 1H), 7.97 (d, *J* = 8.1 Hz, 2H), 7.93 (s, 1H), 7.78 – 7.73 (m, 2H), 7.71 (s, 1H), 7.69 (d, *J* = 2.6 Hz, 1H), 7.65 (d, *J* = 8.8 Hz, 2H), 7.61 (d, *J* = 2.3 Hz, 1H), 7.58 (s, 1H), 7.49 (d, *J* = 3.8 Hz, 1H), 7.40 – 7.35 (m, 1H), 6.71 (s, 2H). MS (MALDI-TOF): calcd for $C_{40}H_{27}ClN_3O_2S^+$ M^+ 648.15, found 648.42. Due to the poor solubility of HPQ-1-Naph (250 μM) in DMSO, its ^{13}C NMR signals are very low even 10000 times scan (the concentrations of organic compounds for ^{13}C NMR test are typically greater than 10 mM).

Synthesis of HPQ-2-Naph. Compound 8 (50.0 mg, 0.13 mmol) and QL-2-Naph (0.13 mmol) were placed in a round bottom flask, and 10 mL ethanol and a drop of piperidine were added and refluxed at 81 °C for 6 h under nitrogen. After cooling, the reaction solution was dried by rotary evaporator and purified by column chromatography (silica gel, DCM/MeOH = 10:1) to give a black solid 22 mg (Yield 23.2%). ^{1}H NMR (400 MHz, DMSO-d_6) δ 9.53 (d, *J* = 6.7 Hz, 1H), 9.01 (d, *J* = 8.3 Hz, 1H), 8.66 (d, *J* = 2.3 Hz, 1H), 8.56 (d, *J* = 7.2 Hz, 1H), 8.38 (d, *J* = 2.3 Hz, 1H), 8.14 – 8.00 (m, 2H), 7.98 (d, *J* = 7.9

Hz, 2H), 7.95 – 7.91 (m, 2H), 7.87 (s, 2H), 7.79 (d, J = 3.8 Hz, 1H), 7.77 – 7.74 (m, 2H), 7.72 (d, J = 8.7 Hz, 2H), 7.61 (d, J = 3.6 Hz, 1H), 7.55 – 7.53 (m, 2H), 7.50 – 7.35 (m, 1H), 6.83 (d, J = 8.8 Hz, 1H), 6.39 (s, 2H). ^{13}C NMR (101 MHz, DMSO-d_6) δ 164.08, 156.08, 153.55, 152.31, 148.01, 143.66, 141.39, 138.55, 137.50, 135.60, 134.37, 133.18, 133.04, 132.03, 130.81, 130.61, 129.31, 128.81, 128.31, 128.13, 127.24, 127.17, 126.34, 125.68, 125.39, 125.01, 123.91, 122.84, 122.77, 121.09, 119.85, 119.62, 117.38, 115.87, 111.83, 110.73, 104.67, 44.22. MS (MALDI-TOF): calcd for $C_{40}H_{27}ClN_3O_2S^+$ M^+ 648.15, found 648.09.

Synthesis of HPQ-EtOH. Compound 8 (50.0 mg, 0.13 mmol) and QL-EtOH (0.13 mmol) were placed in a round bottom flask, and 10 mL ethanol and a drop of piperidine were added and refluxed at 81 °C for 6 h under nitrogen. After cooling, the reaction solution was dried by rotary evaporator and purified by column chromatography (silica gel, DCM/MeOH = 10:1) to give a black solid 13 mg (Yield 13.7%). ^{1}H NMR (400 MHz, DMSO-d_6) δ 9.33 (d, J = 6.0 Hz, 1H), 8.96 (d, J = 8.5 Hz, 1H), 8.62 (d, J = 2.3 Hz, 1H), 8.45 – 8.41 (m, 2H), 8.31 – 8.22 (m, 3H), 8.08 (s, 1H), 8.05 – 7.99 (m, 1H), 7.96 – 7.87 (m, 1H), 7.83 (d, J = 8.2 Hz, 1H), 7.78 (d, J = 9.5 Hz, 2H), 7.65 (s, 1H), 6.99 (s, 1H), 4.99 – 4.89 (m, 2H), 1.93 – 1.73 (m, 2H). MS (MALDI-TOF): calcd for $C_{31}H_{23}ClN_3O_3S^+$ M^+ 552.11, found 552.07. Due to the poor solubility of HPQ-EtOH (250 μM) in DMSO, its ^{13}C NMR signals are very low even 10000 times scan (the concentrations of organic compounds for ^{13}C NMR test are typically greater than 10 mM).

Synthesis of HPQ-PEG. Compound 8 (50.0 mg, 0.13 mmol) and QL-PEG (0.13 mmol) were placed in a round bottom flask, and 10 mL ethanol and a drop of piperidine were added and refluxed at 81 °C for 6 h under nitrogen. After cooling, the reaction solution was dried by rotary evaporator and purified by column chromatography (silica gel, DCM/MeOH = 10:1) to give a black solid 9 mg (Yield 8.9%). ^{1}H NMR (400 MHz, DMSO-d_6) δ 9.17 (s, 1H), 9.10 – 8.90 (m, 1H), 8.64 (d, J = 2.2 Hz, 1H), 8.56 (d, J = 8.7 Hz, 1H), 8.43 (d, J = 6.3 Hz, 1H), 8.39 (d, J = 2.5 Hz, 1H), 8.28 – 8.20 (m, 1H), 8.06 (dd, J = 7.9, 2.3 Hz, 2H), 7.90 – 7.70 (m, 5H), 7.52 – 7.49 (m, 1H), 6.89 (d, J = 8.7 Hz, 1H), 5.33 – 4.95 (m, 2H), 4.02 – 3.88 (m, 2H), 3.56 – 3.50 (m, 2H), 3.46 – 3.42 (m, 2H), 3.41 – 3.37 (m, 4H), 3.18 (s, 3H), 3.02 (t, J = 5.2 Hz, 2H), 1.67 – 1.54 (m, 2H). MS (MALDI-TOF): calcd for $C_{38}H_{37}ClN_3O_6S^+$ M^+ 698.21, found 698.14. Due to the poor solubility of HPQ-PEG (200 μM) in DMSO, its ^{13}C NMR signals are very low even 10000 times scan (the concentrations of organic compounds for ^{13}C NMR test are typically greater than 10 mM).

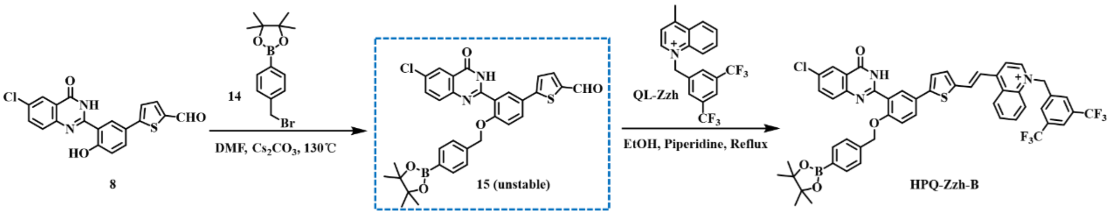

Synthesis of compound HPQ-Zzh-B. A mixture of compound 8 (238.0 mg, 13.0 mmol), compound 14 (118.8 mg, 0.39 mmol, 98% purity) and potassium carbonate (130.0 mg, 0.39 mmol) was dissolved in 3 ml dimethylformamide and then refluxed at 125 °C for 14 h. After cooling, the reaction solution was dried by rotary evaporator and purified by column chromatography (silica gel, DCM) to give a colorless oil which is easy to decompose. Immediately, the colorless oil and QL-Zzh (7.6 mg, 17.0 μmol) were placed in a round bottom flask, and 2 mL ethanol and a drop of piperidine were added and refluxed at 81 °C for 10 min under nitrogen. After cooling, the reaction solution was dried by rotary evaporator and

purified by column chromatography (silica gel, DCM/EtOH = 10:1) to give a red solid 1.1 mg (Yield 0.009%). ^{1}H NMR (400 MHz, DMSO-d_6) δ 9.87 (s, 1H), 9.57 (d, *J* = 6.7 Hz, 1H), 8.96 (d, *J* = 8.7 Hz, 1H), 8.61 (d, *J* = 2.2 Hz, 1H), 8.58 (d, *J* = 2.1 Hz, 1H), 8.55 (s, 1H), 8.41 (d, *J* = 9.1 Hz, 1H), 8.22 (s, 2H), 8.17 (s, 2H), 7.96 – 8.16 (m, 2H), 8.02 (d, *J* = 4.0 Hz, 1H), 7.96 (d, *J* = 4.8 Hz, 1H), 7.82 (s, 1H), 7.81 (s, 1H), 7.78 (d, *J* = 2.4 Hz, 1H), 7.76 (d, *J* = 3.6 Hz, 1H), 7.73 (d, *J* = 3.8 Hz, 1H), 7.71 (d, *J* = 3.9 Hz, 1H), 7.52 (d, *J* = 7.9 Hz, 1H), 7.05 (d, *J* = 8.7 Hz, 2H), 6.41 (s, 2H), 4.71 (s, 2H), 1.30 (s, 12H). ^{13}C NMR (101 MHz, DMSO-d_6) δ 184.22, 161.21, 154.47, 153.66, 148.59, 141.52, 139.71, 138.48, 137.97, 137.33, 135.81, 135.39, 135.16, 134.95, 134.74, 131.94, 131.66, 131.48, 131.37, 131.08, 129.56, 129.10, 128.75, 127.32, 127.17, 127.09, 126.99, 126.12, 125.89, 125.48, 124.89, 122.57, 122.17, 119.67, 117.72, 116.36, 114.86, 114.54, 84.34, 44.16, 22.63. MS (MALDI-TOF): calcd for $C_{51}H_{40}BClF_6N_3O_4S^+$ M^+ 950.24, found 950.50.

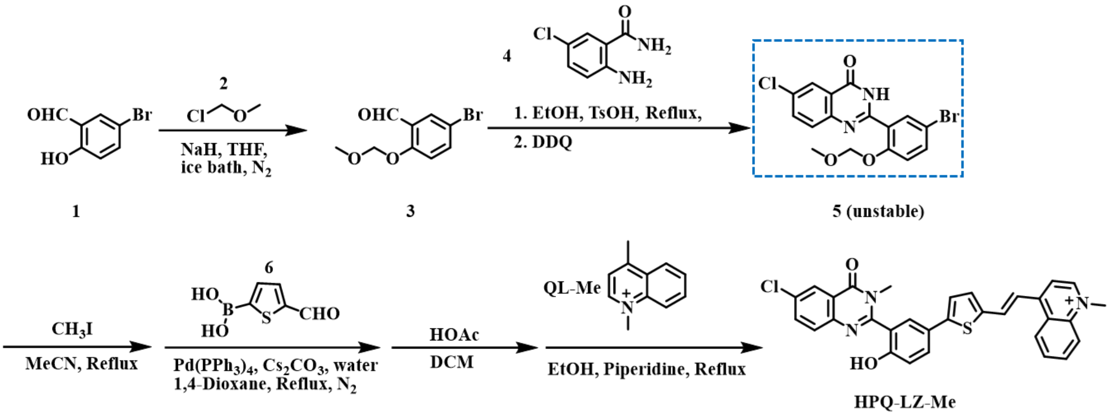

Synthesis of compound HPQ-LZ-Me. Immediately, compound 5 (0.5 g, 1.4 mmol), white solid which is easy to decompose, Cs_2CO_3 (0.1 g, 0.31 mmol) and 10 mL MeCN were placed in a round bottom flask, and excessive CH_3I was added, and stirred at 81 °C for 6 h under nitrogen. After that, the reaction solution was dried by rotary evaporator, and compound 6 (0.33 g, 2.1 mmol, 98% purity), $Pd(PPh_3)_4$ (0.16 g, 0.14 mmol, 98% purity), Cs_2CO_3 (0.6 g, 1.86 mmol) , 10 mL dioxane, 1 mL water were added into the round bottom flask, and stirred at 81 °C for 24 h under nitrogen. Subsequently, the reaction solution was dried by rotary evaporator. After extraction, 1 mL HOAc, 10 mL DCM were added, and used $NaHCO_3$ saturated solution to neutralize excess HOAc. Finally, QL-Me (0.4 g, 1.4 mmol) was placed in a round bottom flask, and 10 mL ethanol and two drop piperidine were added and refluxed at 81 °C for 6 h under nitrogen. After cooling, the reaction solution was dried by rotary evaporator and purified by column chromatography (silica gel, DCM/MeOH = 30:1) to give a black red solid 19.8 mg (Yield 2.1 %). ^{1}H NMR (400 MHz, DMSO-d_6) δ 9.85 (s, 1H), 9.52 (d, *J* = 6.0 Hz, 1H), 8.59 (d, *J* = 8.9 Hz, 1H), 8.56 – 8.45 (m, 1H), 8.43 (d, *J* = 8.3 Hz, 1H), 8.35 – 8.32 (m, 1H), 8.30 (s, 1H), 8.11 – 8.03 (m, 3H), 7.94 (d, *J* = 3.9 Hz, 1H), 7.48 (d, *J* = 4.0 Hz, 1H), 7.47 – 7.42 (m, 1H), 7.40 (d, *J* = 4.0 Hz, 1H), 7.28 (d, *J* = 3.8 Hz, 1H), 7.25 – 7.14 (m, 2H), 4.69 (s, 3H), 3.16 (s, 3H). MS (MALDI-TOF): calcd for $C_{31}H_{23}ClN_3O_2S^+$ M^+ 536.12, found 536.15.

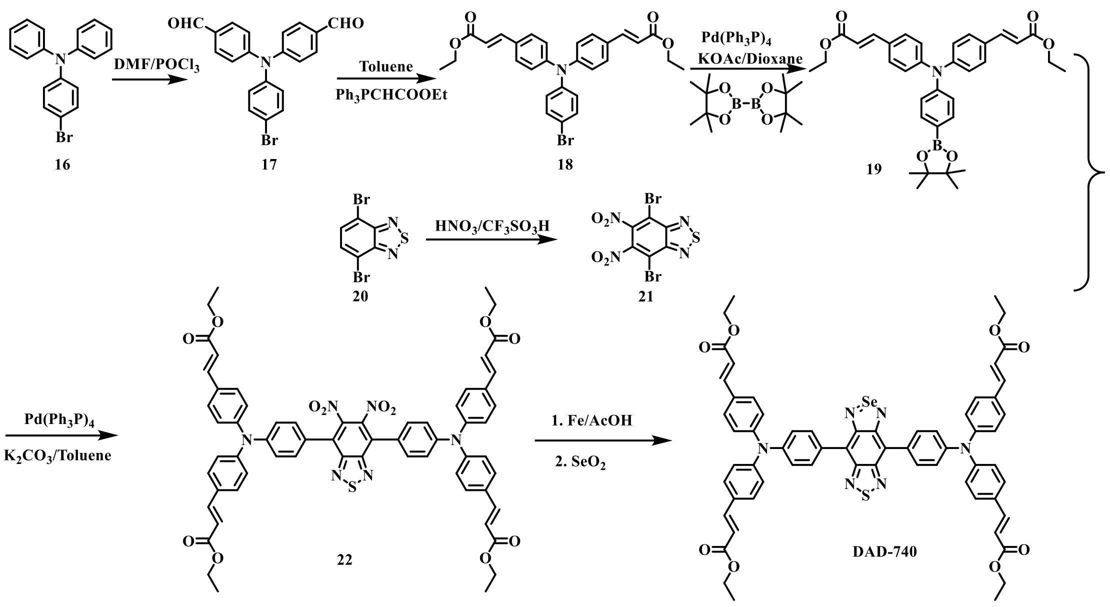

Synthesis of compound 17. $POCl_3$ (76.7g, 500 mmol) was added dropwise to DMF (39.0 mL, 500 mmol) under N_2 at 0 °C. The mixture was stirred for 1h. Then compound 16 (6.5 g, 20.0 mmol, 98% purity) was added and the solution was stirred at 80 °C for 4h. After cooling to R.T., the resulting dark red suspension was washed with water and extracted with DCM twice. The crude product purified by column chromatography (silica gel, DCM/PE= 1:3) to give solid 3.6 g (Yield 2.0%). ^{1}H NMR (400 MHz, Chloroform-*d*) δ 9.91 (s, 2H), 7.80 (d, *J* = 7.9 Hz, 4H), 7.51 (d, *J* = 7.7 Hz, 2H), 7.19 (d, *J* = 7.9 Hz, 4H), 7.06 (d, *J* = 7.6 Hz, 2H). ^{13}C NMR (101 MHz, $CDCl_3$) δ 190.55, 151.59, 144.64, 133.28, 131.59, 128.31, 123.00, 119.23. MS (MALDI-TOF, HRMS): calcd for $C_{20}H_{14}BrNO_2^+$ M$^+$ 379.02, found 379.02.

Synthesis of compound 18. $Ph_3PCHCOOEt$ (2.6 g, 7.5 mmol, 98% purity) was added to a solution of compound 17 (1.3 g, 3.41 mmol) in toluene under N_2. The solution was stirred for 48 h at R.T. and dried by rotary evaporator and purified by column chromatography (silica gel, PE: EtOAc =15:1) to give a green solid 1.4 g (Yield 79%). ^{1}H NMR (400 MHz, Chloroform-*d*) δ 7.63 (d, *J* = 7.4 Hz, 2H), 7.42 (d, *J* = 7.8 Hz, 6H), 7.13 – 6.93 (m, 6H), 6.33 (d, *J* = 7.5 Hz, 2H), 4.26 (q, *J* = 6.8 Hz, 4H), 1.34 (t, *J* = 6.9 Hz, 6H). ^{13}C NMR (101 MHz, $CDCl_3$) δ 167.14, 148.42, 145.54, 143.66, 132.74, 129.43, 129.36, 127.04, 123.61, 117.31, 116.76, 60.43, 14.36. MS (MALDI-TOF, HRMS): calcd for $C_{28}H_{26}BrNO_4^+$ M$^+$ 519.10, found 519.10.

Synthesis of compound 19. To a solution of $Pd(PPh_3)_4$ (0.18 mmol, 98% purity), KOAc (0.24 g, 2.4 mmol) and pinacol diborate (0.30 g, 1.2 mmol, 98% purity) in dioxane was added compound 18 (0.52 g, 1.0 mmol) under N_2. The reaction mixture was heated 80 °C for 12 h. The solution was cooled, dilute with water (40 mL) and extracted with EtOAc. The mixture was dried by rotary evaporator and purified by column chromatography (silica gel, PE: EtOAc =15:1) to give a green solid 0.43 g (Yield 76%). ^{1}H NMR (400 MHz, Chloroform-*d*) δ 7.66 (d, *J* = 7.5 Hz, 2H), 7.55 (d, *J* = 7.6 Hz, 2H), 7.33 (d, *J* = 7.7 Hz, 4H), 7.01 (t, *J* = 9.0 Hz, 6H), 6.25 (d, *J* = 7.7 Hz, 2H), 4.17 (q, *J* = 6.7, 6.2 Hz, 4H), 1.26 (s, 18H). ^{13}C NMR (101 MHz, $CDCl_3$) δ 167.23, 148.58, 143.82, 136.22, 129.31, 124.00, 116.58, 83.82, 60.44, 24.89, 14.39. MS (MALDI-TOF, HRMS): calcd for $C_{34}H_{38}BNO_6^+$ M$^+$ 567.28, found 567.28.

Synthesis of compound 22. Compound 19 (0.50 g, 0.88 mmol), Compound 21 (0.17 g, 0.44 mmol),

K_2CO_3 (0.41 g, 3 mmol), $Pd(PPh_3)_4$ (0.44 mmol, 98% purity), K_2CO_3 (0.41 g, 3 mmol) were placed in a round bottom flask and toluene 20 mL was added and reflux for 48 h under N_2. After extraction, the mixture was dried by rotary evaporator and purified by column chromatography (silica gel, PE: EtOAc =10:1) to give a black solid 0.31 g (Yield 61%). ^{1}H NMR (400 MHz, Chloroform-*d*) δ 7.72 – 7.60 (m, 6H), 7.48 (t, *J* = 6.6 Hz, 12H), 7.25 – 7.05 (m, 10H), 6.47 – 6.32 (m, 4H), 4.31 – 4.23 (m, 8H), 1.35 (t, *J* = 5.9 Hz, 12H). ^{13}C NMR (101 MHz, $CDCl_3$) δ 167.26, 153.03, 148.71, 148.06, 143.60, 142.45, 131.91, 130.56, 129.53, 127.91, 124.94, 123.59, 117.25, 29.71, 14.36. MS (MALDI-TOF, HRMS): calcd for $C_{62}H_{52}N_6O_{12}S^+$ M^+ 1105.19, found 1105.46.

Synthesis of DAD-740. Compound 22 (0.50 g, 0.45 mmol) reacted with iron powder (0.25 g, 4.5 mmol) in acetic acid for 5 h at 100 °C. After extraction, the crude product is oxidized with SeO_2, and purified by column chromatography (silica gel, DCM) to obtain green solid 20.0 mg (Yield 4.3%). ^{1}H NMR (400 MHz, Chloroform-*d*) δ 8.19 (d, *J* = 7.7 Hz, 4H), 7.67 (d, *J* = 7.9 Hz, 4H), 7.49 (d, *J* = 7.8 Hz, 8H), 7.36 (d, *J* = 7.8 Hz, 4H), 7.24 (s, 8H), 6.37 (d, *J* = 7.8 Hz, 4H), 4.27 (q, *J* = 6.7 Hz, 8H), 1.35 (t, *J* = 6.8 Hz, 12H). ^{13}C NMR (101 MHz, $CDCl_3$) δ 167.20, 158.77, 152.78, 148.52, 146.79, 143.77, 133.42, 131.42, 129.74, 129.43, 124.50, 123.94, 120.27, 116.83, 60.46, 14.37. MS (MALDI-TOF, HRMS): calcd for $C_{62}H_{52}N_6O_8SSe$ M^+ 1120.27, found 1120.46.

4. MS Spectra and NMR spectra.

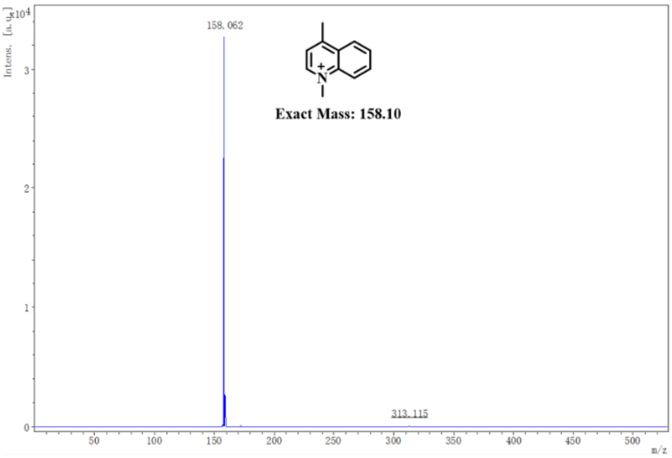

Supplementary Figure | MALDI-TOF mass spectrum of compound QL-Me.

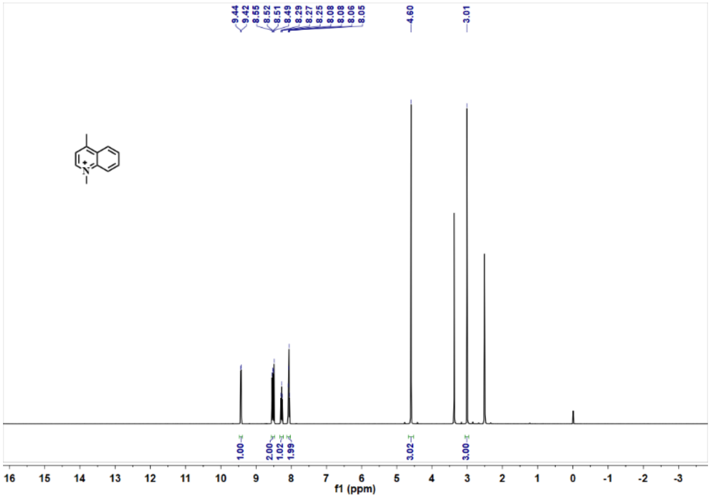

Supplementary Figure | ^{1}H-NMR spectrum of QL-Me in DMSO-d_6.

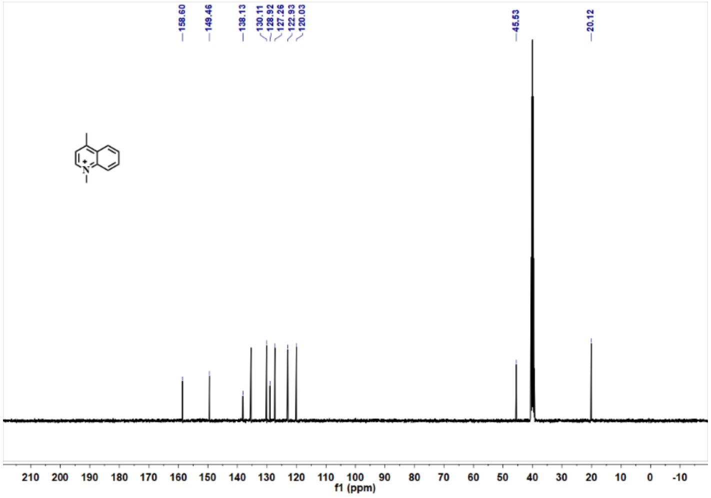

Supplementary Figure | ^{13}C-NMR spectrum of QL-Me in DMSO-d_6.

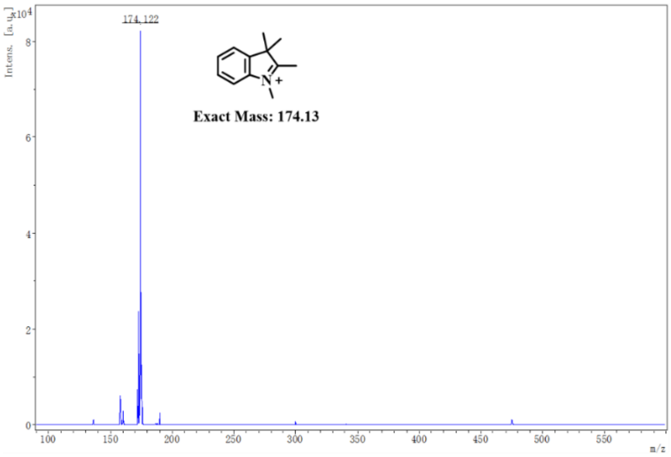

Supplementary Figure | MALDI-TOF mass spectrum of compound 9.

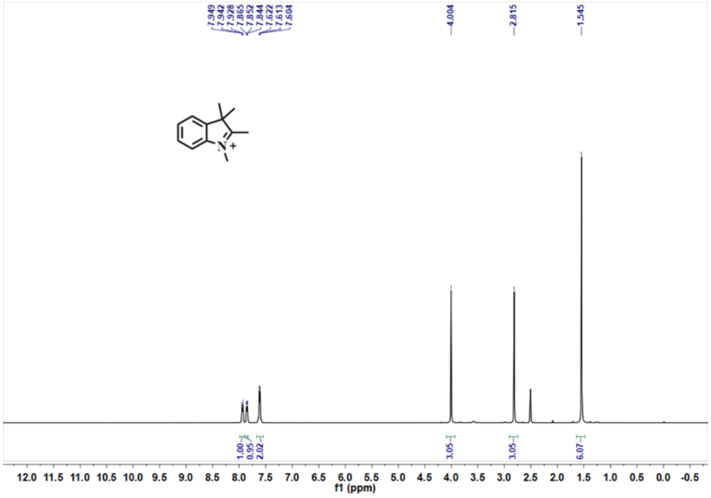

Supplementary Figure | ^{1}H-NMR spectrum of compound 9 in DMSO-d_6.

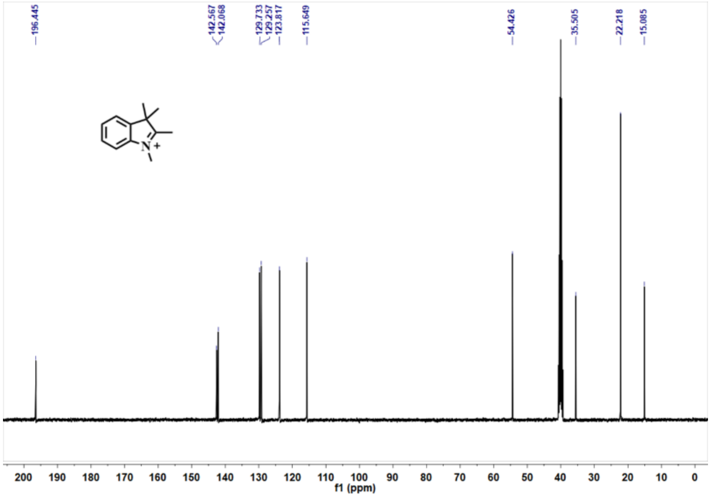

Supplementary Figure | ^{13}C-NMR spectrum of compound 9 in DMSO-d_6.

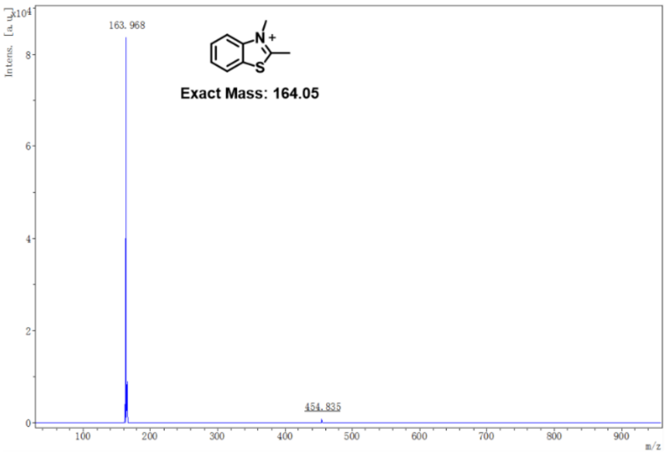

Supplementary Figure | MALDI-TOF mass spectrum of compound 10.

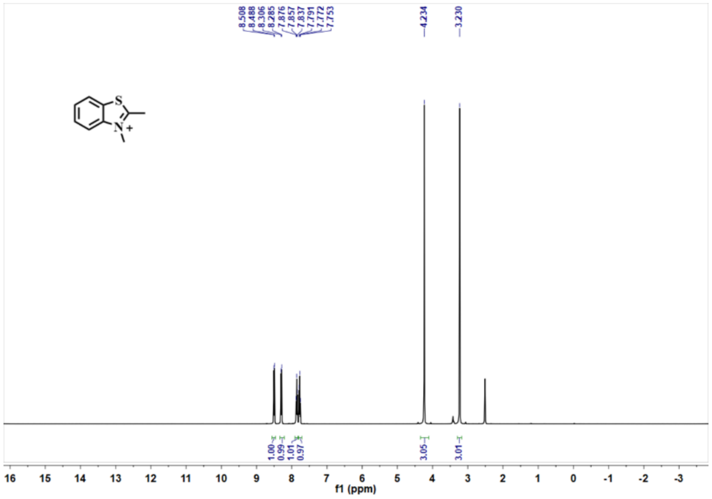

Supplementary Figure | ^{1}H-NMR spectrum of compound 10 in DMSO-d_6.

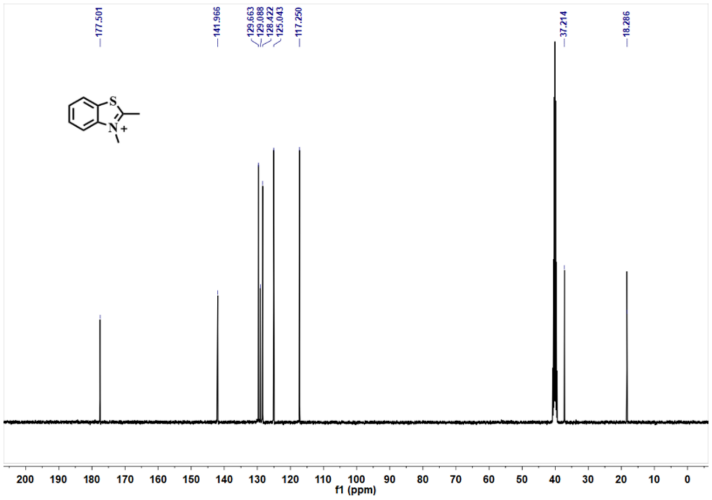

Supplementary Figure | ^{13}C-NMR spectrum of compound 10 in DMSO-d_6.

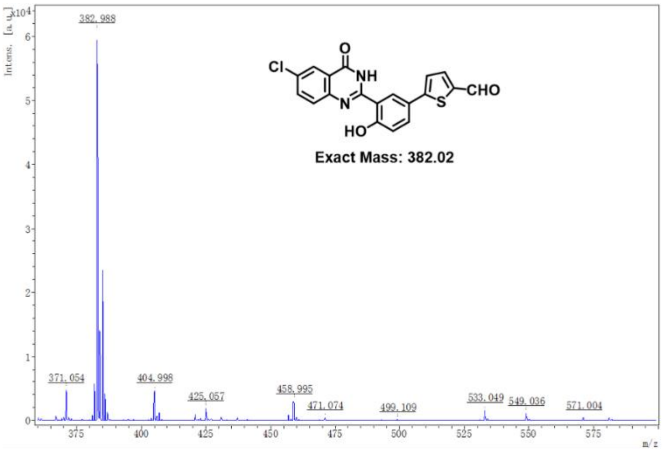

Supplementary Figure | MALDI-TOF mass spectrum of compound 8.

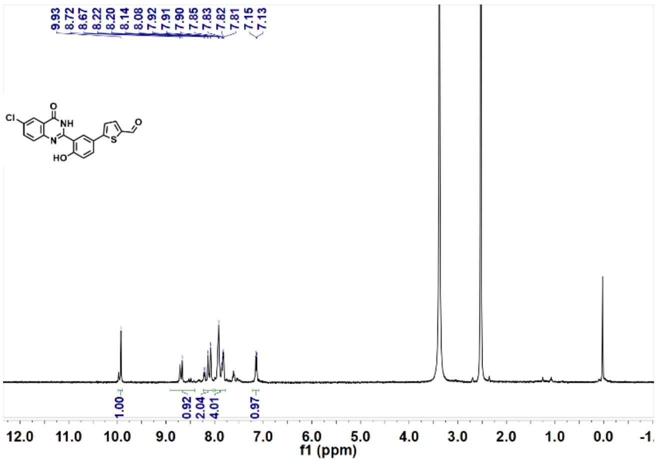

Supplementary Figure | ^{1}H-NMR spectrum of compound 8 in DMSO-d_6.

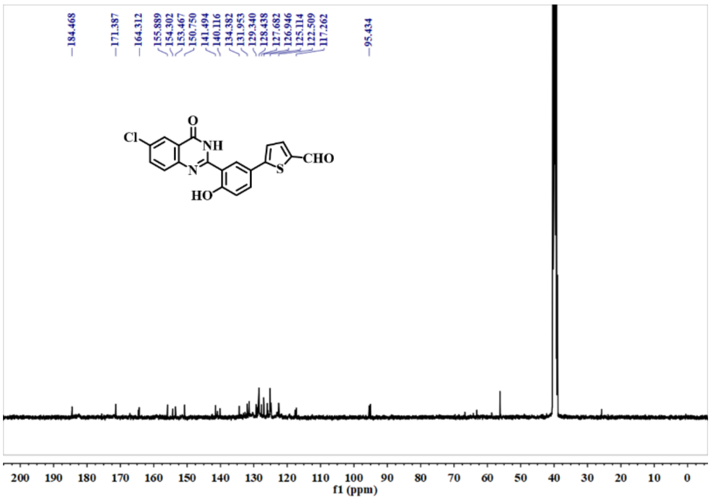

Supplementary Figure | ^{13}C-NMR spectrum of compound 8 in DMSO-d_6.

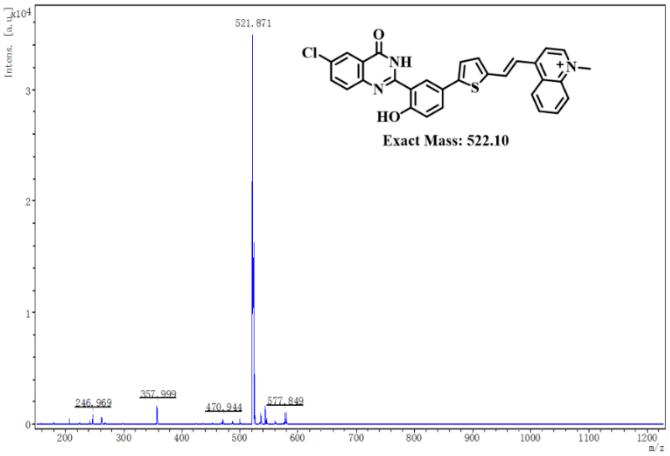

Supplementary Figure | MALDI-TOF mass spectrum of HPQ-LZ.

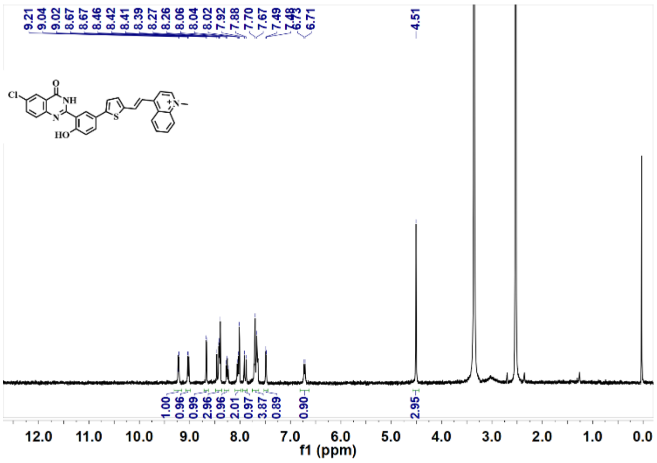

Supplementary Figure | ^{1}H-NMR spectrum of HPQ-LZ in DMSO-d_6.

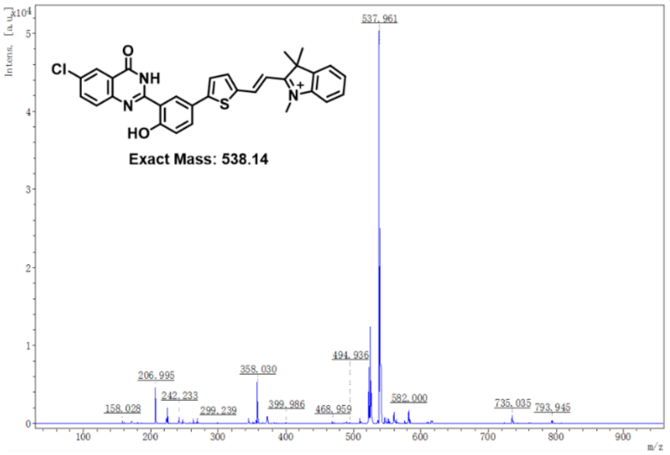

Supplementary Figure | MALDI-TOF mass spectrum of HPQ-1.

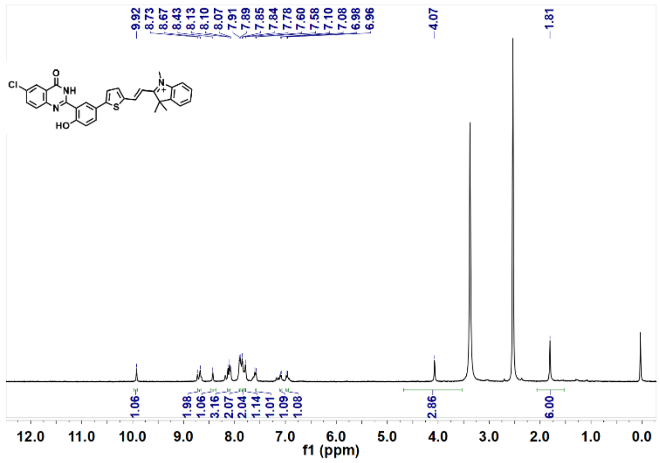

Supplementary Figure | ^{1}H-NMR spectrum of HPQ-1 in DMSO-d_6.

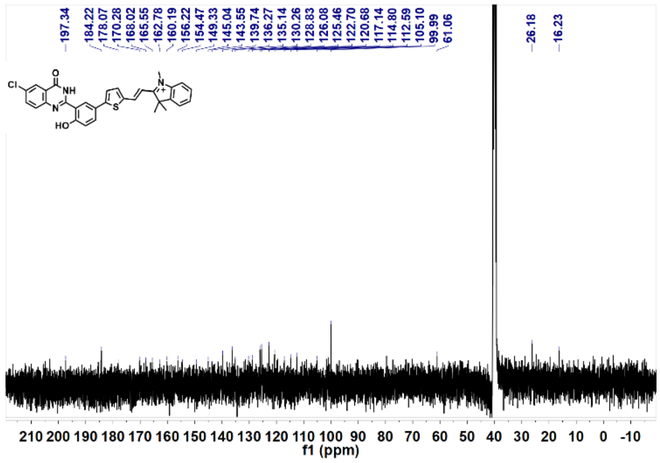

Supplementary Figure | ^{13}C-NMR spectrum of HPQ-1 in DMSO-d_6.

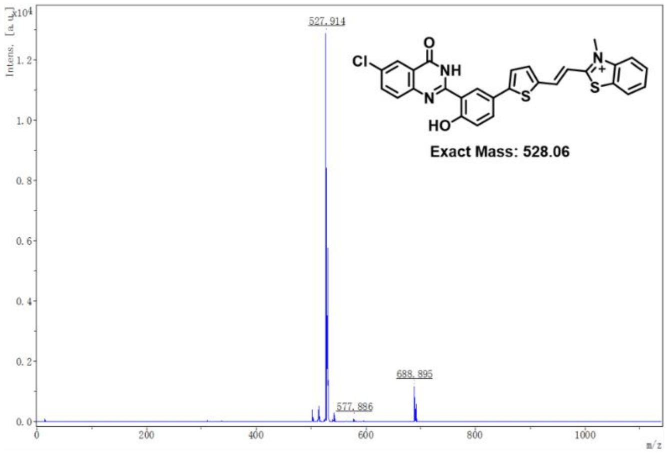

Supplementary Figure | MALDI-TOF mass spectrum of HPQ-2.

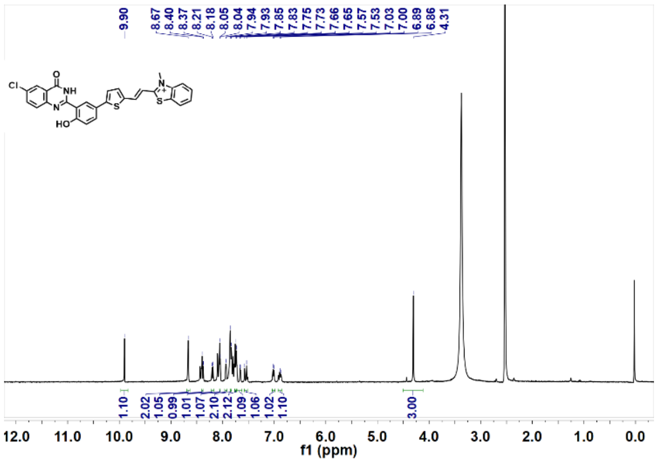

Supplementary Figure | ^{1}H-NMR spectrum of HPQ-2 in DMSO-d_6.

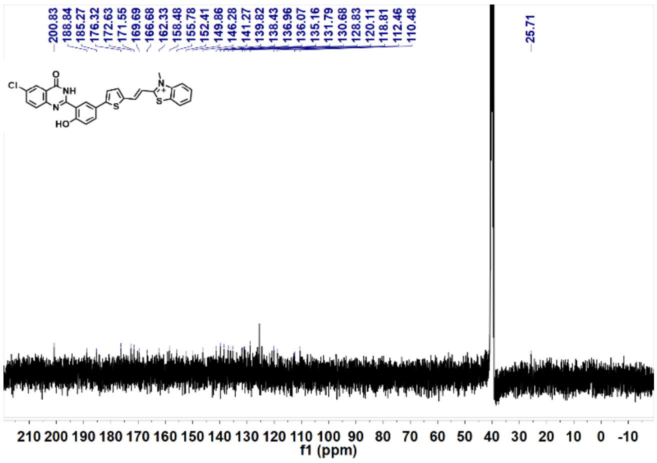

Supplementary Figure | ^{13}C-NMR spectrum of HPQ-2 in DMSO-d_6.

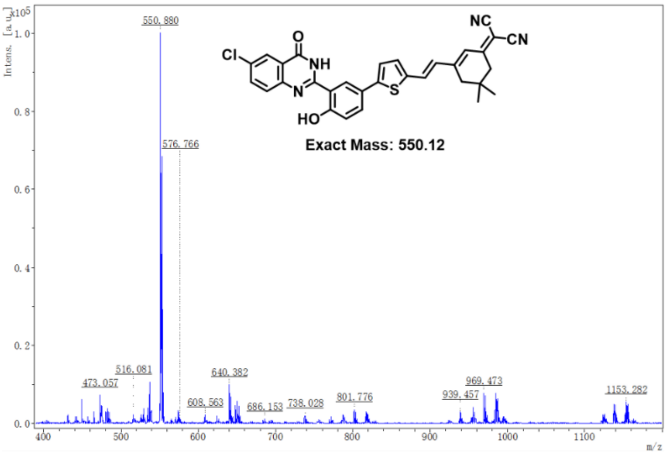

Supplementary Figure | MALDI-TOF mass spectrum of HPQ-3.

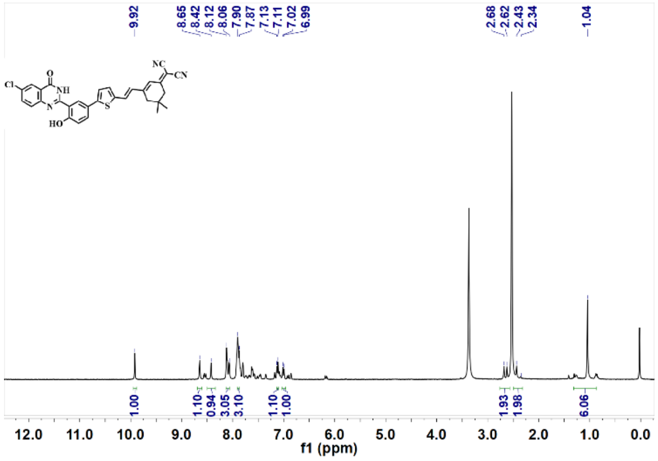

Supplementary Figure | ^{1}H-NMR spectrum of HPQ-3 in DMSO-d_6.

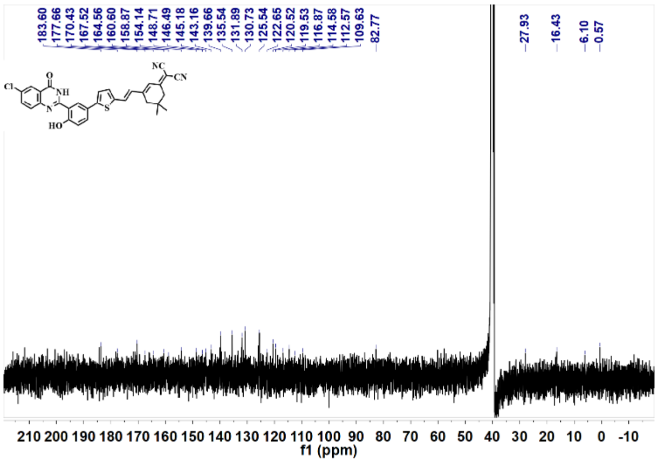

Supplementary Figure | ^{13}C-NMR spectrum of HPQ-3 in DMSO-d_6.

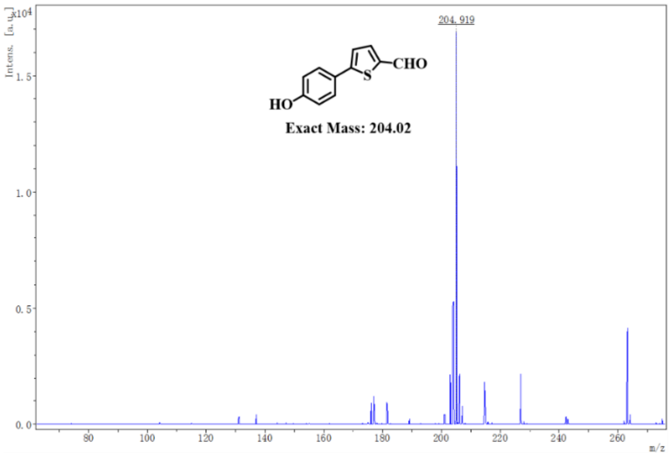

Supplementary Figure | MALDI-TOF mass spectrum of compound 13.

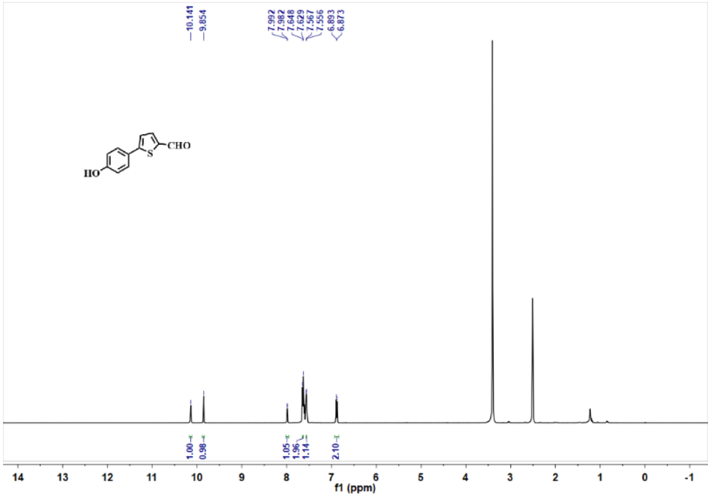

Supplementary Figure | ^{1}H-NMR spectrum of compound 13 in DMSO-d_6.

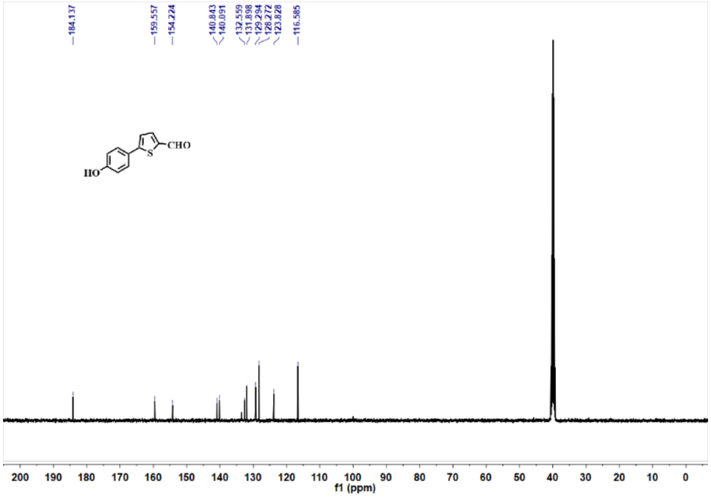

Supplementary Figure | ^{13}C-NMR spectrum of compound 13 in DMSO-d_6.

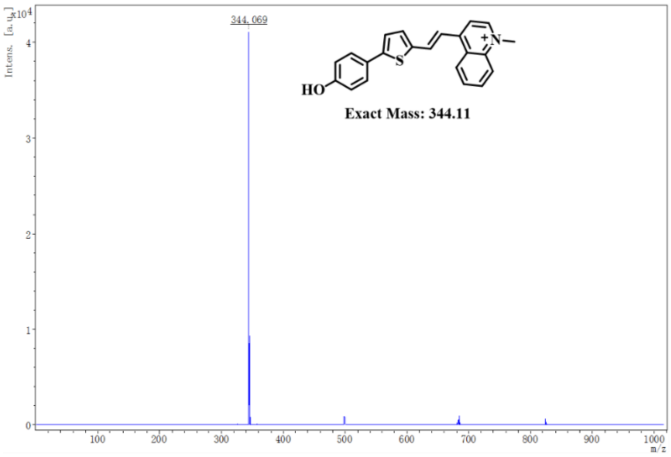

Supplementary Figure | MALDI-TOF mass spectrum of HP-LZ.

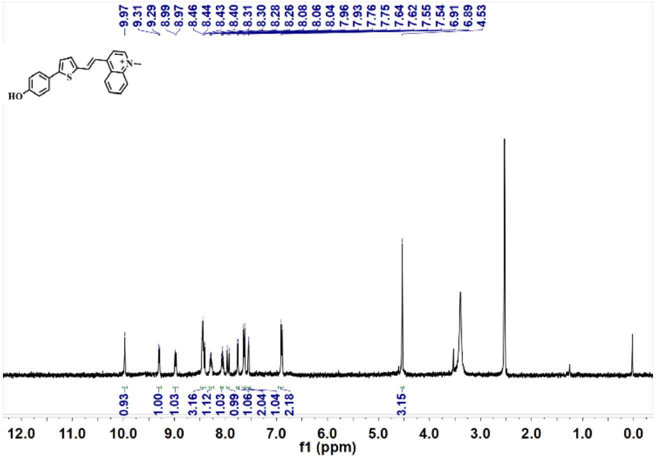

Supplementary Figure | ^{1}H-NMR spectrum of HP-LZ in DMSO-d_6.

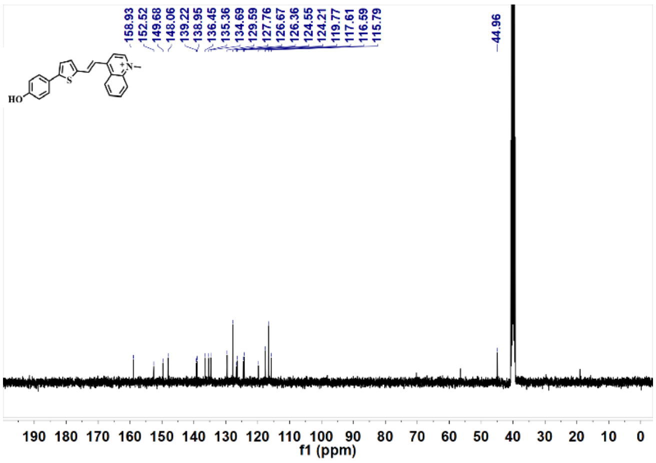

Supplementary Figure | ^{13}C-NMR spectrum of HP-LZ in DMSO-d_6.

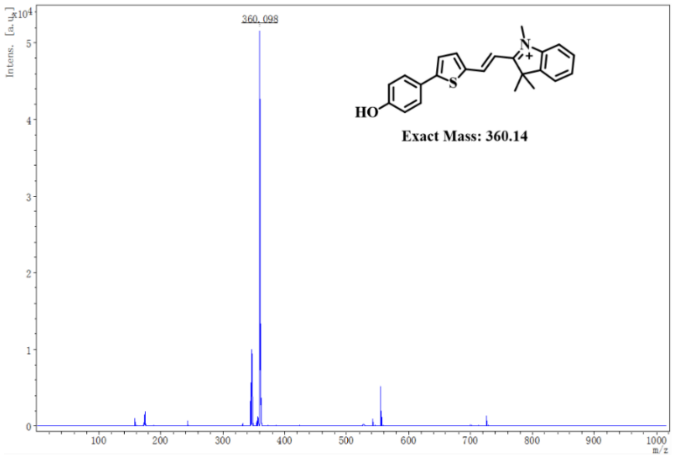

Supplementary Figure | MALDI-TOF mass spectrum of HP-1.

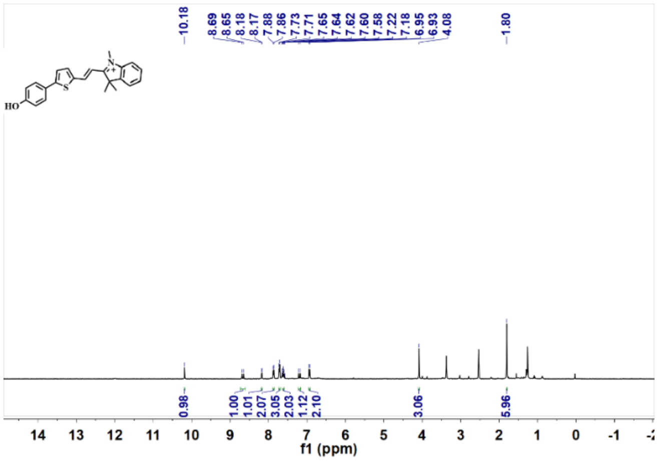

Supplementary Figure | ^{1}H-NMR spectrum of HP-1 in DMOS-d_6.

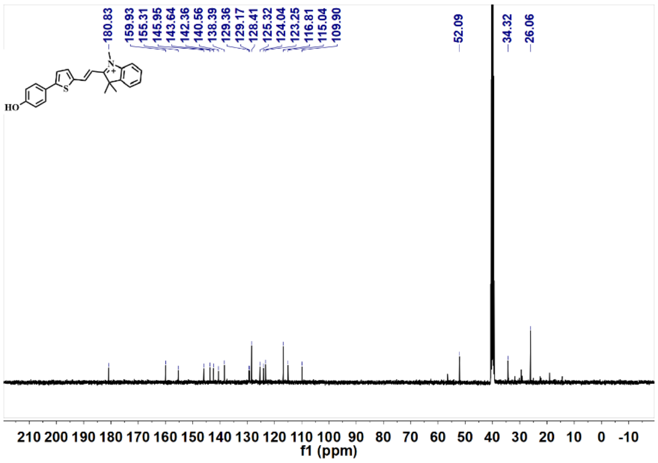

Supplementary Figure | ^{13}C-NMR spectrum of HP-1 in DMOS-d_6.

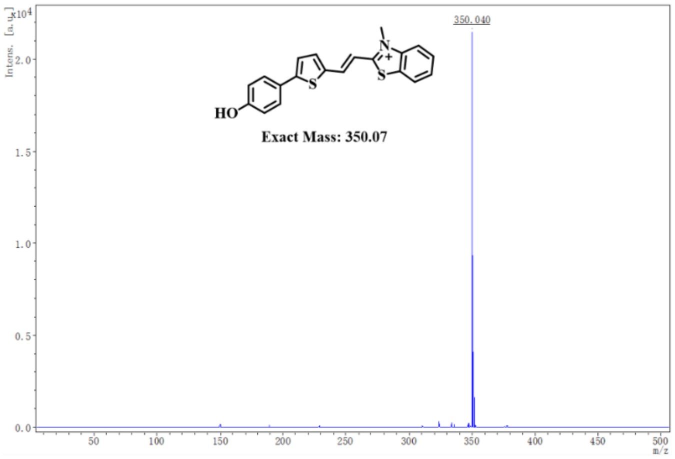

Supplementary Figure | MALDI-TOF mass spectrum of HP-2.

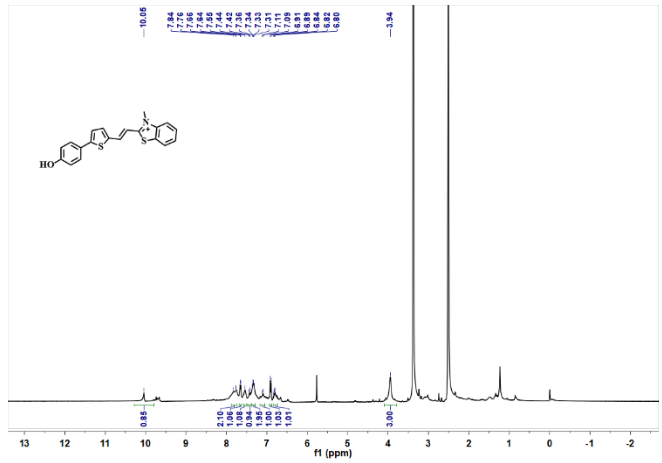

Supplementary Figure | ^{1}H-NMR spectrum of HP-2 in DMSO-d_6.

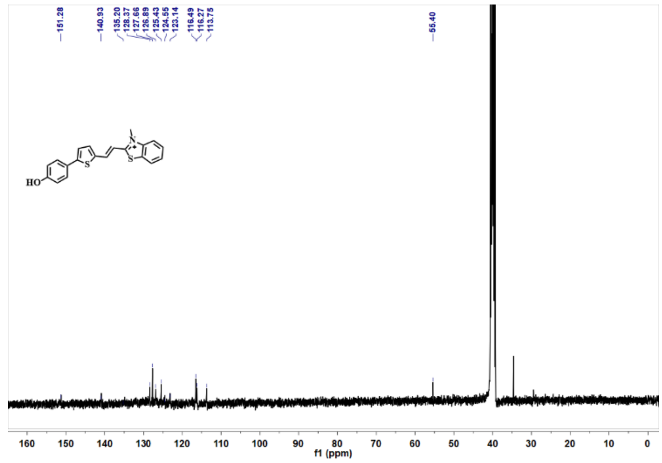

Supplementary Figure | ^{13}C-NMR spectrum of HP-2 in DMSO-d_6.

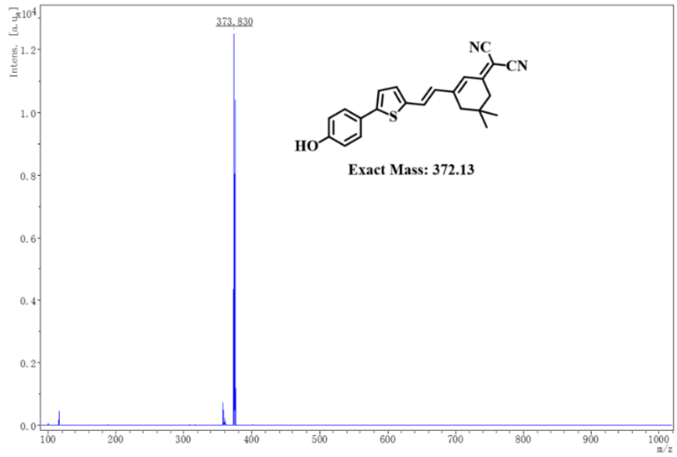

Supplementary Figure | MALDI-TOF mass spectrum of HP-3.

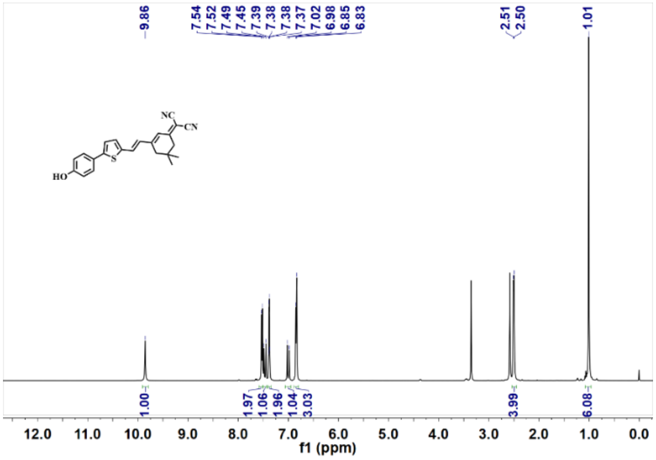

Supplementary Figure | ^{1}H-NMR spectrum of HP-3 in DMSO-d_6.

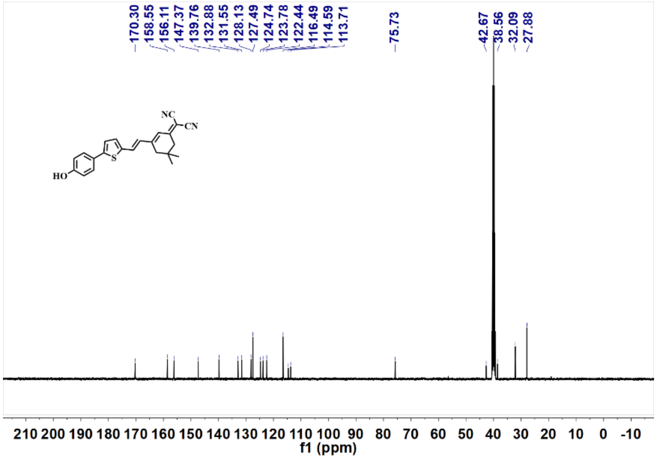

Supplementary Figure | ^{13}C-NMR spectrum of HP-3 in DMSO-d_6.

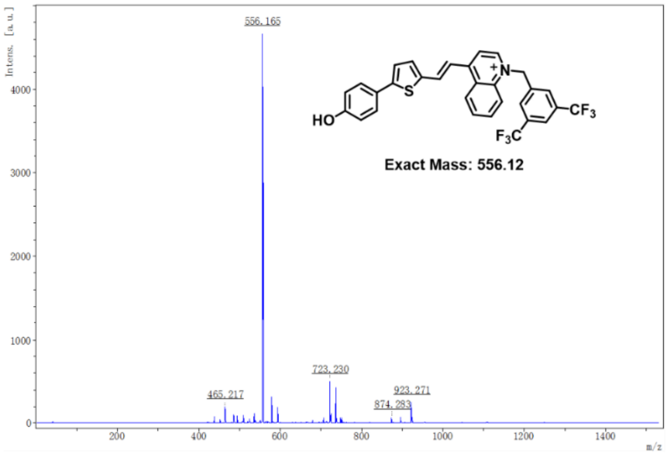

Supplementary Figure | MALDI-TOF mass spectrum of HP-Zzh.

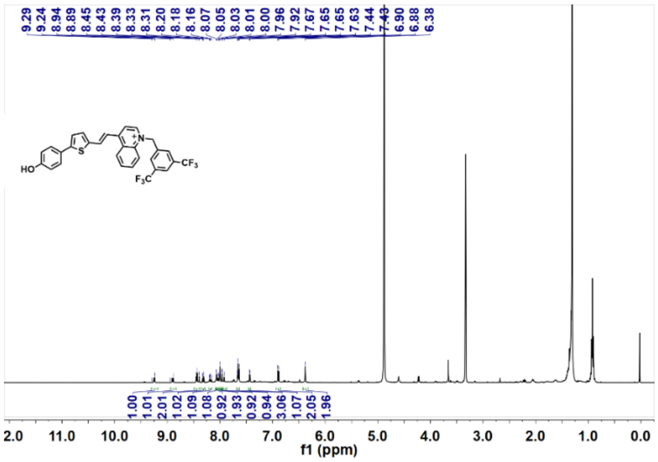

Supplementary Figure | ^{1}H-NMR spectrum of HP-Zzh in DMSO-d_6.

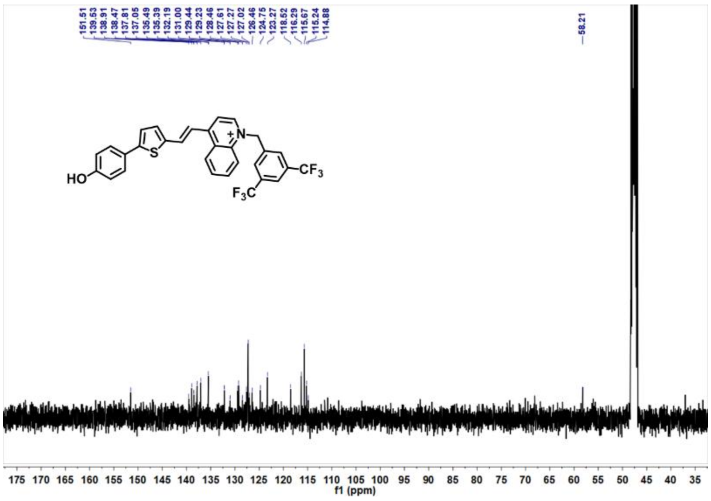

Supplementary Figure | ^{13}C-NMR spectrum of HP-Zzh in DMSO-d_6.

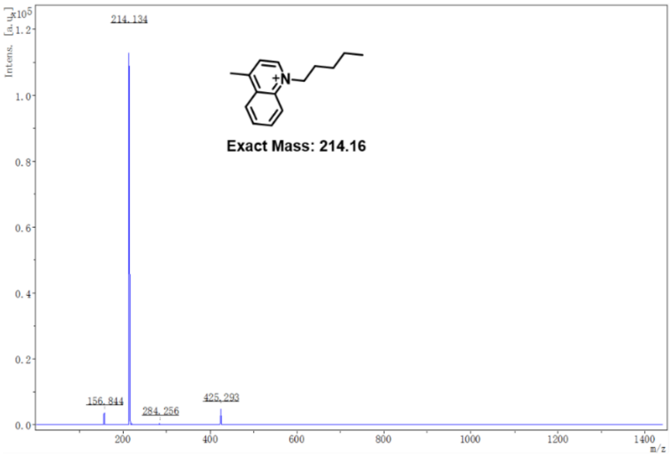

Supplementary Figure | MALDI-TOF mass spectrum of QL-n5C.

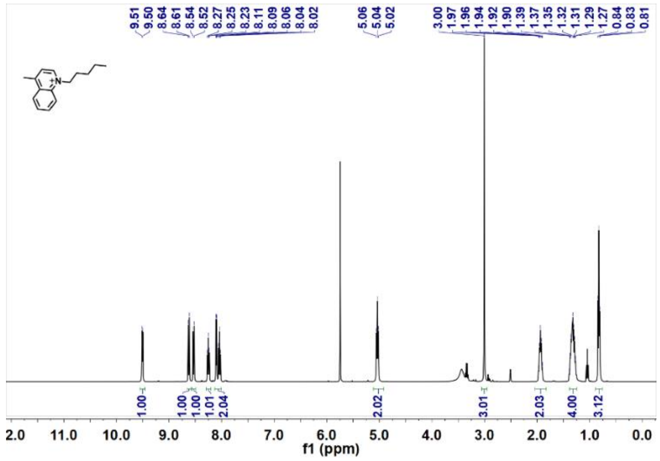

Supplementary Figure | ^{1}H-NMR spectrum of QL-n5C in DMSO-d_6.

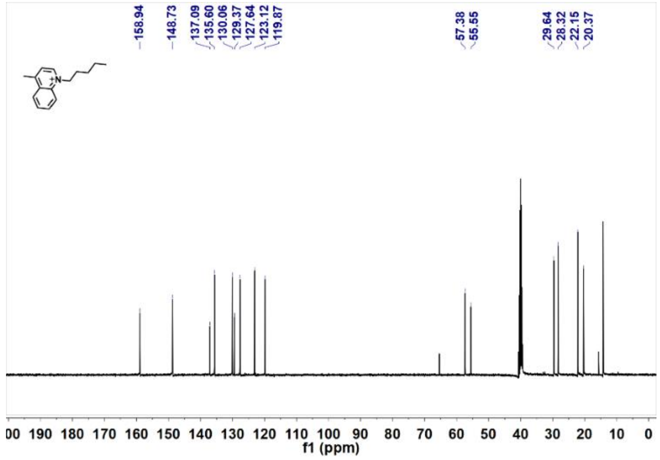

Supplementary Figure | ^{13}C-NMR spectrum of QL-n5C in DMSO-d_6.

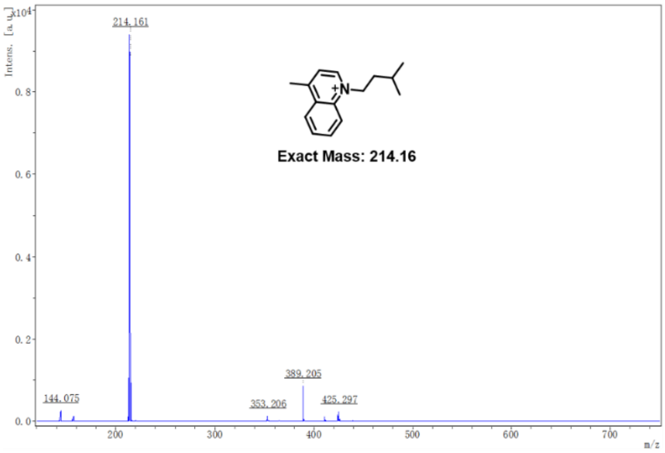

Supplementary Figure | MALDI-TOF mass spectrum of QL-i5C.

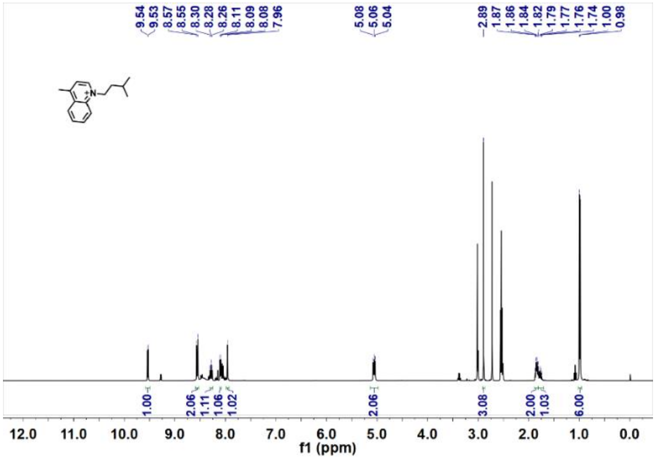

Supplementary Figure | ^{1}H-NMR spectrum of QL-i5C in DMSO-d_6.

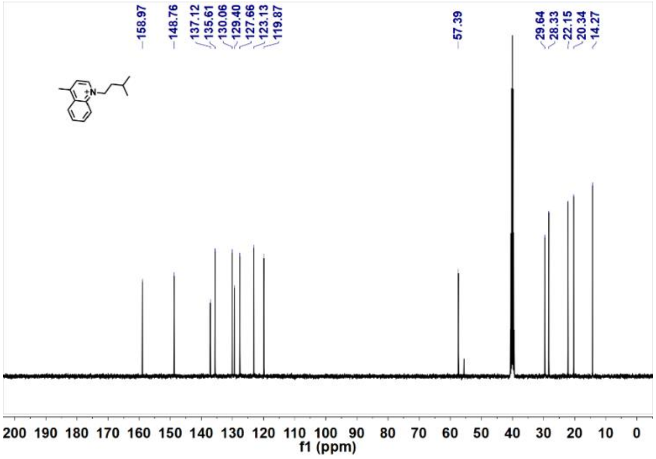

Supplementary Figure | ^{13}C-NMR spectrum of QL-i5C in DMSO-d_6.

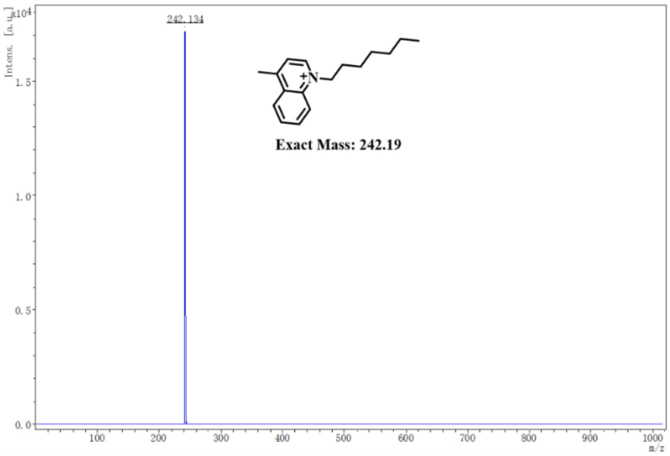

Supplementary Figure | MALDI-TOF mass spectrum of QL-n7C.

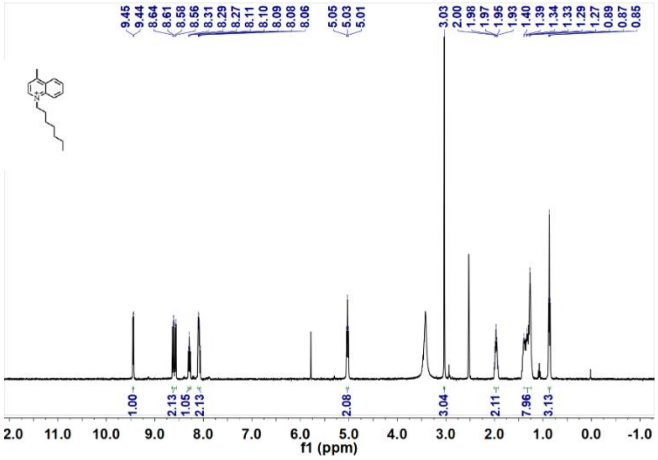

Supplementary Figure | ^{1}H-NMR spectrum of QL-n7C in DMSO-d_6.

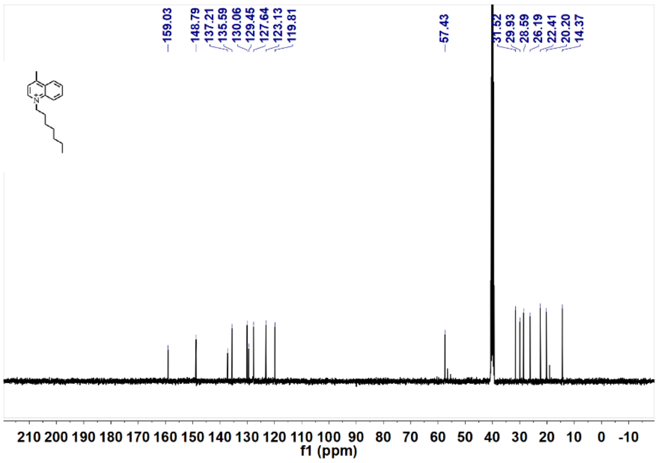

Supplementary Figure | ^{13}C-NMR spectrum of QL-n7C in DMSO-d_6.

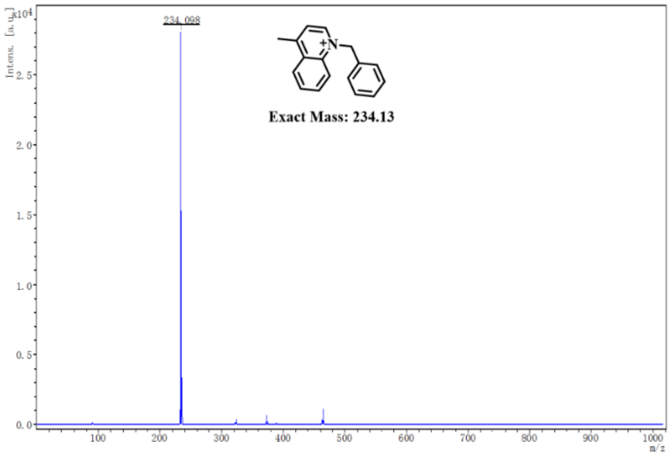

Supplementary Figure | MALDI-TOF mass spectrum of QL-Ph.

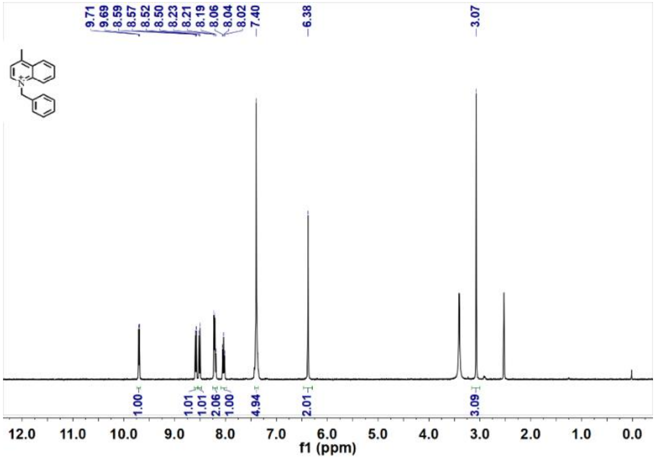

Supplementary Figure | ^{1}H-NMR spectrum of QL-Ph in DMSO-d_6.

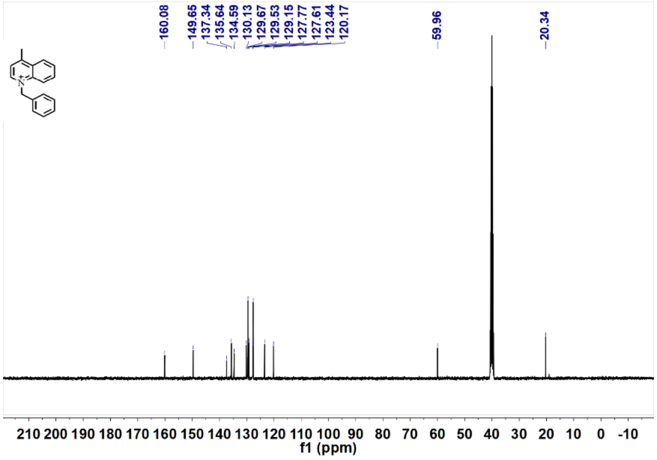

Supplementary Figure | ^{13}C-NMR spectrum of QL-Ph in DMSO-d_6.

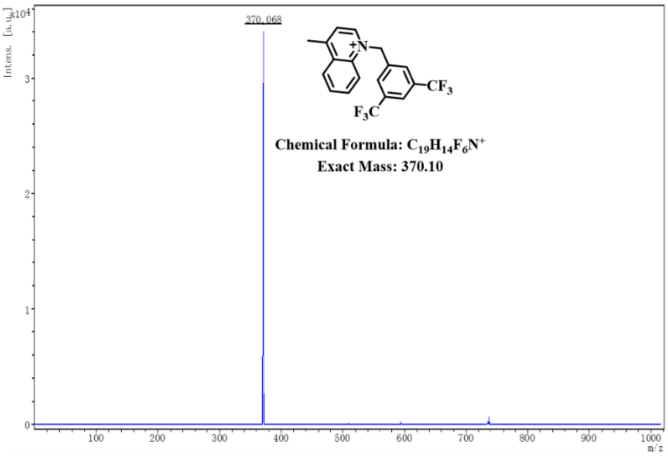

Supplementary Figure | MALDI-TOF mass spectrum of QL-Ph.

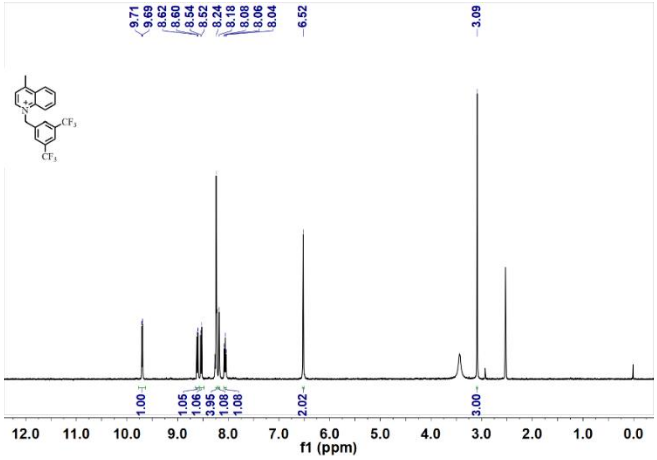

Supplementary Figure | ^{1}H-NMR spectrum of QL-Zzh in DMSO-d_6.

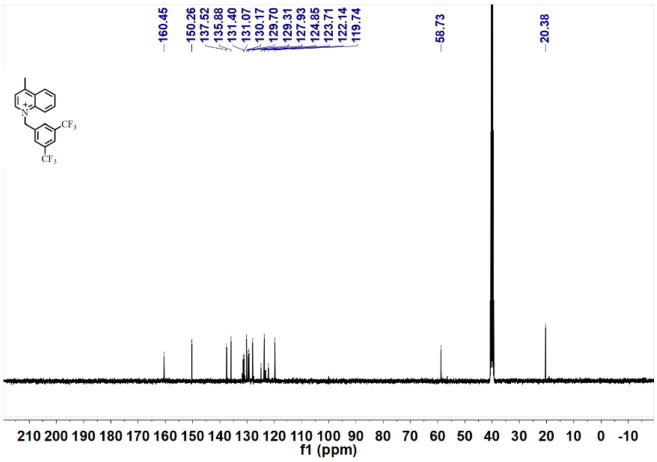

Supplementary Figure |^{13}C-NMR spectrum of QL-Zzh in DMSO-d_6.

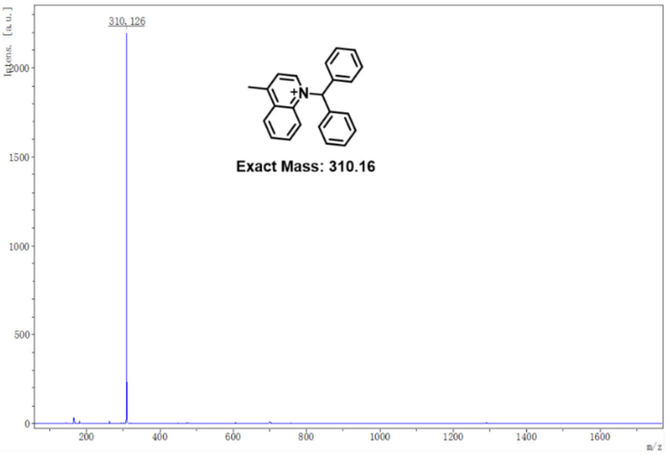

Supplementary Figure | MALDI-TOF mass spectrum of QL-LPZ.

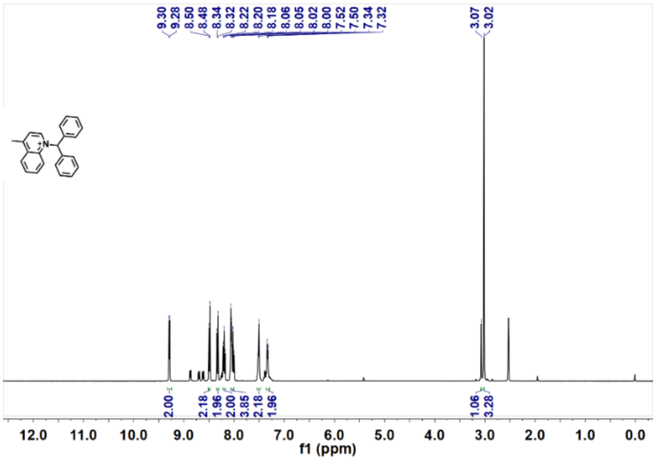

Supplementary Figure | ^{1}H-NMR spectrum of QL-LPZ in DMSO-d_6.

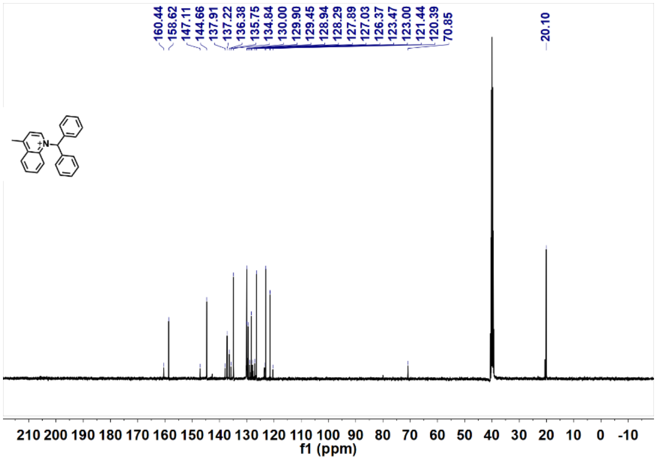

Supplementary Figure | ^{13}C-NMR spectrum of QL-LPZ in DMSO-d_6.

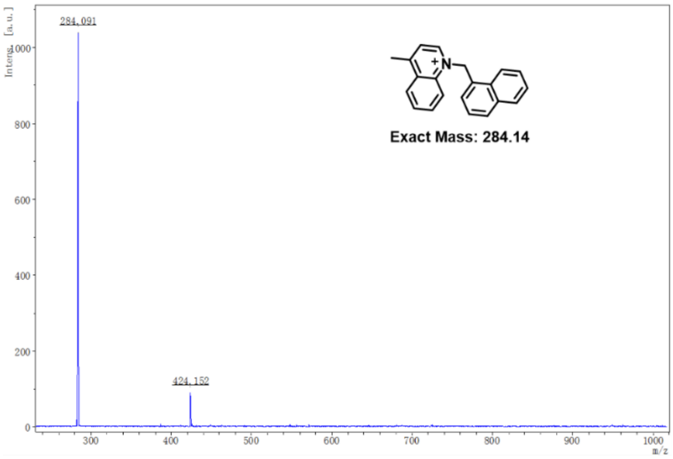

Supplementary Figure | MALDI-TOF mass spectrum of QL-1-Naph.

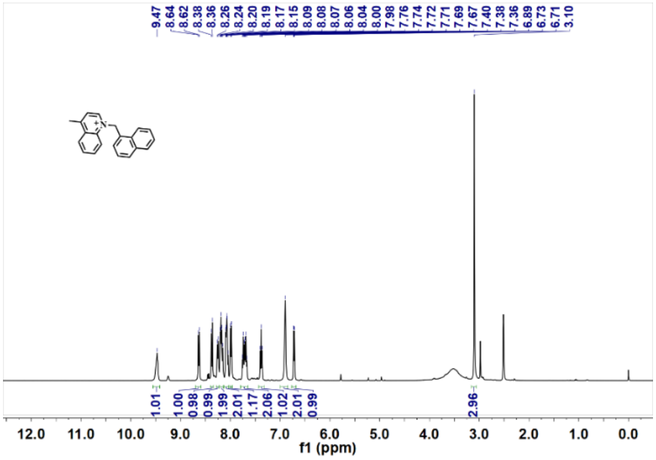

Supplementary Figure | ^{1}H-NMR spectrum of QL-1-Naph in DMSO-d_6.

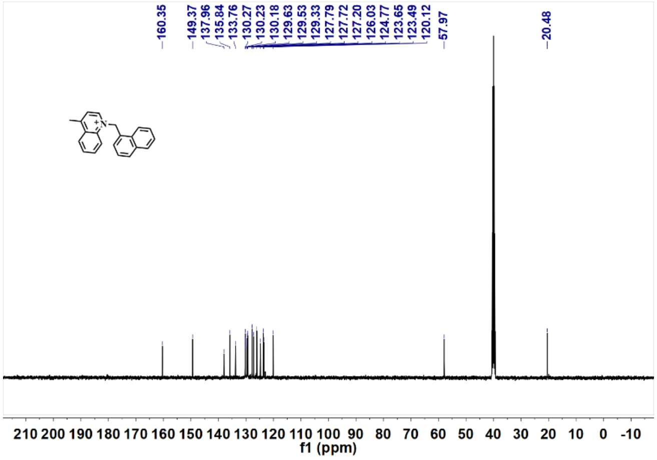

Supplementary Figure | ^{13}C-NMR spectrum of QL-1-Naph in DMSO-d_6.

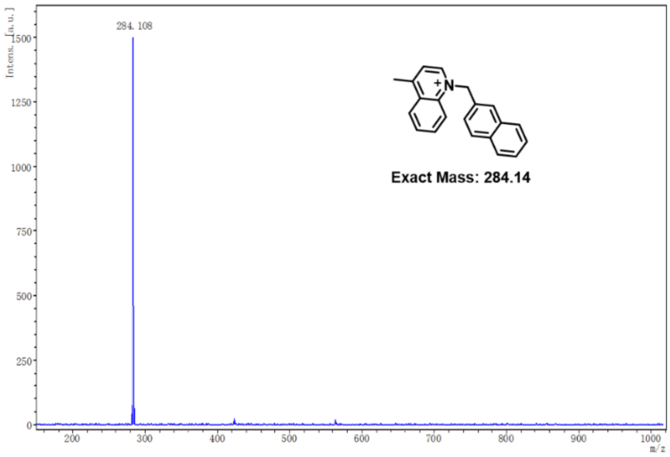

Supplementary Figure | MALDI-TOF mass spectrum of QL-2-Naph.

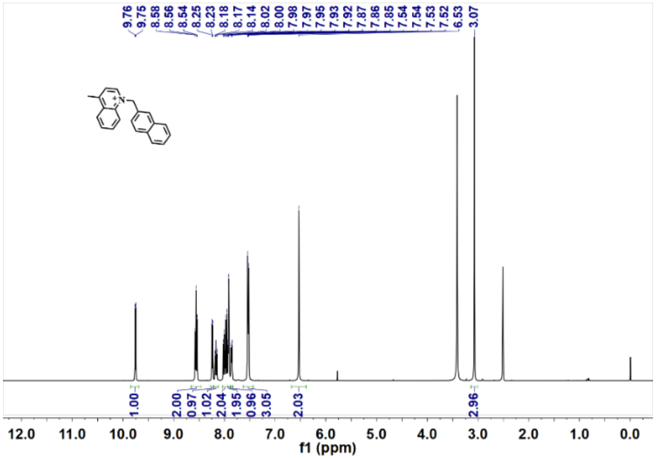

Supplementary Figure | ^{1}H-NMR spectrum of QL-2-Naph in DMSO-d_6.

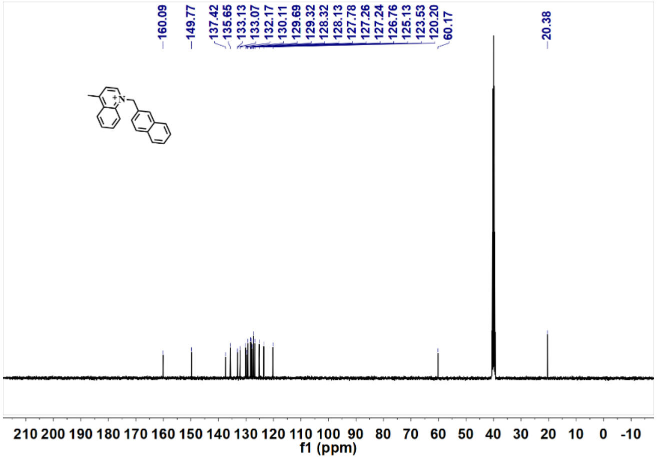

Supplementary Figure | ^{13}C-NMR spectrum of QL-2-Naph in DMSO-d_6.

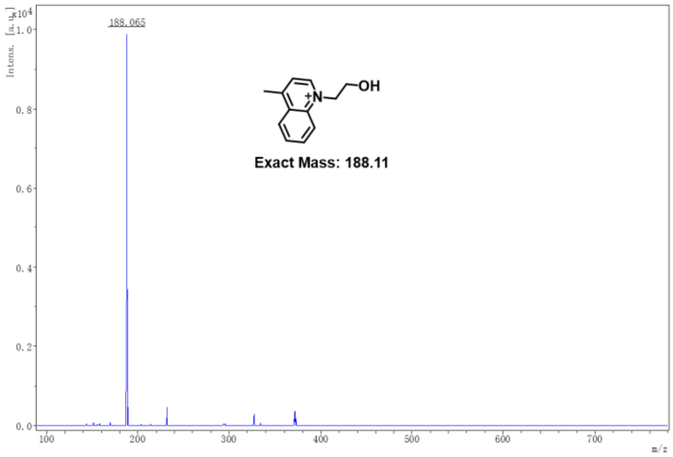

Supplementary Figure | MALDI-TOF mass spectrum of QL-EtOH.

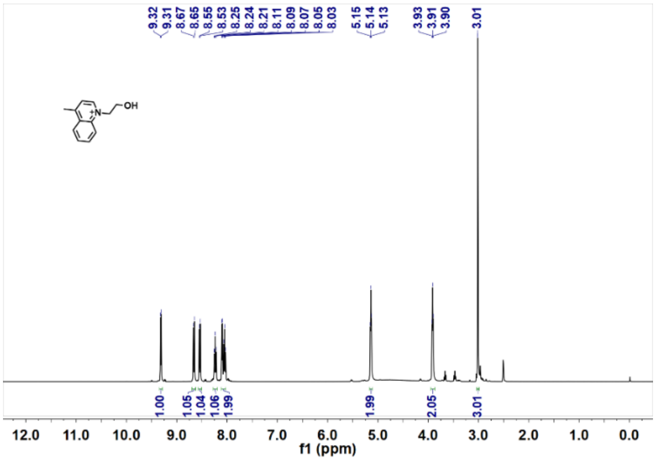

Supplementary Figure | ^{1}H-NMR spectrum of QL-EtOH in DMSO-d_6.

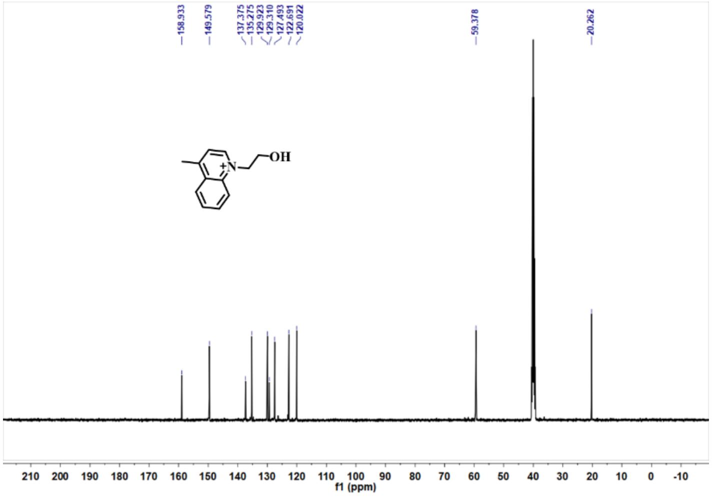

Supplementary Figure | ^{13}C-NMR spectrum of QL-EtOH in DMSO-d_6.

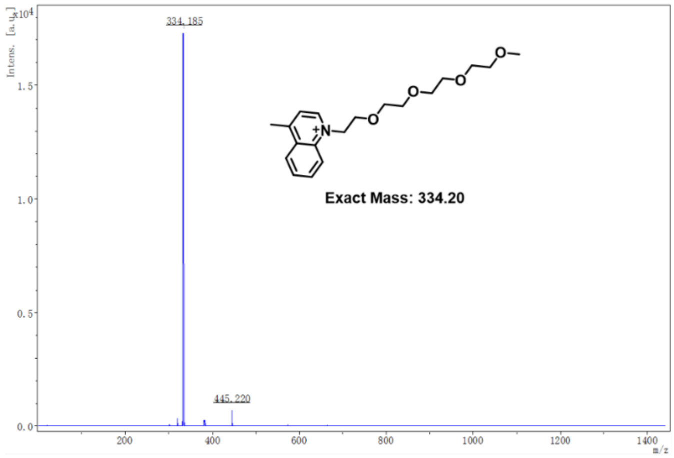

Supplementary Figure | MALDI-TOF mass spectrum of QL-PEG.

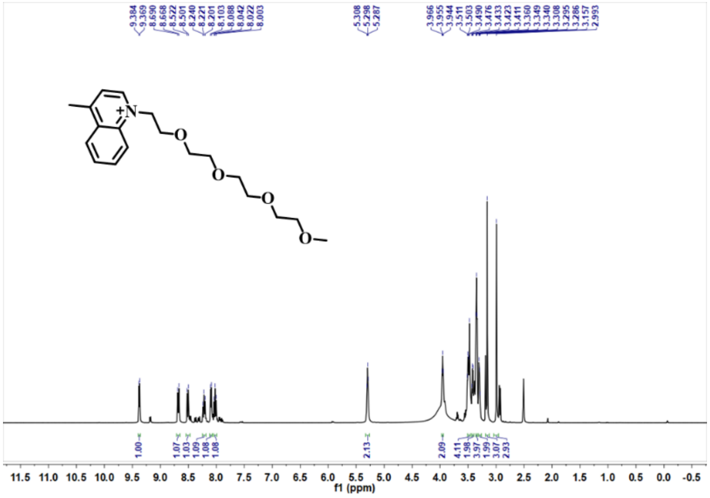

Supplementary Figure | ^{1}H-NMR spectrum of QL-PEG in DMSO-d_6.

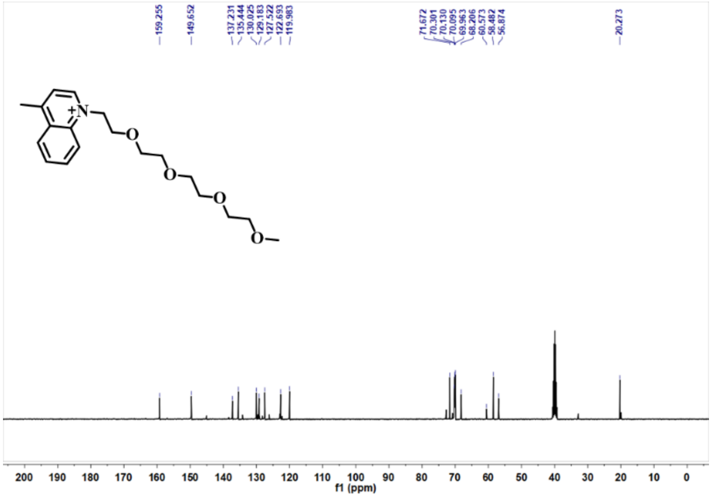

Supplementary Figure | ^{13}C-NMR spectrum of QL-PEG in DMSO-d_6.

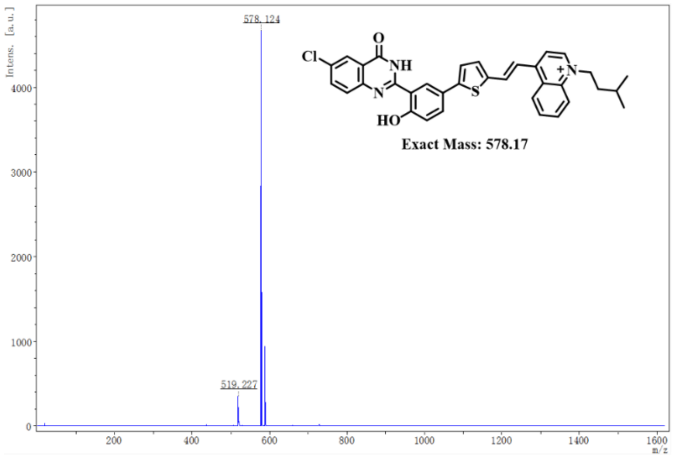

Supplementary Figure | MALDI-TOF mass spectrum of HPQ-i5C.

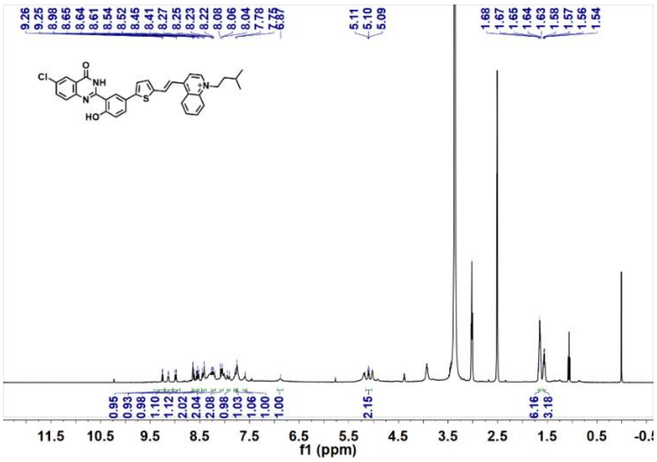

Supplementary Figure | ^{1}H-NMR spectrum of HPQ-i5C in DMSO-d_6.

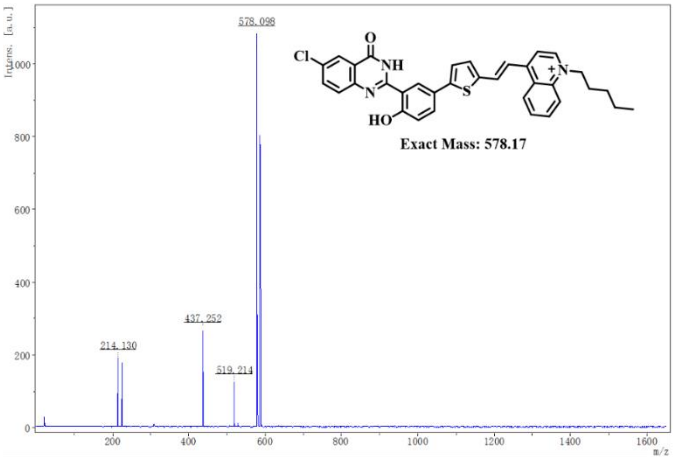

Supplementary Figure | MALDI-TOF mass spectrum of HPQ-n5C.

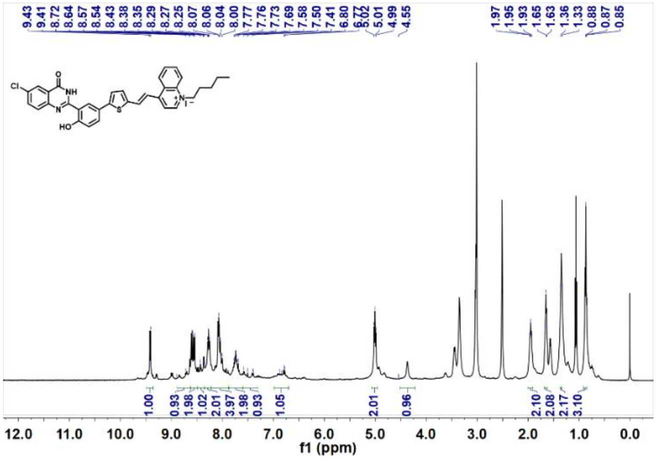

Supplementary Figure | ^{1}H-NMR spectrum of HPQ-n5C in DMSO-d_6.

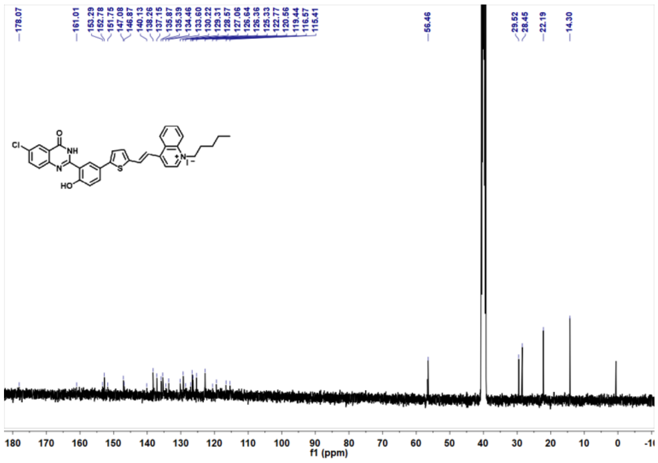

Supplementary Figure | ^{13}C-NMR spectrum of HPQ-n5C in DMSO-d_6.

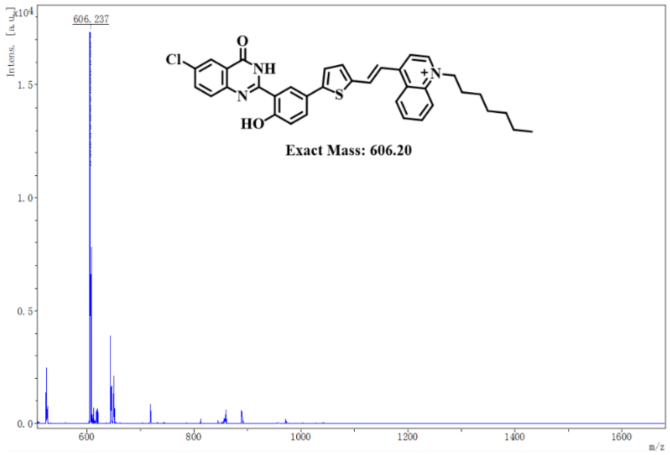

Supplementary Figure | MALDI-TOF mass spectrum of HPQ-n7C.

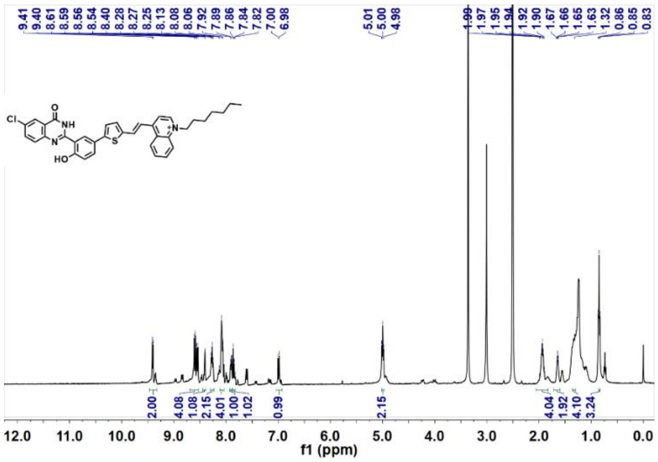

Supplementary Figure | ^{1}H-NMR spectrum of HPQ-n7C in DMSO-d_6.

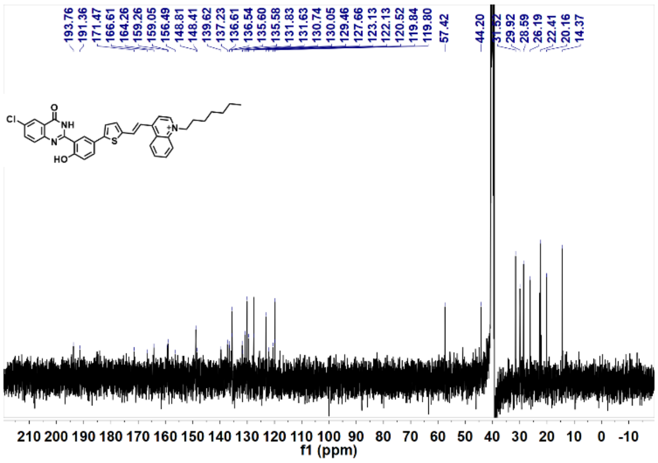

Supplementary Figure | ^{13}C-NMR spectrum of HPQ-n7C in DMSO-d_6.

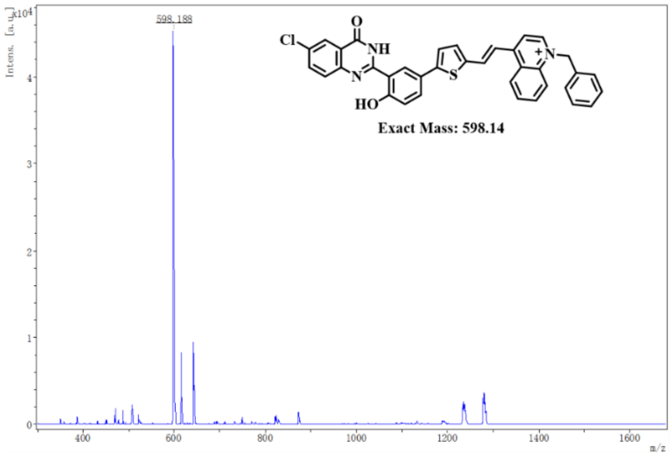

Supplementary Figure | MALDI-TOF mass spectrum of HPQ-Ph.

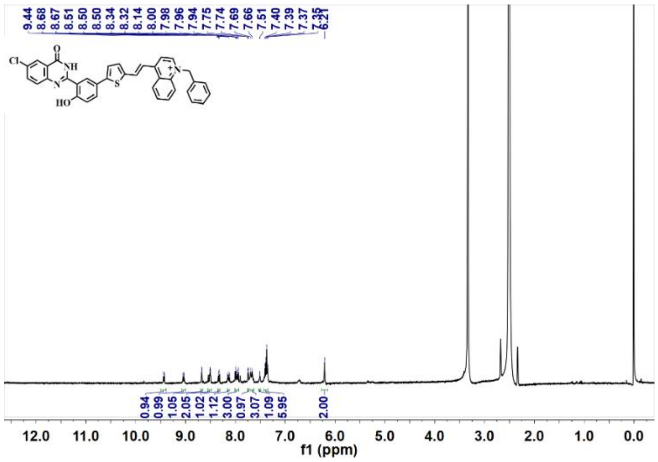

Supplementary Figure | ^{1}H-NMR spectrum of HPQ-Ph in DMSO-d_6.

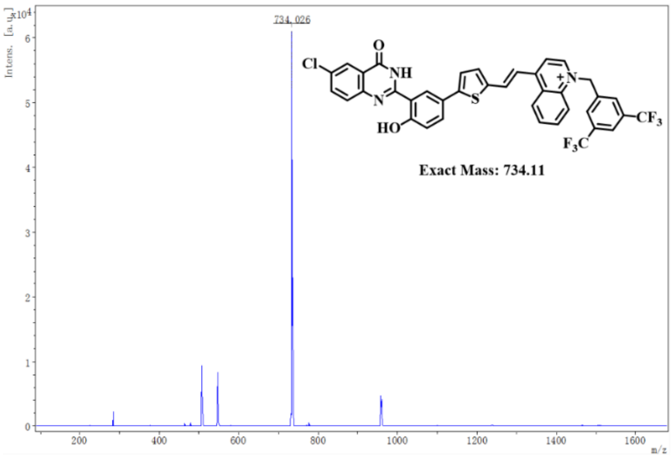

Supplementary Figure | MALDI-TOF mass spectrum of HPQ-Zzh.

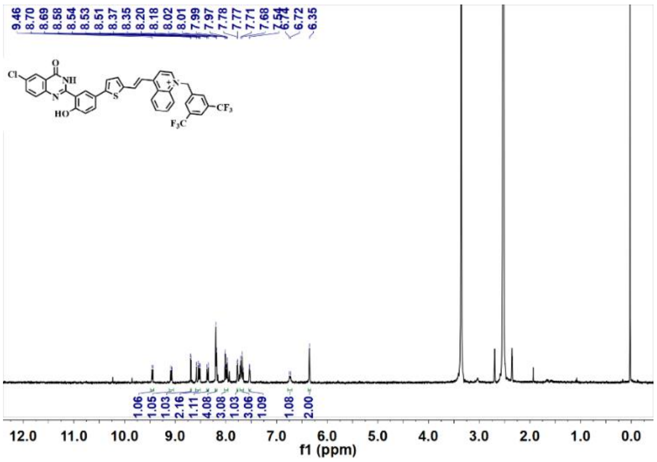

Supplementary Figure | ^{1}H-NMR spectrum of HPQ-Zzh in DMSO-d_6.

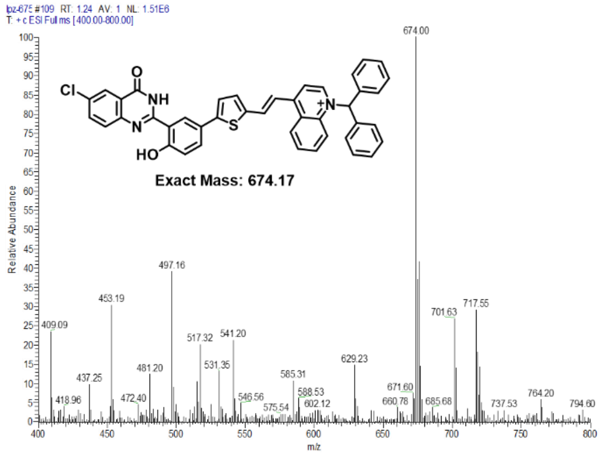

Supplementary Figure | ESI mass spectrum of HPQ-LPZ.

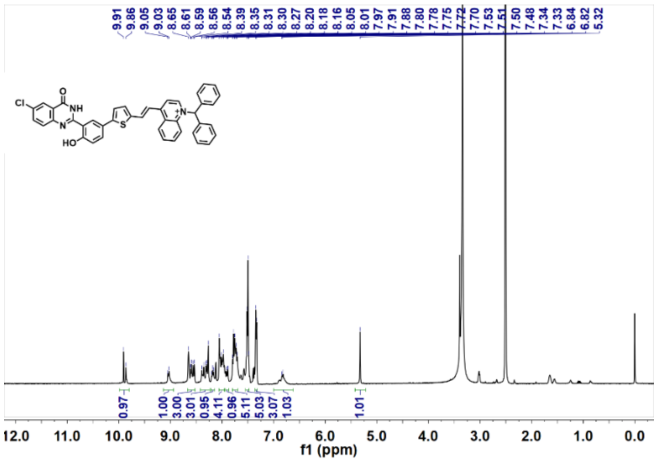

Supplementary Figure | ^{1}H-NMR spectrum of HPQ-LPZ in DMSO-d_6.

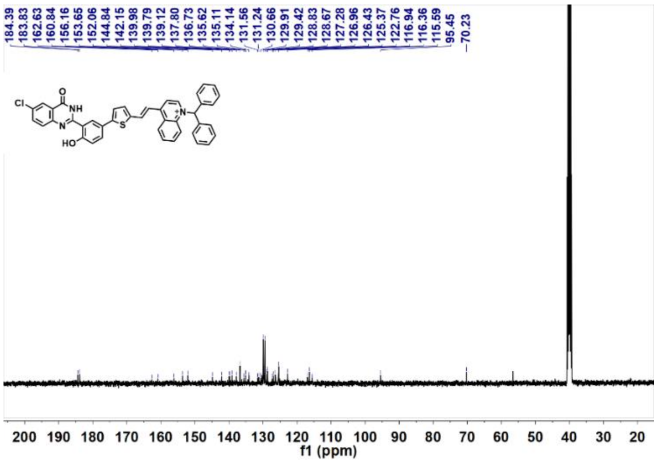

Supplementary Figure | ^{13}C-NMR spectrum of HPQ-LPZ in DMSO-d_6.

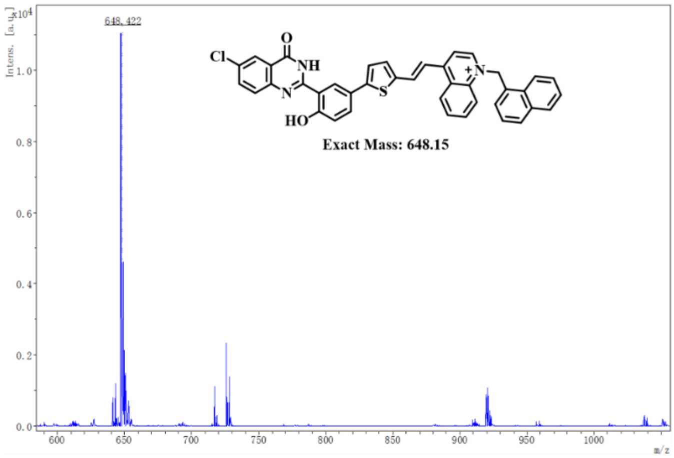

Supplementary Figure | MALDI-TOF mass spectrum of HPQ-1-Naph.

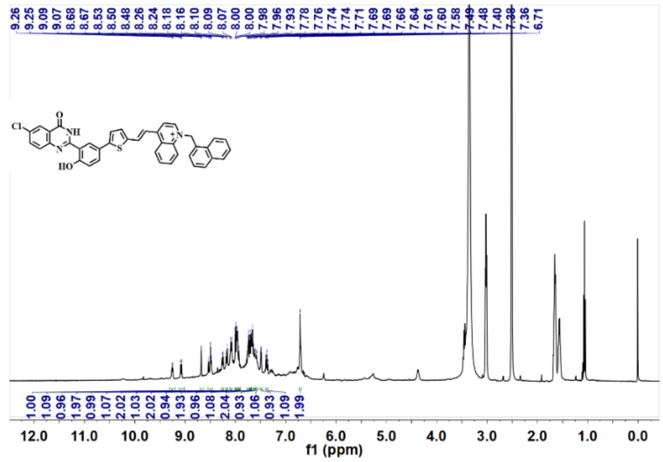

Supplementary Figure | ^{1}H-NMR spectrum of HPQ-1-Naph in DMSO-d_6.

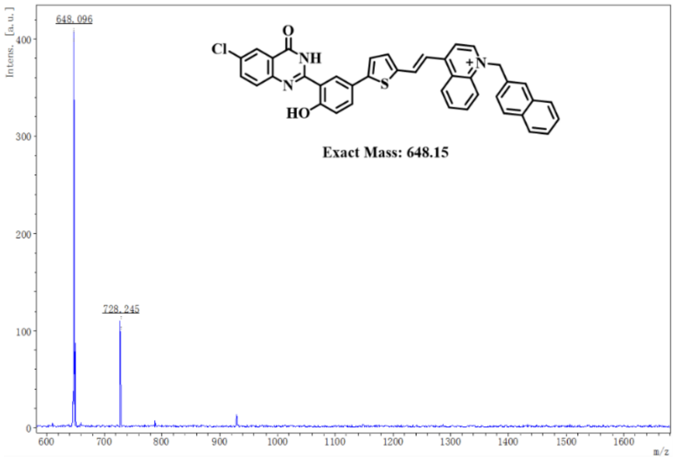

Supplementary Figure | MALDI-TOF mass spectrum of HPQ-2-Naph.

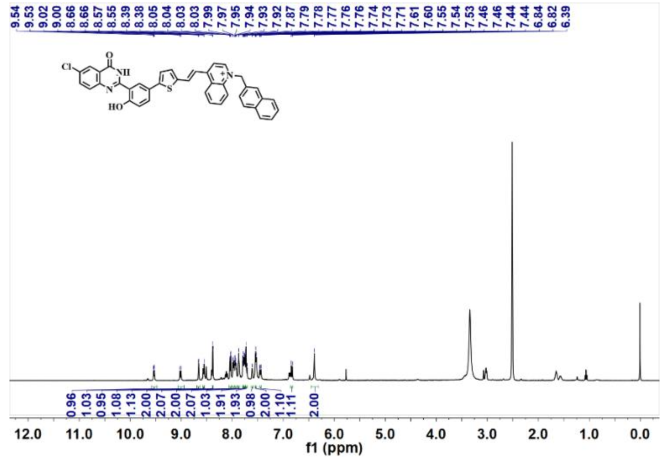

Supplementary Figure | ^{1}H-NMR spectrum of HPQ-2-Naph in DMSO-d_6.

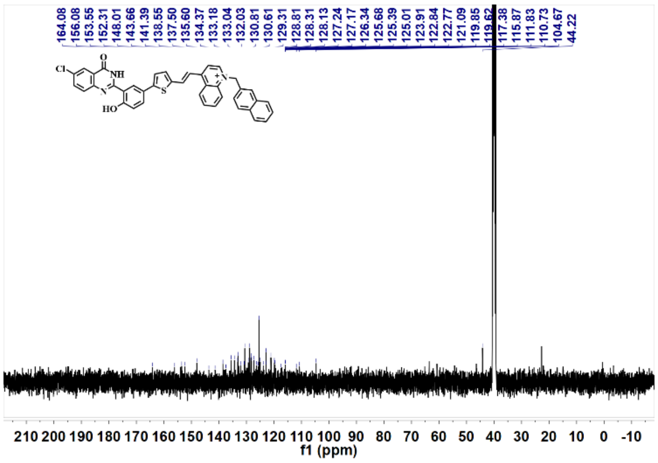

Supplementary Figure | ^{13}C-NMR spectrum of HPQ-2-Naph in DMSO-d_6.

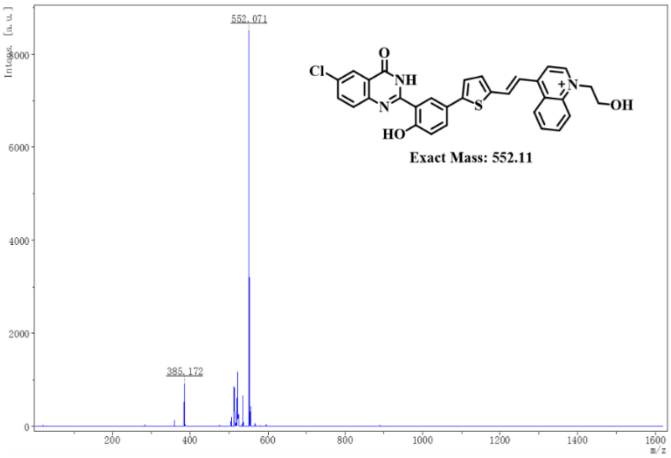

Supplementary Figure | MALDI-TOF mass spectrum of HPQ-EtOH.

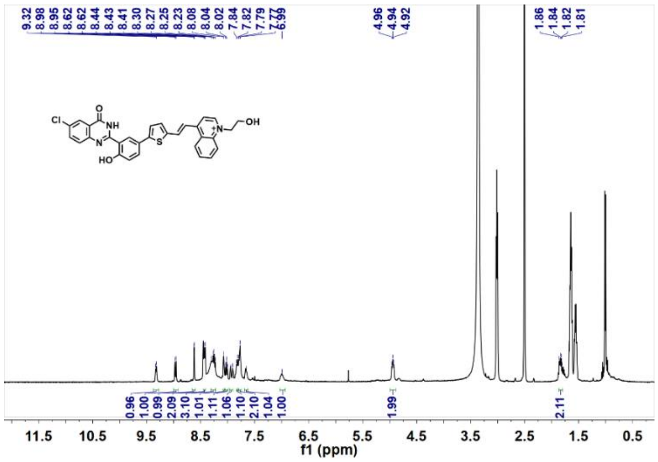

Supplementary Figure | ^{1}H-NMR spectrum of HPQ-EtOH in DMSO-d_6.

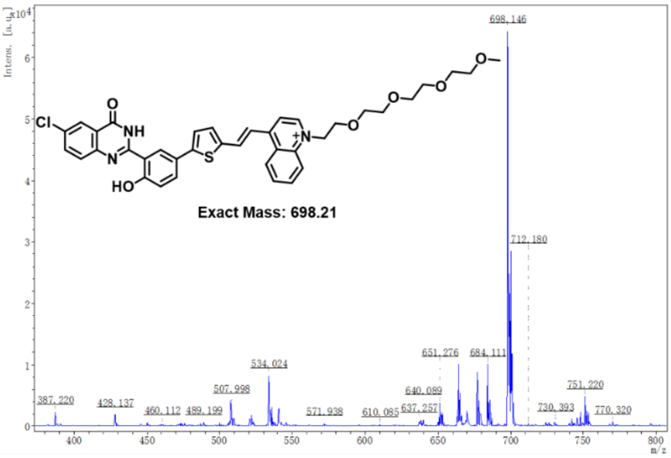

Supplementary Figure | MALDI-TOF mass spectrum of HPQ-PEG.

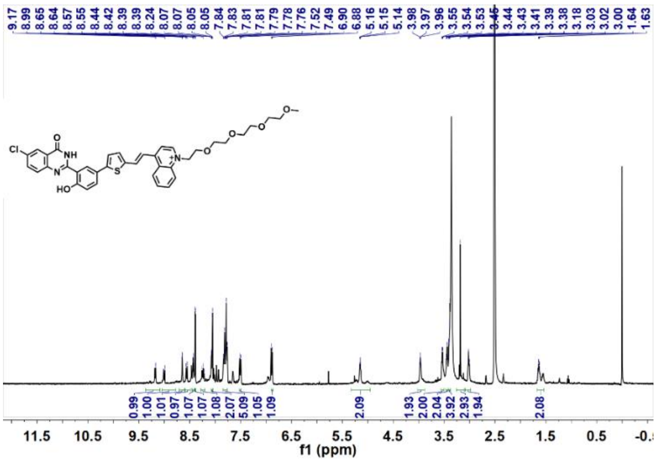

Supplementary Figure | ^{1}H-NMR spectrum of HPQ-PEG in DMSO-d_6.

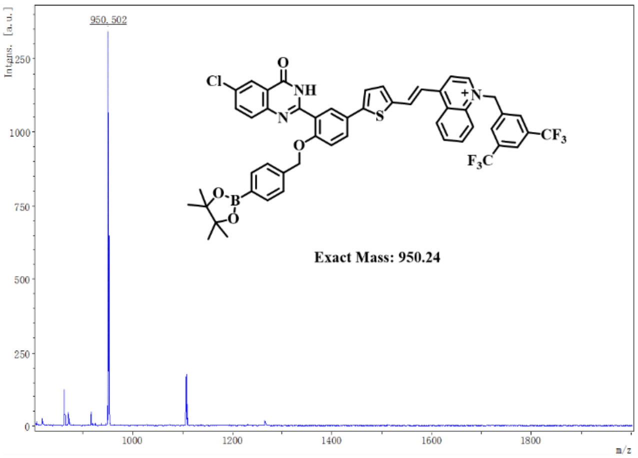

Supplementary Figure | MALDI-TOF mass spectrum of HPQ-Zzh-B.

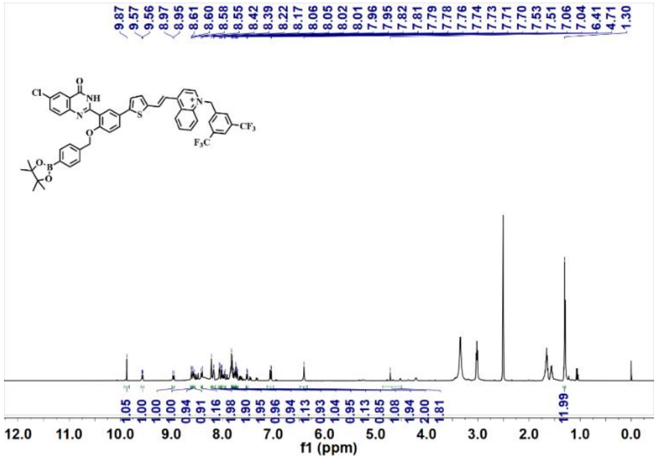

Supplementary Figure | ^{1}H-NMR spectrum of HPQ-Zzh-B in DMSO-d_6.

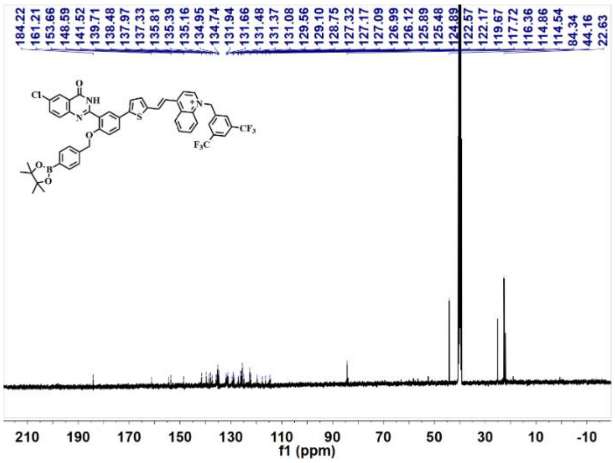

Supplementary Figure | ^{13}C-NMR spectrum of HPQ-Zzh-B in DMSO-d_6.

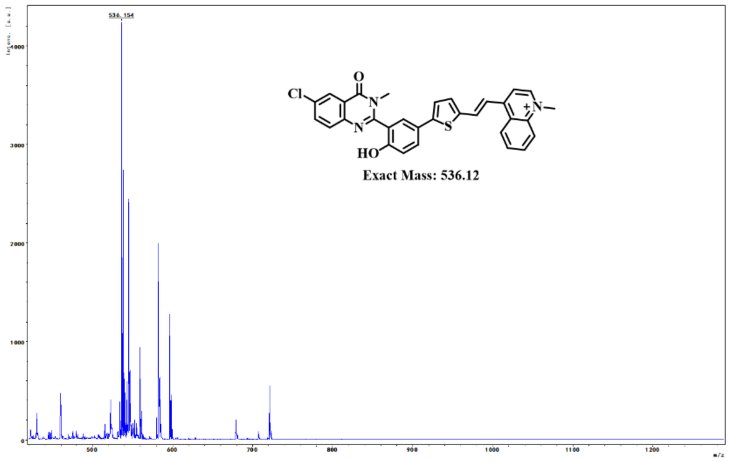

Supplementary Figure | MALDI-TOF mass spectrum of HPQ-LZ-Me.

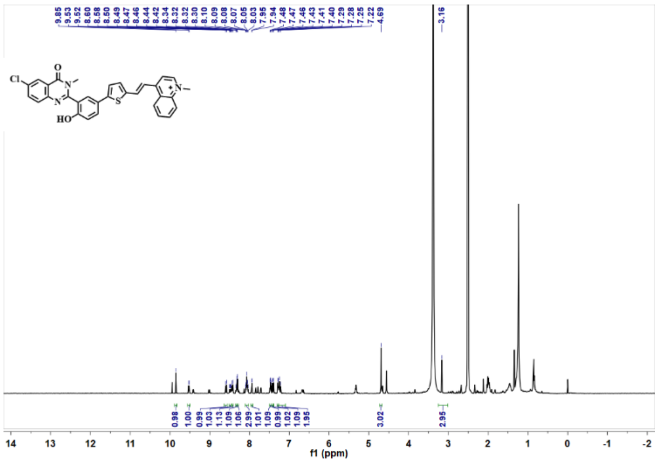

Supplementary Figure | ^{1}H-NMR spectrum of HPQ-LZ-Me in DMSO-d_6.

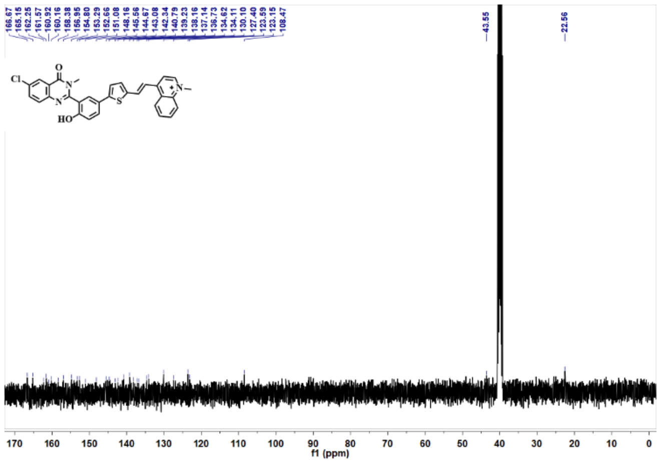

Supplementary Figure | ^{13}C-NMR spectrum of HPQ-LZ-Me in DMSO-d_6.

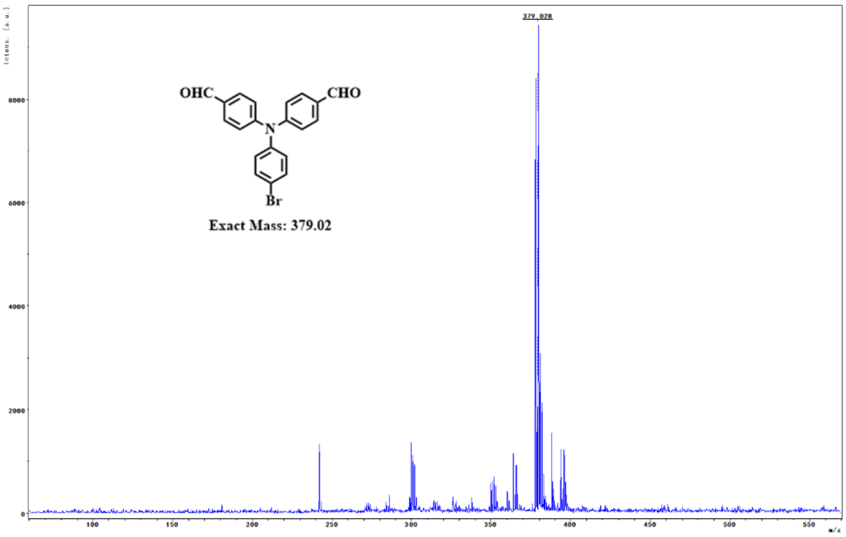

Supplementary Figure | MALDI-TOF mass spectrum of compound 17.

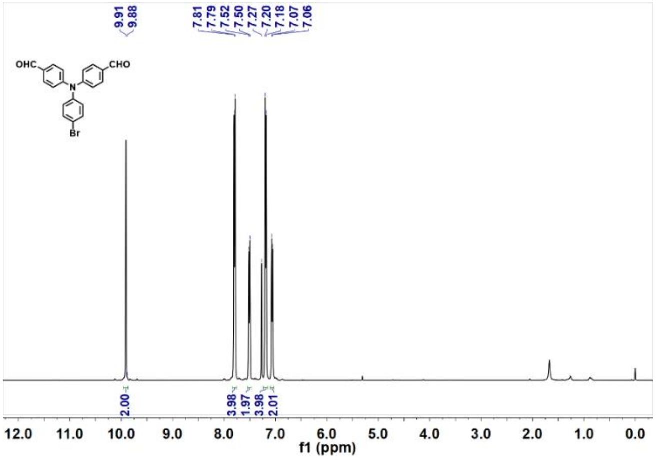

Supplementary Figure | ^{1}H-NMR spectrum of compound 17 in $CDCl_3$.

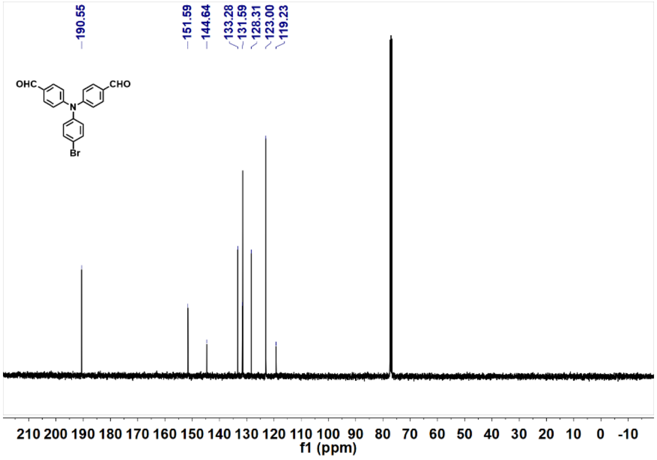

Supplementary Figure | ^{13}C-NMR spectrum compound 17 in $CDCl_3$.

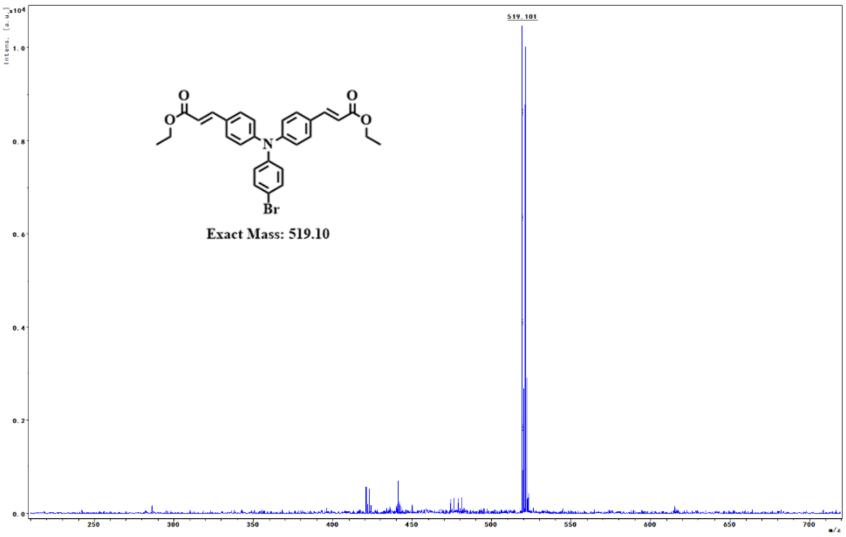

Supplementary Figure | MALDI-TOF mass spectrum of compound 18.

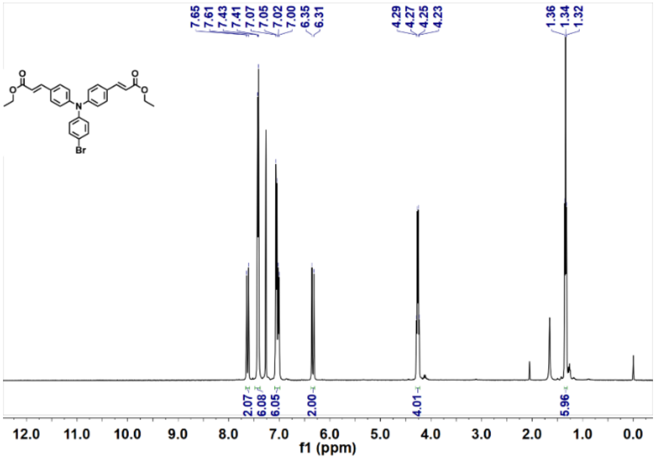

Supplementary Figure | ^{1}H-NMR spectrum compound 18 in $CDCl_3$.

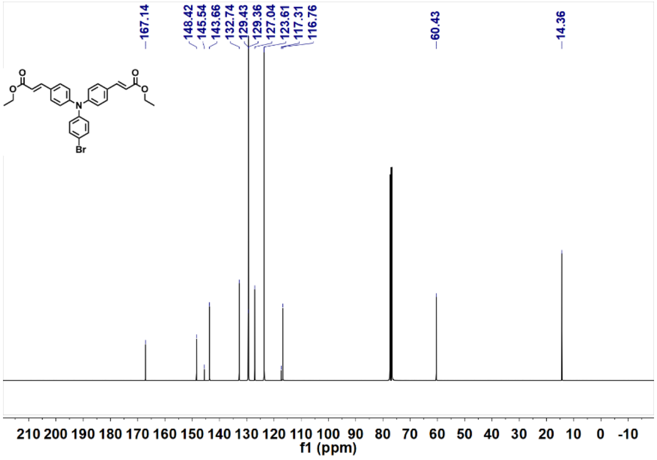

Supplementary Figure | ^{13}C-NMR spectrum compound 18 in $CDCl_3$.

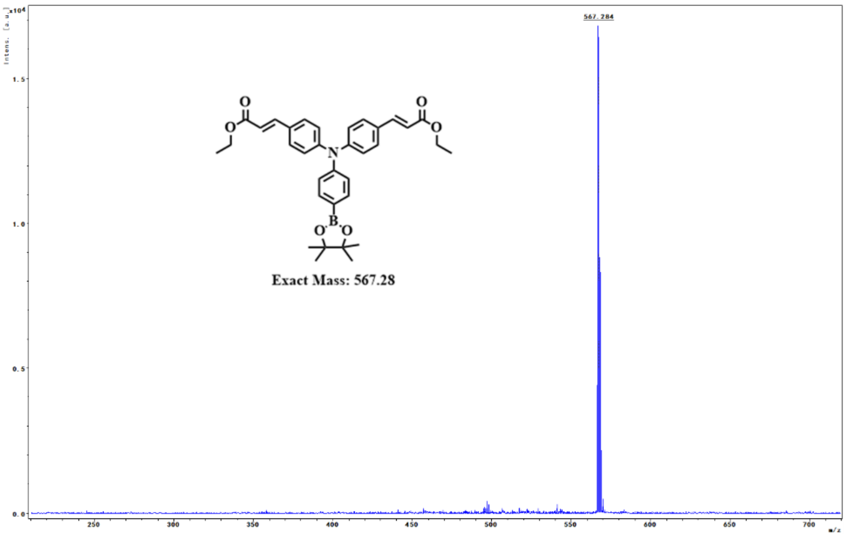

Supplementary Figure | MALDI-TOF mass spectrum of compound 19.

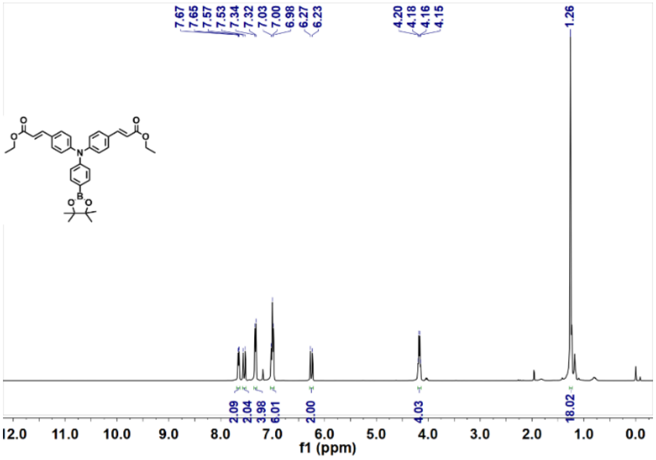

Supplementary Figure | ^{1}H-NMR spectrum compound 19 in $CDCl_3$.

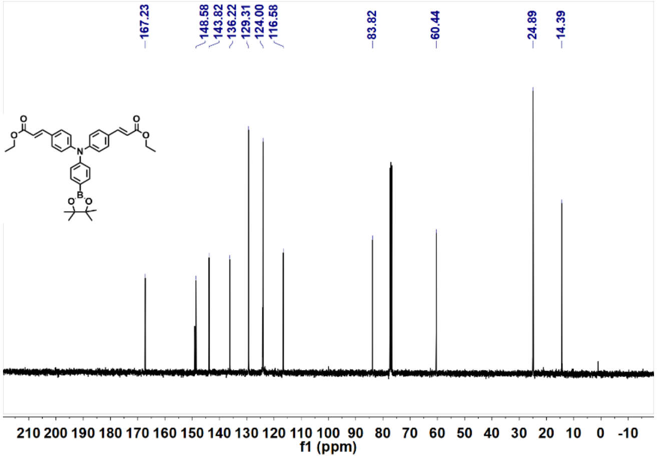

Supplementary Figure | ^{13}C-NMR spectrum compound 19 in $CDCl_3$.

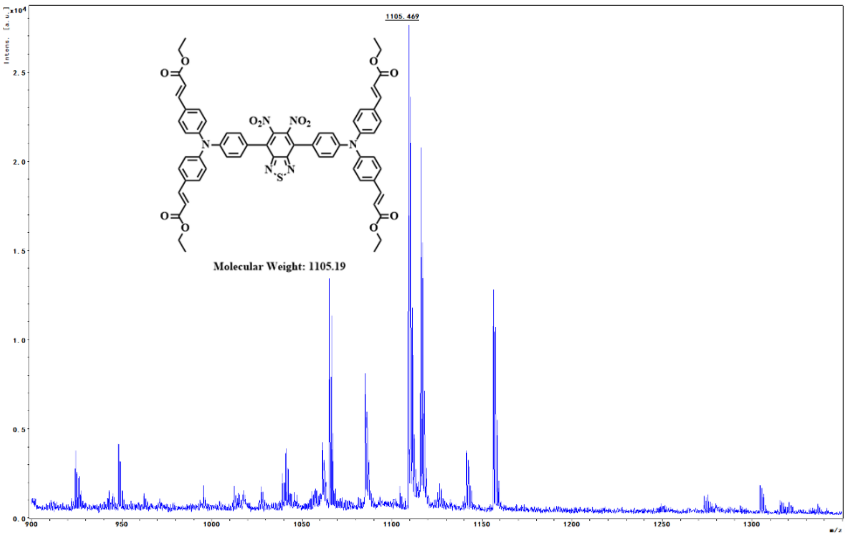

Supplementary Figure | MALDI-TOF mass spectrum of compound 22.

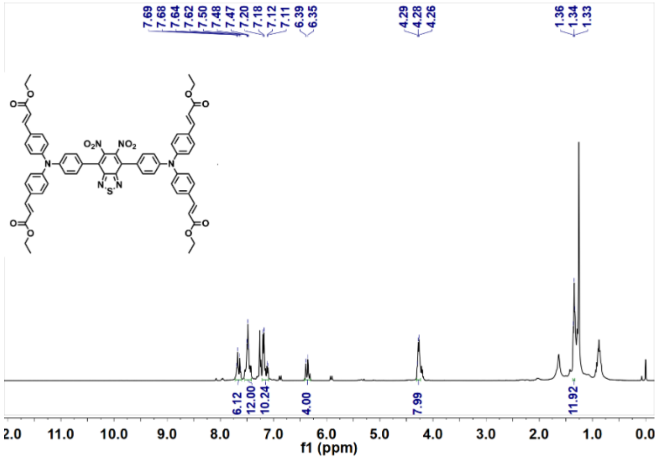

Supplementary Figure | ^{1}H-NMR spectrum compound 22 in $CDCl_3$.

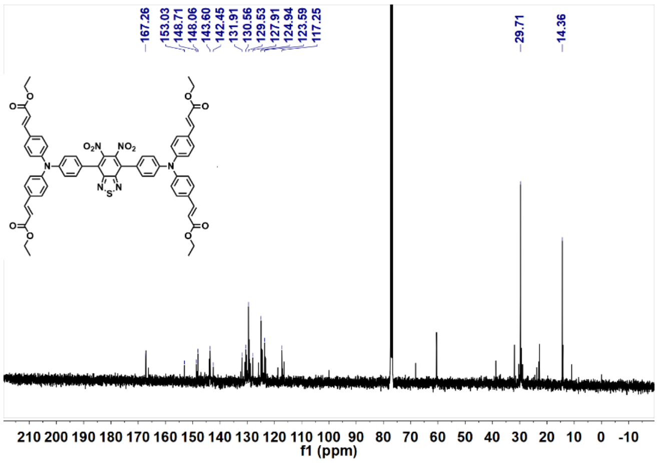

Supplementary Figure | ^{13}C-NMR spectrum compound 22 in $CDCl_3$.

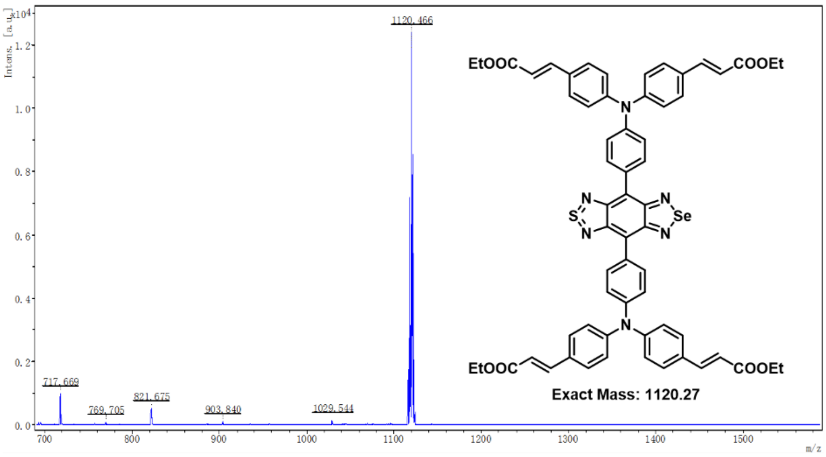

Supplementary Figure | MALDI-TOF mass spectrum of DAD-740.

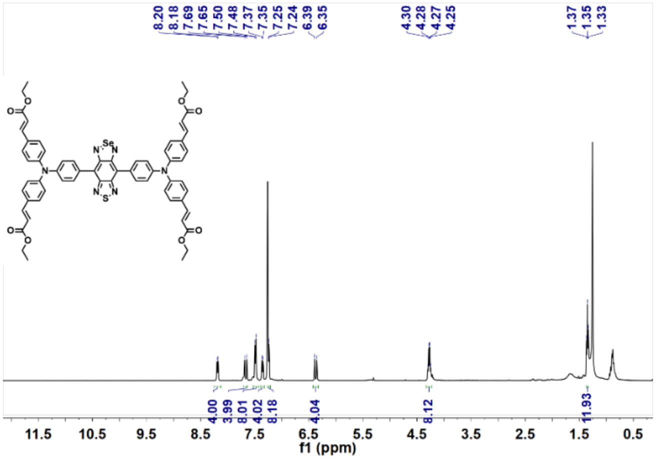

Supplementary Figure | ^{1}H-NMR spectrum of DAD-740 in $CDCl_3$.

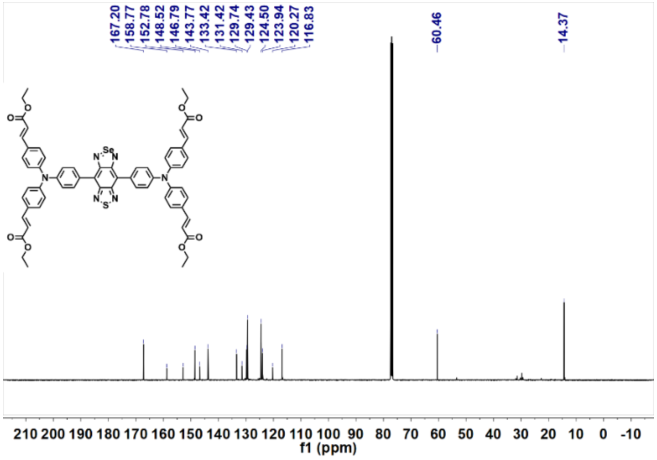

Supplementary Figure | ^{13}C-NMR spectrum of DAD-740 in $CDCl_3$.

5. Supplementary References

1. Cosco, E.D. et al. Flavylium Polymethine Fluorophores for Near- and Shortwave Infrared Imaging. *Angew. Chem. Int. Ed.* **56**, 13126-13129 (2017).

2. Sun, C. et al. J-Aggregates of Cyanine Dye for NIR-II in Vivo Dynamic Vascular Imaging beyond 1500 nm. *J. Am. Chem. Soc.* **141**, 19221-19225 (2019).
