# Supplementary material for: In situ orderly self-assembly strategy affording NIR-II-J-aggregates for in vivo imaging and surgical navigation
Source: Nat Commun. 2023 Apr 3;14:1843. doi: 10.1038/s41467-023-37586-7 (PMC10070396; doi:10.1038/s41467-023-37586-7)
Supplement: Supplementary file 3 — Description of Additional Supplementary Files [file 41467_2023_37586_MOESM3_ESM.pdf]

File Name: Supplementary Movie 1

Description: Surgical navigation for subcutaneous tumor resection based on NIR-II fluorescence.
