# Supplementary material for: In situ orderly self-assembly strategy affording NIR-II-J-aggregates for in vivo imaging and surgical navigation
Source: Nat Commun. 2023 Apr 3;14:1843. doi: 10.1038/s41467-023-37586-7 (PMC10070396; doi:10.1038/s41467-023-37586-7)
Supplement: Supplementary file 6 — Reporting Summary [file 41467_2023_37586_MOESM6_ESM.pdf]

nature portfolio

Corresponding author(s): Xiao-Bing Zhang

Last updated by author(s): Feb 1, 2023

Reporting Summary

Nature Portfolio wishes to improve the reproducibility of the work that we publish. This form provides structure for consistency and transparency in reporting. For further information on Nature Portfolio policies, see our Editorial Policies and the Editorial Policy Checklist.

Statistics

For all statistical analyses, confirm that the following items are present in the figure legend, table legend, main text, or Methods section.

n/a	Confirmed	
☐	☒	The exact sample size (*n*) for each experimental group/condition, given as a discrete number and unit of measurement
☐	☒	A statement on whether measurements were taken from distinct samples or whether the same sample was measured repeatedly
☐	☒	The statistical test(s) used AND whether they are one- or two-sided<br>*Only common tests should be described solely by name; describe more complex techniques in the Methods section.*
☐	☒	A description of all covariates tested
☐	☒	A description of any assumptions or corrections, such as tests of normality and adjustment for multiple comparisons
☐	☒	A full description of the statistical parameters including central tendency (e.g. means) or other basic estimates (e.g. regression coefficient) AND variation (e.g. standard deviation) or associated estimates of uncertainty (e.g. confidence intervals)
☒	☐	For null hypothesis testing, the test statistic (e.g. *F*, *t*, *r*) with confidence intervals, effect sizes, degrees of freedom and *P* value noted<br>*Give P values as exact values whenever suitable.*
☒	☐	For Bayesian analysis, information on the choice of priors and Markov chain Monte Carlo settings
☒	☐	For hierarchical and complex designs, identification of the appropriate level for tests and full reporting of outcomes
☒	☐	Estimates of effect sizes (e.g. Cohen's *d*, Pearson's *r*), indicating how they were calculated

Our web collection on statistics for biologists contains articles on many of the points above.

Software and code

Policy information about availability of computer code

Data collection	UV–Vis spectra were recorded on a UV-1800 spectrophotometer (Shimadzu Corporation, Japan). Photoluminescence spectra were recorded on an Edinburgh Instruments FLS-1000 fluorescence spectrometer. All NIR-II images were collected on a home-built small animal imaging system with 640×512 pixel 2D InGaAs NIRvana CCD camera. Dynamic light scattering (DLS) was measured on Malvern Zetasizer Nano ZS90 (Malvern). Mass spectra were performed using an Agilent 1200-6520 Q-TOF mass spectrometer system operating in a MALDI-TOF mode and LCQ Advantage ion trap mass spectrometer (Thermo Finnigan). NMR spectra were recorded on Bruker-400.
Data analysis	All statistical graph, absorption spectrum and fluorescent spectra were analyzed with OriginLab 2019. NIR-II Images were processed with the LightField imaging software and Image J (1.4.3.67). NMR files were analyzed with MestReNova. Mass spectrum files were analyzed with flexAnalysis.

For manuscripts utilizing custom algorithms or software that are central to the research but not yet described in published literature, software must be made available to editors and reviewers. We strongly encourage code deposition in a community repository (e.g. GitHub). See the Nature Portfolio guidelines for submitting code & software for further information.

Data

Policy information about availability of data

All manuscripts must include a data availability statement. This statement should provide the following information, where applicable:

- Accession codes, unique identifiers, or web links for publicly available datasets
- A description of any restrictions on data availability
- For clinical datasets or third party data, please ensure that the statement adheres to our policy

The source data underlying Figs. 3, 4d-i, 5b-g, 7h–i and Supplementary Figs. 1-4, 6, 9, 10, 12–22, 24, 26, 29 are recorded in a Source Data file. The authors declare that other data related to this research are available within the paper and its Supplementary Information, or from the authors upon request.

Human research participants

Policy information about studies involving human research participants and Sex and Gender in Research.

Reporting on sex and gender	*Use the terms sex (biological attribute) and gender (shaped by social and cultural circumstances) carefully in order to avoid confusing both terms. Indicate if findings apply to only one sex or gender; describe whether sex and gender were considered in study design whether sex and/or gender was determined based on self-reporting or assigned and methods used. Provide in the source data disaggregated sex and gender data where this information has been collected, and consent has been obtained for sharing of individual-level data; provide overall numbers in this Reporting Summary. Please state if this information has not been collected. Report sex- and gender-based analyses where performed, justify reasons for lack of sex- and gender-based analysis.*
Population characteristics	*Describe the covariate-relevant population characteristics of the human research participants (e.g. age, genotypic information, past and current diagnosis and treatment categories). If you filled out the behavioural & social sciences study design questions and have nothing to add here, write "See above."*
Recruitment	*Describe how participants were recruited. Outline any potential self-selection bias or other biases that may be present and how these are likely to impact results.*
Ethics oversight	*Identify the organization(s) that approved the study protocol.*

Note that full information on the approval of the study protocol must also be provided in the manuscript.

Field-specific reporting

Please select the one below that is the best fit for your research. If you are not sure, read the appropriate sections before making your selection.

[x] Life sciences [ ] Behavioural & social sciences [ ] Ecological, evolutionary & environmental sciences

For a reference copy of the document with all sections, see nature.com/documents/nr-reporting-summary-flat.pdf

Life sciences study design

All studies must disclose on these points even when the disclosure is negative.

Sample size	To ensure high repeatability and consistency of experiment, it was predetermined that a sample size of at least n=3 would allow for adequate analysis to reach meaningful conclusions of the data.
Data exclusions	No data was excluded.
Replication	All experiments have been reproduced to reliably support the conclusions stated in the manuscript (at least three replicates).
Randomization	All samples/organism were divided into each group randomly.
Blinding	No blinding was used throughout experiments. All data collected was quantifiable and blinding would not change any bias in data collected.

Reporting for specific materials, systems and methods

We require information from authors about some types of materials, experimental systems and methods used in many studies. Here, indicate whether each material, system or method listed is relevant to your study. If you are not sure if a list item applies to your research, read the appropriate section before selecting a response.

Materials & experimental systems

n/a	Involved in the study	
☐	☒	Antibodies
☐	☒	Eukaryotic cell lines
☒	☐	Palaeontology and archaeology
☐	☒	Animals and other organisms
☒	☐	Clinical data
☒	☐	Dual use research of concern

Methods

n/a	Involved in the study	
☒	☐	ChIP-seq
☒	☐	Flow cytometry
☒	☐	MRI-based neuroimaging

Antibodies

Antibodies used	CD206 antibodies were purchased from Wuhan Servicebio Technology CO., LTD.<br>Description: Mannose Receptor rabbit polyclonal.<br>Protein full name: Macrophage mannose receptor 1.<br>Synonyms: CD206, CLEC13D, Macrophage mannose receptor 1, Mannose receptor, mannose receptor, C type 1, MMR, MRC1.<br>Immunogen: KLH conjugated Synthetic peptide corresponding to Mouse Mannose Receptor.<br>Isotype: IgG.<br>Purity: Affinity purification.<br>Subcellular location: Cell membrane, Endosome.<br>Uniprot ID: Q61830.<br>Product number: GB113497
Validation	All antibodies were used in the research according to the manufacturer's information.<br>CD206, also named as MMR, CLEC13D and MRC1, is a type I membrane receptor that mediates the endocytosis of glycoproteins by macrophages. CD206 has been shown to bind high-mannose structures on the surface of potentially pathogenic viruses, bacteria, and fungi so that they can be neutralized by phagocytic engulfment. CD206 is a 170 kDa transmembrane glycoprotein which contains 5 domains: an amino-terminal cysteine-rich region, a fibronectin type II repeat, a series of eight tandem lectin-like carbohydrate recognition domains (responsible for the recognition of mannose and fucose), a transmembrane domain, and an intracellular carboxy-terminal tail.

Eukaryotic cell lines

Policy information about cell lines and Sex and Gender in Research

Cell line source(s)	All the cells lines (4T1) were purchased from American Type Culture Collection (ATCC).
Authentication	The cell line was purchased directly from ATCC without independent verification.
Mycoplasma contamination	The cell line was not tested for mycoplasma contamination.
Commonly misidentified lines<br>(See ICLAC register)	No commonly misidentified cell lines were used.

Animals and other research organisms

Policy information about studies involving animals; ARRIVE guidelines recommended for reporting animal research, and Sex and Gender in Research

Laboratory animals	Mice (8-week-old) were purchased from Hunan Slake Jingda Laboratory Animal Co., Ltd (China). Mice were housed under controlled conditions (22 °C, 55 - 65% humidity, 12h light-dark cycle) and were allowed free access to tap water.
Wild animals	The study did not involve wild animals
Reporting on sex	In this manuscript, our animal studies focused on NIR-II fluorescence imaging and surgical resection of tumour, therefore, we did not perform sex-based analyses.
Field-collected samples	The study did not involve samples collected from the field
Ethics oversight	All animal experiments were conducted in accordance with the Guidelines for the Care and Use of Laboratory Animals of Hunan University, and experiments were approved by the Animal Ethics Committee of the College of Biology (Hunan University).

Note that full information on the approval of the study protocol must also be provided in the manuscript.
